# Assessment of fluid responsiveness using pulse pressure variation, stroke volume variation, plethysmographic variability index, central venous pressure, and inferior vena cava variation in patients undergoing mechanical ventilation: a systematic review and meta-analysis

**DOI:** 10.1186/s13054-024-05078-9

**Published:** 2024-08-31

**Authors:** Renato Carneiro de Freitas Chaves, Carmen Silvia Valente Barbas, Veronica Neves Fialho Queiroz, Ary Serpa Neto, Rodrigo Octavio Deliberato, Adriano José Pereira, Karina Tavares Timenetsky, João Manoel Silva Júnior, Flávio Takaoka, Daniel de Backer, Leo Anthony Celi, Thiago Domingos Corrêa

**Affiliations:** 1 Department of Intensive Care, Hospital Israelita Albert Einstein, São Paulo, SP Brazil; 2 Department of Anesthesiology, Hospital Israelita Albert Einstein, São Paulo, SP Brazil; 3 Department of Pneumology, Instituto do Coração (INCOR), Hospital das Clínicas da Faculdade de Medicina da Universidade de São Paulo, São Paulo, Brazil; 4 MIT Critical Data, Laboratory for Computational Physiology, Harvard-MIT Health Sciences and Technology, Massachusetts Institute of Technology, Cambridge, MA USA; 5 Department of Anesthesiology, Takaoka Anestesia, São Paulo, SP Brazil; 6 Australian and New Zealand Intensive Care Research Centre (ANZIC-RC), Melbourne, VIC Australia; 7 Department of Intensive Care, Melbourne Medical School, University of Melbourne, Austin Hospital, Melbourne, Australia; 8 Translational Health Intelligence and Knowledge Lab, Department of Biostatistics, Health Informatics and Data Science, University of Cincinnati, Cincinnati, OH USA; 9 Division of Biomedical Informatics, Cincinnati Children’s Hospital, Cincinnati, OH USA; 10 Department of Intensive Care, CHIREC Hospitals, Université Libre de Bruxelles, Brussels, Belgium; 11 Department of Critical Care Medicine and Anesthesiology, Hospital Israelita Albert Einstein, Avenida Albert Einstein, 627/701, 5° Floor, São Paulo, SP 05651-901 Brazil

**Keywords:** Hemodynamic, Cardiac output, Echocardiography, Intensive care, Anesthesiology

## Abstract

**Importance:**

Maneuvers assessing fluid responsiveness before an intravascular volume expansion may limit useless fluid administration, which in turn may improve outcomes.

**Objective:**

To describe maneuvers for assessing fluid responsiveness in mechanically ventilated patients.

**Registration:**

The protocol was registered at PROSPERO: CRD42019146781.

**Information sources and search:**

PubMed, EMBASE, CINAHL, SCOPUS, and Web of Science were search from inception to 08/08/2023.

**Study selection and data collection:**

Prospective and intervention studies were selected**.**

**Statistical analysis:**

Data for each maneuver were reported individually and data from the five most employed maneuvers were aggregated. A traditional and a Bayesian meta-analysis approach were performed.

**Results:**

A total of 69 studies, encompassing 3185 fluid challenges and 2711 patients were analyzed. The prevalence of fluid responsiveness was 49.9%. Pulse pressure variation (PPV) was studied in 40 studies, mean threshold with 95% confidence intervals (95% CI) = 11.5 (10.5–12.4)%, and area under the receiver operating characteristics curve (AUC) with 95% CI was 0.87 (0.84–0.90). Stroke volume variation (SVV) was studied in 24 studies, mean threshold with 95% CI = 12.1 (10.9–13.3)%, and AUC with 95% CI was 0.87 (0.84–0.91). The plethysmographic variability index (PVI) was studied in 17 studies, mean threshold = 13.8 (12.3–15.3)%, and AUC was 0.88 (0.82–0.94). Central venous pressure (CVP) was studied in 12 studies, mean threshold with 95% CI = 9.0 (7.7–10.1) mmHg, and AUC with 95% CI was 0.77 (0.69–0.87). Inferior vena cava variation (∆IVC) was studied in 8 studies, mean threshold = 15.4 (13.3–17.6)%, and AUC with 95% CI was 0.83 (0.78–0.89).

**Conclusions:**

Fluid responsiveness can be reliably assessed in adult patients under mechanical ventilation. Among the five maneuvers compared in predicting fluid responsiveness, PPV, SVV, and PVI were superior to CVP and ∆IVC. However, there is no data supporting any of the above mentioned as being the best maneuver. Additionally, other well-established tests, such as the passive leg raising test, end-expiratory occlusion test, and tidal volume challenge, are also reliable.

**Supplementary Information:**

The online version contains supplementary material available at 10.1186/s13054-024-05078-9.

## Introduction

Fluid therapy is one of the cornerstones of hemodynamic resuscitation [1, 2]. While fluids may have beneficial effect, excessive fluid administration may contribute to fluid accumulation, which has been associated with adverse events and poor clinical outcomes [3, 4]. Optimization of fluid therapy implies restricting fluid administration to those patients who are predicted to respond to a fluid infusion in order to prevent useless and potentially harmful fluid administration [4, 5]. Accordingly, the assessment of fluid responsiveness prior to fluid administration sounds logical [6].

Fluid responsiveness is defined as the patient’s capacity to increase cardiac output (CO) in response to an intravenous (I.V.) fluid infusion [7, 8]. From a physiology point of view, patients who increase CO during an intravascular volume expansion have both ventricles in the ascending portion of the Frank–Starling curve, which characterizes preload responsiveness [7]. Despite this straightforward and objective definition, bedside identification of fluid responsiveness remains one of the most challenging tasks in critically ill patients [9].

The gold standard assessment of fluid responsiveness is to perform a fluid challenge and quantify the variation of CO, cardiac index (CI) or stroke volume (SV) before and after the infusion of a specific amount of intravenous fluid [7]. However, as many patients may fail to respond to fluids, it sounds logical to predict which patient may respond to fluid prior to fluid administration. Several maneuvers and tests to predict fluid responsiveness in mechanically ventilated patients have been described [1, 10]. Nevertheless, a significant variability in operational characteristics, such as cardiac arrhythmia, increased abdominal pressure, spontaneous breathing activity, need for pulmonary ventilation with low tidal volume and high positive end-expiratory pressure (PEEP), peripheral vascular disease, as well as costs, availability, and performances of CO monitoring (including poor echocardiographic echogenicity) may affect test selection [9, 10]. Well-established tests, such as the passive leg raising test, end-expiratory occlusion test, and tidal volume challenge, are also reliable [9, 10]. In addition to applicability and availability, each test has its own intrinsic discriminative performances that may affect the decision-making regarding what methods and threshold value should be used at the bedside [1, 10].

To address common issues in meta-analysis concerning fluid responsiveness, this meta-analysis performed a traditional and a Bayesian approach. The inclusion of a Bayesian approach can enhance the reliability of results by addressing two common issues in meta-analysis concerning fluid responsiveness: a limited number of studies describing methods for assessing fluid responsiveness and small sample sizes. The Bayesian approach provides more robust credible intervals, even in scenarios with a limited number of studies, and may help mitigate the influence of studies with relatively small sample sizes, which could introduce biases (small study effect) [11, 12].

Therefore, this systematic review and meta-analysis aimed to describe the diagnostic performance and summarize threshold values for five common maneuvers available to assess fluid responsiveness in mechanically ventilated patients. We compared the predictive value of the different tests.

## Methods

### Protocol and registration

This systematic review and meta-analysis of diagnostic test accuracy was conducted and reported according to the Preferred Reporting Items for Systematic Reviews and Meta-Analyses (PRISMA) guidelines [13], and the Cochrane Handbook for Diagnostic Test Accuracy Reviews [14]. The study protocol was registered at the International Prospective Register of systematic reviews (PROSPERO) on registration number CRD42019146781 [15]. Due to the reviewing nature of this study, institutional review board ethical approval was not needed.

### Eligibility criteria and study selection

Studies were selected according to the PICOS statement as follows:

- P-Patients and setting: studies were eligible for inclusion if they evaluated adult patients at the intensive care unit (ICU), emergency department, and operating room.
- I-index test: studies were eligible for inclusion if they evaluated maneuvers to assess fluid responsiveness in mechanically ventilated adult patients. All maneuvers to assess fluid responsiveness were eligible.
- C-comparison or reference standard: studies were assessed for eligibility if one of the following standard definitions of fluid responsiveness and fluid challenge was adopted: an increase in CO or CI or SV or stroke volume index (SVI) or velocity–time integral (VTI) ≥ 10% after a fluid challenge. A fluid challenge was considered adequate if at least 200 ml or 4 ml/kg of I.V. fluid (crystalloids or colloids) was infused within 15 min or 500 ml within 30 min. More than one fluid challenge could be performed in the same patient. Mechanical ventilation was defined as a modality of life support that delivers ventilation cycles with positive pressure to the lungs under controlled or assisted/controlled mode via a tube inserted into the trachea. Patients in spontaneous mode of mechanical ventilation or with respiratory movements were excluded.
- O-outcomes or target condition: to be selected, studies should report data on the operative performance of any fluid responsiveness test and at least the following parameters: the cutoff value of each maneuver to assess fluid responsiveness, the number of patients, the number of fluid challenges performed, the frequency of fluid responsiveness or non-fluid responsiveness patients, the adopted definition of fluid responsiveness, and the amount of I.V. fluid infused. If the study had multiple data points on operative performance; all data regarding operative performance were included.
- S-studies: prospective interventional studies were included. Review articles, editorials, comments, letters, case reports, animal studies, non-interventional studies, studies assessing fluid responsiveness during spontaneous breathing, studies that either did not report or did not provide information enabling the calculation of sensitivity and specificity, and studies that did not report outcomes of interest were excluded.

### Information sources and search

The completely search strategy was previously published [15]. An electronic literature search was conducted by two authors (RCFC and VNFQ) through a computerized blinded search of PubMed, EMBASE, Cumulative Index to Nursing and Allied Health Literature (CINAHL), SCOPUS, and Web of Science. The sensitive search strategy is presented in additional file. A literature search was performed from inception to 08/08/2023. An automatic alert system was used to identify studies published during the data extraction process. Additionally, the reference lists of the included studies were hand-searched to identify other relevant studies that might have been missed in the research. No restrictions on language were adopted.

### Data collection process

Two authors (RCFC and VNFQ) screened all retrieved citations independently by reviewing their titles and abstracts. Subsequently, the full-text manuscripts were evaluated for eligibility by the reviewers using a standardized form. The reviewers extracted relevant data from the full-text manuscripts using a data recording form designed for this purpose. Additionally, the risk of bias was assessed using another standardized form. In cases of disagreement, resolution was reached through discussion between the two authors (RCFC and VNFQ). If a disagreement persisted, a third author was consulted for resolution (TDC). Whenever necessary, additional information about a specific study was obtained by directly querying the corresponding authors.

### Risk of bias within studies and across studies

Two authors (RCFC and VNFQ) independently evaluated the quality of each study using the Quality Assessment of Diagnostic Accuracy Studies tool (QUADAS) [16]. Disagreements were resolved through discussion between the two authors (RCFC and VNFQ); however, if a disagreement persisted, a third author (TDC) intervened for resolution. Publication bias was performed with a funnel plot [17]. The funnel plot was constructed using the log diagnostic odds ratio (LnDOR) plotted against 1/effective sample size^1/2^ (EES) [17]. The funnel plot was constructed for each pooled and summarized maneuver.

Investigating publication bias represents a particular challenge in meta-analyses of diagnostic accuracy tests [17]. The diagnostic odds ratio (DOR) of meta-analysis of diagnostic accuracy test is expected to be heterogeneous, and all tests of funnel plot asymmetry have limited power when DOR is heterogeneous [17]. Funnel plots were constructed; however, no statistical assumption was made regarding presence or absence of publication bias [17]. Relying on such statistical assumptions could lead to serious misunderstandings, and thus validity of funnel plot asymmetry becomes questionable [17].

### Definitions of end points

The primary endpoint was to report individual and pooled data regarding the available methods for assessing fluid responsiveness in mechanically ventilated patients. Secondary endpoints were the following: (1) to evaluate diagnostic performance and construct a receiver operating characteristics curve (ROC curve) for the available methods for assessing fluid responsiveness; (2) to aggregate sensitivity and specificity data regarding the methods for assessing fluid responsiveness; (3) to report the frequency of fluid responsiveness patients; (4) to report range and mean threshold values for the methods used to assess fluid responsiveness; (5) to report detailing fluid challenge characteristics such as the type and amount of fluid administered; (6) to report the adopted definition of fluid responsiveness and the device used as gold standard; and (7) to report the baseline hemodynamic parameters, obtained immediately before the fluid challenge, including heart rate (HR), mean arterial pressure (MAP), CO, CI, and central venous pressure (CVP).

### Statistical analysis

The statistical analysis plan has been previously published [15]. Categorical variables are presented as absolute and relative frequencies. Continuous variables are presented as mean ± standard deviation (SD) or median with interquartile range (IQR). The following values for each maneuver were reported: sensitivity, specificity, positive predictive value, negative predictive value, positive likelihood ratio, negative likelihood ratio, accuracy, Youden index, DOR, and area under the receiver operating characteristics curve (AUC). Articles that either did not report these values or did not provide information enabling the calculation of these values were excluded. In cases where these values were not reported, but the article provided information enabling the calculation, these values were calculated using standard formulas outlined in the previously published statistical analysis plan [15]. For the computation of these values, a two-by-two table was constructed, utilizing the counts of true positive, true negative, false positive, and false negative [15].

Individual data for each maneuver used to assess fluid responsiveness were reported. The data from the five most employed maneuvers were aggregated and summarized. A bivariate and hierarchical model incorporating a random effect was constructed to calculate the summary estimates for sensitivity, specificity, and AUC. Sensitivity and specificity for each maneuver were jointly modeled within the study at level one of the analysis [14]. This approach was taken as sensitivity and specificity are connected by shared study characteristics, such as inclusion and exclusion criterion, the definition of fluid responders, and the performance of volume expansion [14]. Forest plot graphs were generated to visualize sensitivity, specificity, and LnDOR along with their respective 95% CI [18]. These plots aimed to identify the presence of outliers and heterogeneity [18]. Heterogeneity was evaluated by Cochran Q statistics; its effect was quantified by using inconsistency (I^2^).

For each maneuver, a summary ROC curve (SROC) was estimated, accompanied by a 95% CI (traditional approach) or 95% credible intervals (Bayesian approach) and a prediction region. Furthermore, for each maneuver, three SROC analyses were conducted using distinct models: the Rutter and Gatsonis hierarchical model; the Moses, Shapiro and Littenberg model, and the Rücker and Schumacher model. Traditional meta-analysis approach [17–21] and Bayesian meta-analysis approach [11, 12] are described in an additional file. All analyses were performed using R 4.2.0 (R Foundation for Statistical Computing, Vienna, Austria).

## Results

### Study selection

The initial search strategy identified a total of 8417 studies. Among these, 69 prospective interventional studies were included in this systematic review and meta-analysis [22–90]. The details of the database search, the process of study selection, and the reasons for study exclusions are demonstrated in figure AF 1.

### Study characteristics

The main characteristics of included studies are presented in Table AF1. In total, data of 3,185 fluid challenges [1589 (49.9%) fluid responders and 1596 (50.1%) fluid non-responders] and 2711 patients were assessed. The Bayesian approach indicated that 50% (48–51%) of the patients were fluid responders.

### Risk of bias within studies and across studies

The QUADAS evaluation for each study is presented in Table AF2. In total, 55 (80%) studies were subjectively classified as high quality. The funnel plot for pulse pressure variation (PPV), stroke volume variation (SVV), plethysmographic variability index (PVI), CVP, and inferior vena cava variation (∆IVC) are shown in figure AF 2 through AF 6. The I^2^ with 95% CI was = 59% (44–70%) for PPV, 59% (36–74%) for SVV, 57% (28–74%) for PVI, 0% (0–58%) for CVP, and 59% (17–80%) for ∆IVC. No evidence of publication bias was found.

## Maneuvers for assessing fluid responsiveness

The five most commonly employed maneuvers to predict fluid responsiveness in mechanically ventilated patients were, respectively, PPV, SVV, PVI, CVP, and ∆IVC. Details of individual performance of these maneuvers is presented in Table 1 and the Bayesian approach is presented in Table AF 3. The main characteristics and individual data of the 205 maneuvers used to assess fluid responsiveness in the included studies are presented in Table AF 4.

**Table 1:** Performance of maneuvers to predict fluid responsiveness in mechanically ventilated patients

Maneuver	N^o^ of studies	N^o^ of patients	N^o^ of fluid challenges (responders/non-responders)	Range threshold	Mean (SD) threshold	Sensitivity (95%CI)	Specificity (95%CI)	DOR (95% CI)	AUC (95% CI)	References
PPV	40	1936	2318 (1118/1200)	4–25.8%	11.5 (3.3) %	74 (70–79)	82 (77–86)	13.61 (9.72–19.05)	0.87 (0.84–0.90)	[22–26, 32, 33, 36, 37, 39–41, 43, 46, 49–51, 53–57, 60, 62–65, 67, 68, 71, 73, 76, 78, 79, 81–84, 88, 90]
SVV	24	1043	1305 (614/691)	8–24.8%	12.1 (3.3) %	76 (71–81)	78 (72–83)	12.23 (7.65–19.58)	0.87 (0.84–0.91)	[22, 23, 26, 35, 36, 38, 39, 41–43, 51, 53–60, 63, 69, 72, 73, 77]
PVI	17	603	671 (382/289)	9.5–20%	13.8 (3.1) %	79 (70–85)	78 (70–84)	12.59 (6.90–22.98)	0.88 (0.82–0.94)	[29, 35, 39, 41, 47–49, 53, 55, 58, 59, 65, 66, 68, 72, 78, 81]
CVP	12	429	429 (264/165)	6.5–12.5 mmHg	9.0 (2.1) mmHg	61 (52–69)	69 (55–81)	3.69 (2.33–5.86)	0.77 (0.69–0.87)	[30, 35, 46, 51, 58, 65, 71, 72, 75, 78, 80, 86]
∆IVC	8	303	303 (152/151)	11.1–21%	15.4 (3.5) %	66 (54–75)	81 (70–88)	9.03 (4.26–19.13)	0.83 (0.78–0.89)	[25, 30, 35, 45, 69, 70, 86, 87]

The reported threshold value point represents the mean value of the maneuver. Sensitivity, specificity, DOR, and AUC are reported along with their respective 95% confidence interval. Sensitivity and specificity are expressed as percentages

AUC, area under the receiver operating characteristics curve; CVP, central venous pressure; DOR, diagnostic odds ratio; ∆IVC, inferior vena cava variation; N^o^, number; PPV, pulse pressure variation; PVI, plethysmographic variability index; SSV, stroke volume variation

**Fig. 1:**
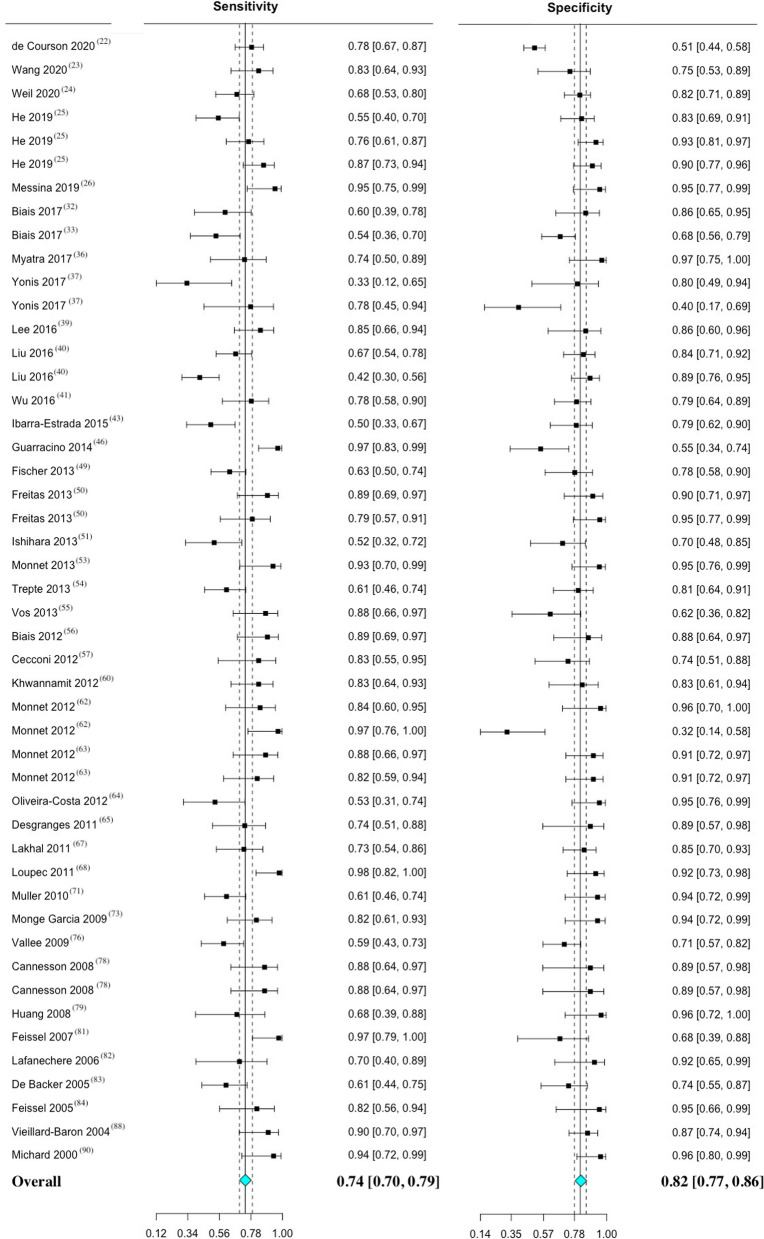
Paired forest plot of sensitivity and specificity with 95% CI of pulse pressure variation—PPV. The overall result represents a random effect model. Inconsistency (I^2^) with 95% CI = 59% (44–70%)

**Fig. 2:**
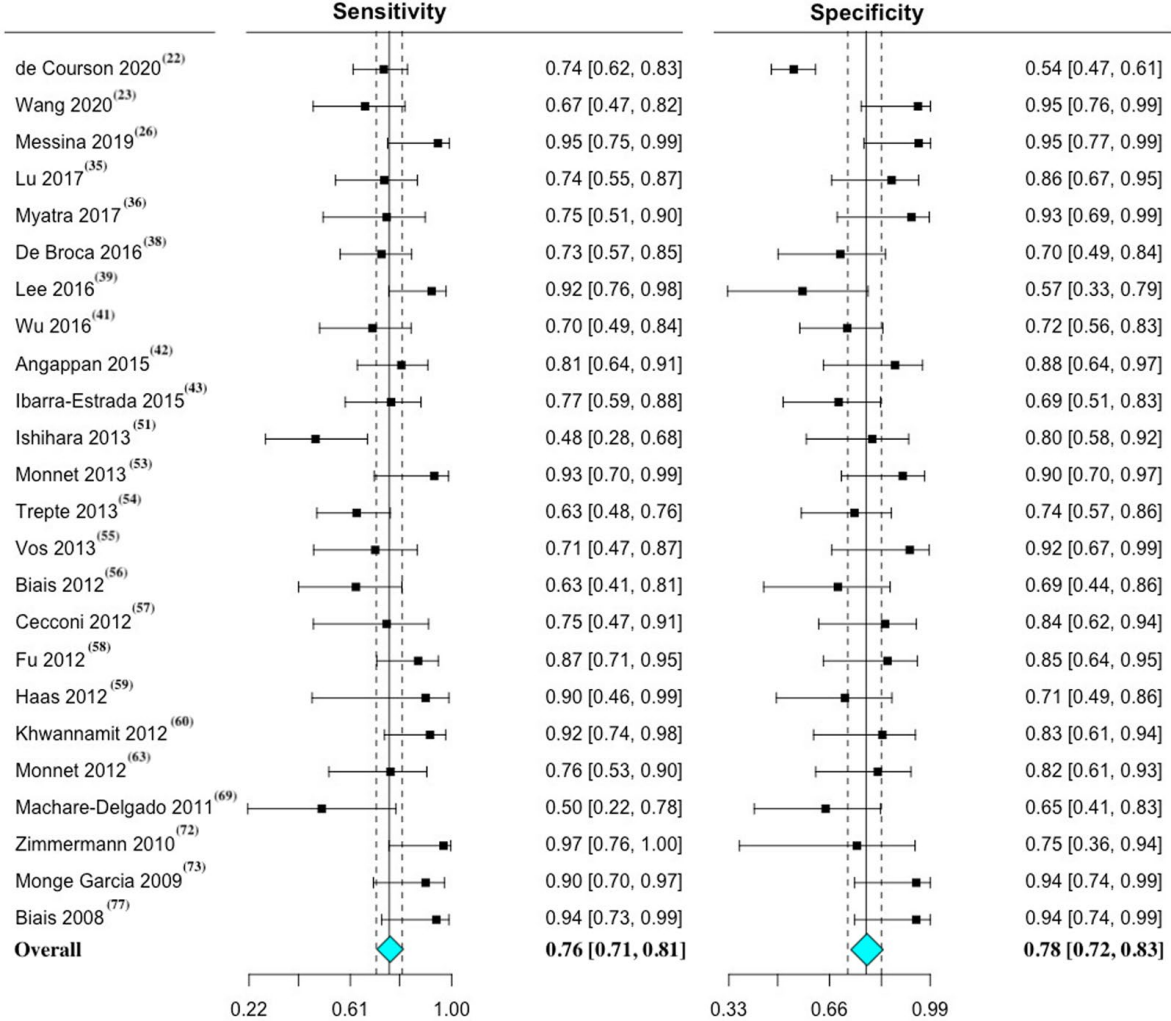
Paired forest plot of sensitivity and specificity with 95% CI of stroke volume variation—SVV. The overall result represents a random effect model. Inconsistency (I^2^) with 95% CI = 59% (36–74%)

**Fig. 3:**
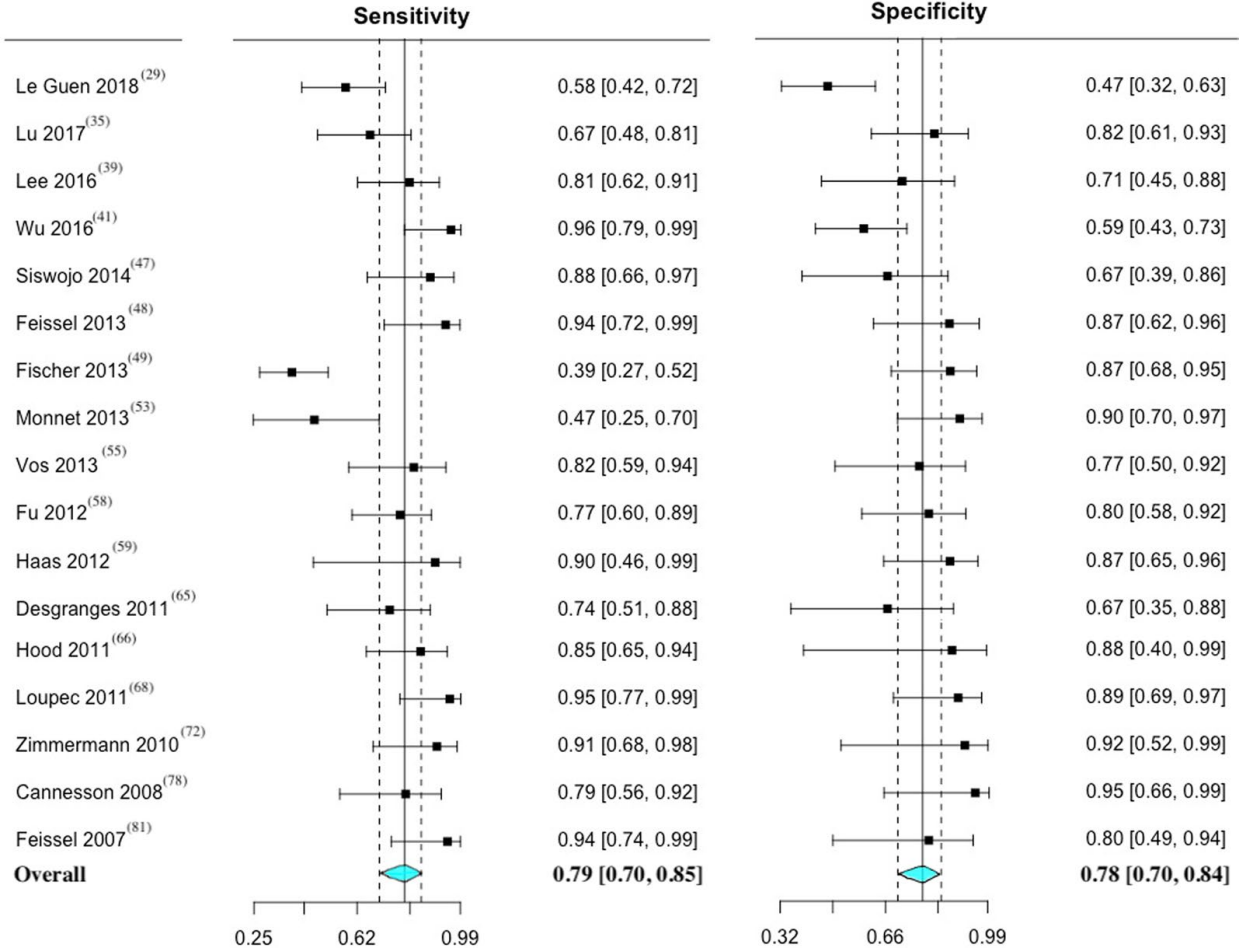
Paired forest plot of sensitivity and specificity with 95% CI of plethysmographic variability index—PVI. The overall result represents a random effect model. Inconsistency (I^2^) with 95% CI = 57% (28–74%)

The paired forest plots of sensitivity and specificity with 95% CI was performed for PPV (Fig. 1), SVV (Fig. 2), PVI (Fig. 3), CVP (figure AF 7), and ∆IVC (figure AF 8). The forest plot of LnDOR with 95% CI is presented in additional file for PPV, SVV, PVI, CVP, and ∆IVC (figure AF 9 through 13).

**Fig. 4:**
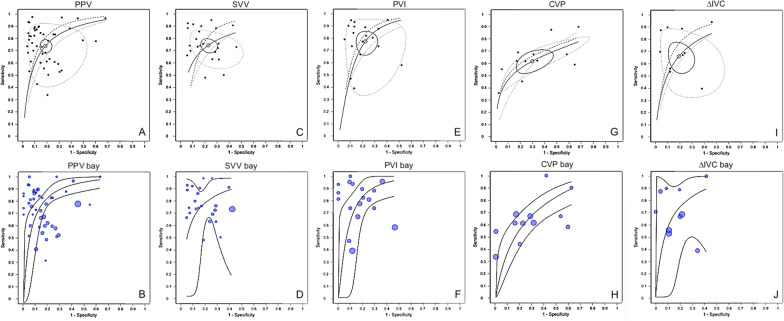
Summary ROC curve (SROC) with prediction region, and Bayesian SROC with prediction region. Panel A: SROC of pulse pressure variation (PPV). Panel B: Bayesian SROC of PPV. Panel C: SROC of stroke volume variation (SVV). Panel D: Bayesian SROC of SVV. Panel E: SROC of plethysmographic variability index (PVI). Panel F: Bayesian SROC of PVI Panel G: SROC of central venous pressure (CVP). Panel H: Bayesian SROC of CVP. Panel I: SROC of inferior vena cava variation (∆IVC). Panel J: Bayesian SROC of ∆IVC

The SROC with 95% confidence and Bayesian SROC with 95% credible levels along with their respective prediction region for PPV, SVV, PVI, CVP, and ∆IVC are presented in Fig. 4. Bayesian SROC with posterior predictive contour is presented in additional file for PPV, SVV, PVI, CVP, and ∆IVC (figure AF 14 through 18).

### Comparison PPV versus SVV

A total of 15 studies [15 of 69 (21.7%)], encompassing 539 patients and 801 fluid challenges (352 responders; and 449 non-responders) simultaneously applied PPV and SVV to assess fluid responsiveness. Out of these, 8 (53.3%) studies [23, 41, 51, 53, 56, 57, 63, 73] reported a higher AUC value for PPV, 2 (13.3%) studies [43, 55] reported a higher AUC value for SVV, and 5 (33.3%) studies [22, 36, 39, 54, 60] reported that the AUC were nearly equal (with a difference ≤ 2%). In these 15 studies simultaneously applying PPV and SVV to assess fluid responsiveness, the AUC (95% CI) values for PPV and SVV were, respectively, 0.86 (0.81–0.92) and 0.86 (0.81–0.91).

### Comparison CVP versus PPV, SVV, PVI, and ∆IVC

A total of 10 studies [10 of 69 (14.5%)] [30, 35, 46, 51, 58, 65, 71, 72, 78, 86] simultaneously applied CVP and PPV or SVV or ∆IVC or PVI to assess fluid responsiveness. Of these 10 studies, only 1 study [51] reported a higher AUC value for CVP. Notably, 21 studies adopting CVP as a maneuver to predict fluid responsiveness opted not to report the sensitivity and specificity values due to their lower accuracy compared to other maneuvers in those studies. Therefore, data regarding the use of CVP as a maneuver to predict fluid responsiveness from those studies could not be included in the meta-analyses [23, 28, 36, 37, 40–43, 45, 49, 55, 57, 60, 64, 70, 73, 76, 79, 83, 88, 90].

### Fluid challenge characteristics

Colloid solutions remain the most frequently used I.V. fluid employed for performing fluid challenge compared to crystalloids solution (Table AF 5). However, over the years there has been a substantial decline in the number of studies using colloid solutions [hydroxyethyl starch (HES)] and a significant increase in the utilization of crystalloid solutions (saline solutions) (figure AF 19 and figure AF 20).

The amount of fluid infused for conducting a fluid challenge exhibits considerable variability (Table AF 5). The infused volume ranges from 200 ml [35] to 1000 ml [64, 83] or alternatively from 4 ml/kg [59] to 15 ml/kg [55]. The amount of I.V. fluid most frequently administered/infused for conducting a fluid challenge was 500 ml (Table AF 5). Among the studies that infused 500 ml for a fluid challenge, 15 studies [15 of 69 (21.7%)] used saline solution and 12 studies [12 of 69 (17.4%)] used HES.

### Definitions and devices adopted to define fluid responsiveness

The most frequently [29 of 69 (42.0%) studies] adopted definition for fluid responsiveness was an increase in CI ≥ 15% (Table AF 5). The most frequently used device to determine CO/CI was pulse indicator continuous cardiac output (PiCCO) [22 of 69 (31.9%) studies] (Table AF 5).

### Hemodynamic variables

Baseline value of HR, MAP, CVP, CO and CI and the HR, MAP, and CVP variation induced by fluid challenge did not allow the categorization of patients as fluid responders or fluid non-responders (additional file Table AF 6).

## Discussion

The main finding of this systematic review and meta-analysis suggests that fluid responsiveness can be reliably assessed in adult patients under mechanical ventilation. Our findings indicate that when fluid responsiveness is assessed, approximately half of the patients will respond to a fluid administration. Furthermore, we demonstrated that PPV, SVV, and PVI proved to be the best maneuvers, while ∆IVC and CVP are intermediate, and systemic hemodynamic parameters such as MAP and HR are poor in predicting which patients would benefit from volume expansion. Since fluid overload has been associated with increased morbidity and mortality, our findings have significant clinical implications and reinforce the importance of a proper evaluation of fluid responsiveness in critically ill patients [4].

An understanding of the application and limitations of each available maneuver to assess fluid responsiveness is crucial for obtaining accurate information. Among the various maneuvers studied for predicting fluid responsiveness, PPV and SVV stand out as the most explored. One of the advantages of PPV and SVV is their continuous monitoring capability, which is associated with minimal interrater variability. However, it is important to note that these maneuvers should not be interpreted in isolation. Ventilatory settings play a significant role as the variations depend on cardiovascular and respiratory mechanisms. On the respiratory side, these mechanisms include factors such as tidal volume, lung volume, PEEP, pleural pressure, and chest wall and lung compliances [25, 36, 37, 40, 50, 62]. It is worth mentioning that the predictive value of PPV and SVV is limited in patients mechanically ventilated with low tidal volume, high PEEP levels, and low compliance of the respiratory system [25, 36, 37, 40, 50, 62]. In this current systematic review and meta-analysis, there were no instances where the study protocol employed a tidal volume lower than 5 ml/kg or involved spontaneous modes of mechanical ventilation or respiratory movements.

PVI is a non-invasive method that enables continuous assessment of fluid responsiveness with minimal interrater variability. However, it is worth noting that critically ill patients often display signs of low perfusion, which can reduce the reliability of the PI signal [65]. The accuracy of PVI is significantly influenced by the adequacy of perfusion. Other variables, such as abnormal peripheral perfusion, use of vasopressor, hypothermia, and low CO could impact the accuracy of PVI [39]. Both PI and PVI can be measured at the finger, ear, and forehead [65].

∆IVC is an echocardiographic maneuver that can be used to assess a patient’s fluid responsiveness without an invasive arterial line [86, 87]. Although it relies on the operator's skill, echocardiography is a non-invasive technique that can be learned fast. Echocardiography is routinely used in the ICU and allows for intermittent measurements of ∆IVC, as well as stroke volume and CO [86, 87]. For patients who do not require continuous CO monitoring, echocardiography could be an interesting alternative for monitoring changes in stroke volume, CO, and heart function. There are two standardized methods for calculating ΔIVC, and both are equally accepted [86, 87].

CVP is an intermediate maneuver for predicting which patients may benefit from volume expansion. When compared to PPV or SVV or ∆IVC or PVI, only one study [51] reported a higher AUC value for CVP. Consequently, caution should be exercised when using CVP to guide volume expansion. The baseline CVP value did not allow the classification of patients as fluid responders or fluid non-responders in 83% of the studies, and the variation in CVP induced by a fluid challenge did not allow the classification of patients as fluid responders or fluid non-responders in 92% of the studies. Importantly, as 21 studies mentioning the poor predictive value of CVP did not report sensitivity and specificity values, the aggregated values we reported may be too optimistic.

For the management of hemodynamically unstable patients, numerous variables, aside from assessing intravascular volume and identifying patients who will benefit from an intravenous infusion of fluids, can influence patient outcomes [91–97]. It is also important to note that the cut-offs presented in most trials (and aggregated in this metanalysis) represent the best compromise between sensitivity and specificity. According to patients’ conditions, it may be interesting to select lower or higher cut-offs, optimized for specificity in patients expected to be of limited tolerance to fluids (such as severe ARDS) or optimized sensitivity in patients with high benefit/risk profiles (such as septic shock with severely impaired tissue hypoperfusion but minimal respiratory dysfunction). Only one trial provided such optimized thresholds [98]; therefore, it was not feasible to evaluate the impact of selecting lower or higher cut-offs according to patients’ conditions.

Previous meta-analyses have assessed maneuvers to assess fluid responsiveness in various clinical scenarios, demonstrating their overall good performance [9, 99]. Our meta-analysis confirms these findings and to address common issues in meta-analysis concerning fluid responsiveness, this meta-analysis performed a traditional and a Bayesian approach. The Bayesian approach offers flexibility and can accommodate complex likelihood functions other than normal distribution [20, 21]. Furthermore, the Bayesian approach is expected to provide more robust credible intervals even with a limited number of studies [20, 21]. The study included a wide range of patients in various clinical settings, different reference tests, diverse volume expansion approaches, and varying reporting methods for validating the index test. While this diversity might reduce the power of pooled data, it also has the potential to guide bedside decision-making by allowing the selection of appropriate and available devices. This diversity increases the applicability of the study findings. Thus, what might be seen as a limitation was converted into a strength of the study, as it enabled the inclusion of a broad range of maneuvers and the consideration of results from individual studies.

This study has limitations. It is important to emphasize that the results of this systematic review and meta-analysis should be interpreted in the context of the included studies. These studies varied significantly, with clinical scenarios, methodology, and sample size differences. Some studies had relatively small sample sizes, and this might introduce a small study effect. To address this issue, a Bayesian approach was used. Additionally, systematic reviews are susceptible to publication bias, which can potentially exaggerate study conclusions if publication is related to the strength of the results. Furthermore, there is a limitation related to transforming continuous diagnostic indices, such as PPV, SVV, PVI, ∆IVC, and CVP into binary variables (i.e., responders or non-responders). This represents an inherent limitation of all methods for assessing fluid responsiveness. In this analysis, it’s not feasible to take into account the “grey-zone” concept, which would have made possible to limit this dichotomic aspect. Additionally, different cut-offs are often used. While it may be necessary to use some techniques to achieve higher values due to the elevated least significant change with the specific device, by using other tools, lower values may also be valid. In this systematic review it was not possible to alter the cut-off selected in the primary studies a posteriori. Additionally, the objective of the study was to describe maneuvers for assessing fluid responsiveness in mechanically ventilated patients, and data from the five most employed maneuvers were aggregated. As consequence, the conclusions should not be interpreted as identifying the best maneuvers, as the study did not compare the aggregated maneuvers with well-established and reliable tests, such as the passive leg raising test, end-expiratory occlusion test, and tidal volume challenge. The GRADE system (Grading of Recommendations, Assessment, Development, and Evaluations) was not used to assess the quality of the meta-analysis since it was not foreseen in the study protocol. Finally, the cut-off as selected by the Youden index represents the best compromise between sensitivity and specificity. In some situations, it may be preferable to optimize sensitivity (low risk of fluid overload profile), while in others, optimizing specificity may be desirable (as in ARDS) [98].

## Conclusion

Among the five maneuvers compared in predicting fluid responsiveness, PPV, SVV, and PVI were superior to CVP and ∆IVC. However, there is no data supporting any of the above mentioned as being the best maneuver. Furthermore, it has been demonstrated that values of mean arterial pressure, heart rate, and central venous pressure before volume expansion, and their variations induced by volume expansion were not associated with changes in cardiac output. Consequently, these variables should not be used to guide volume expansion.

### Supplementary Information

Additional file 1

## Data Availability

The datasets generated during and/or analyzed during the current study are available from the corresponding author on reasonable request.

## Abbreviations

95% CI: 95% Confidence interval
95% CrI: 95% Credible interval
AUC: Area under the receiver operating characteristics curve
CI: Cardiac index
CO: Cardiac output
CVP: Central venous pressure
DOR: Diagnostic odds ratio
EES: Effective sample size
I^2^: Inconsistency
ICU: Intensive care unit
I.V.: Intravenous
∆IVC: Inferior vena cava variation
LnDOR: Log diagnostic odds ratio
PiCCO: Pulse indicator continuous cardiac output
PPV: Pulse pressure variation
PVI: Plethysmographic variability index
ROC curve: Receiver operating characteristics curve
SD: Standard deviation
SROC: Summary ROC curve
SVV: Stroke volume variation
VTI: Velocity-time integral
