# Supplementary material for: Assessment of fluid responsiveness using pulse pressure variation, stroke volume variation, plethysmographic variability index, central venous pressure, and inferior vena cava variation in patients undergoing mechanical ventilation: a systematic review and meta-analysis
Source: Crit Care. 2024 Aug 31;28:289. doi: 10.1186/s13054-024-05078-9 (PMC11366151; doi:10.1186/s13054-024-05078-9)
Supplement: Supplementary file 1 — Additional file 1 [file 13054_2024_5078_MOESM1_ESM.docx]

**Assessment of fluid responsiveness using pulse pressure variation, stroke volume variation, plethysmographic variability index, central venous pressure, and inferior vena cava variation in patients undergoing mechanical ventilation: a systematic review and meta-analysis**

**Additional file**

**Authors:** Renato Carneiro de Freitas Chaves, Carmen Silvia Valente Barbas, Veronica Neves Fialho Queiroz, Ary Serpa Neto, Rodrigo Octavio Deliberato, Adriano José Pereira, Karina Tavares Timenetsky, João Manoel Silva Júnior, Flávio Takaoka, Daniel de Backer, Leo Anthony Celi, Thiago Domingos Corrêa.

**ADDITIONAL FILE APPENDIX**

**ADDITIONAL METHODS**

**ADDITIONAL RESULTS**

**Figure AF 1.** Flow diagram of database search and study selection.

**Figure AF 2.** Funnel plot of pulse pressure variation - PPV.

**Figure AF 3.** Funnel plot of stroke volume variation - SVV.

**Figure AF 4.** Funnel plot of plethysmographic variability index - PVI.

**Figure AF 5.** Funnel plot of central venous pressure - CVP.

**Figure AF 6.** Funnel plot of inferior vena cava variation - ∆IVC.

**Figure AF 7.** Paired forest plot of sensitivity and specificity with 95% CI of central venous pressure - CVP.

**Figure AF 8.** Paired forest plot of sensitivity and specificity with 95% CI of inferior vena cava variation - ∆IVC.

**Figure AF 9.** Forest plot of LnDOR with 95% CI of pulse pressure variation - PPV.

**Figure AF 10.** Forest plot of LnDOR with 95% CI of stroke volume variation - SVV.

**Figure AF 11.** Forest plot of LnDOR with 95% CI of plethysmographic variability index - PVI.

**Figure AF 12.** Forest plot of LnDOR with 95% CI of central venous pressure - CVP.

**Figure AF 13.** Forest plot of LnDOR with 95% CI of inferior vena cava variation - ∆IVC.

**Figure AF 14.** Bayesian SROC with posterior predictive contour of pulse pressure variation - PPV.

**Figure AF 15.** Bayesian SROC with posterior predictive contour of stroke volume variation - SVV.

**Figure AF 16.** Bayesian SROC with posterior predictive contour of plethysmographic variability index - PVI.

**Figure AF 17.** Bayesian SROC with posterior predictive contour of central venous pressure - CVP.

**Figure AF 18.** Bayesian SROC with posterior predictive contour of inferior vena cava variation - ∆IVC.

**Figure AF 19.** The use of hydroxyethyl starch (HES) solution and saline solution by year.

**Figure AF 20.** The use of colloid solution and crystalloid solution by year.

**Table AF 1.** Individual characteristics of included studies.

**Table AF 2.** The Quality Assessment of Diagnostic Accuracy Studies tool (QUADAS) of each individual study.

**Table AF 3**. Bayesian approach to summary the performance of maneuvers to predict fluid responsiveness in mechanically ventilated patients.

**Table AF 4.** Individual data of each maneuver to assess fluid responsiveness in mechanically ventilated patients.

**Table AF 5.** Fluid challenge characteristics, definition of fluid responsiveness and devices adopted to define fluid responsiveness.

**Table AF 6.** The baseline value and the variation induced by the fluid challenge of heart rate, mean arterial pressure, cardiac output, cardiac index, and central venous pressure.

**ADDITIONAL METHODS**

**Information sources and search**

The following sensitive search strategy was applied, combining the following Medical Subject Headings and keywords: (“operating room” or “intensive care unit” or “intensive care” or “ICU” or “critical ill” or “critical care” or “intensive care” or “emergency department”) and (“mechanical ventilation” or “artificial respiration” or “positive-pressure ventilation” or “continuous positive airway pressure”) and (“hemodynamics” or “pulse contour” or “systolic pressure variation” or “systolic pressure volume” or “pulse pressure” or “stroke volume” or “cardiac output” or “passive leg raising” or “ultrasonography” or “echocardiographic” or “preload”) and (“fluid” or “fluid challenge” or “fluid therapy” or “fluid responsiveness” or “volume” or “volume responsiveness”).

**Traditional meta-analysis approach**

A bivariate model was utilized to calculate the summary estimates of both sensitivity and specificity, expressed with the respectively 95% confidence interval (95%CI). Additionally, a random effect model was utilized to calculate pooled data, aiming to mitigate disparities within the study population. To determine the overall accuracy, DerSimonian-Laird random-effect model was utilized to calculate the diagnostic odds ratio, while the Holling’s proportional hazard models were used to calculate the area under the receiver operating characteristics curve (AUC). The analysis was supported using the following R packages: “reitsma {mada}” for sensibility and specificity; “madauni {mada}” for DOR; “phm {mada}” for AUC.

**Bayesian meta-analysis approach**

A Bayesian bivariate model incorporating a random effect was constructed to calculate the summary estimates for both sensitivity and specificity. For the construction of this Bayesian model, a Logit link function was applied. Estimations for sensitivity and specificity, along with the corresponding 95% credible intervals (CrI), were performed for each maneuver. With the aim of mitigating any potential influences on the model's outcomes, a noninformative prior was used to calculate the posterior distribution.

The Bayesian approach was a Markov Chain Monte Carlo simulation, which encompassed the utilization of five Markov chains, 50,000 interactions and the initial 10,000 interactions were discarded. For each maneuver, the estimation of Bayesian AUC and Bayesian SROC was carried out, and the resulting estimates were presented along with the associated 95% CrI. A beta distribution was employed to calculate the proportion of fluid responsiveness patients. This beta prior distribution, for the proportion of fluid responsiveness patients, integrated information from previously published studies, which indicated that half of the patients were fluid responsiveness.

The determination of the 95% CrI for the posterior probabilities relating to the proportion of fluid responsiveness patients were accomplished by calculating the 2.5th and 97.5th percentiles of the posterior distribution. The analysis was supported by the use of the following R packages: “beta.select {LearnBayes}” for constructing the beta distribution of proportion; “metadiag {bamdit}” for sensibility and specificity, “metadiag {bamdit}” and “bsroc {bamdit}” for AUC and SROC estimation.

**ADDITIONAL RESULTS**

**Pulse pressure variation - PPV**

Pulse pressure variation (PPV) was calculated as the difference in pulse pressure maximal (PPmax) and pulse pressure minimal (PPmin) divided by the mean between PPmax and PPmin, as follow: PPV (%) = (PPmax − PPmin) / [(PPmax + PPmin) / 2] X 100. ^(69)^

Stroke volume variation (SVV) was mostly calculated as the difference in stroke volume maximal (SVmax) and stroke volume minimal (SVmin) divided by the mean between SVmax and SVmin, as follows: SVV (%) = (SVmax − SVmin) / [(SVmax + SVmin) / 2] X 100. ^(56)^ SVV could also be calculated as the area under the systolic part of the arterial pressure waveform (Asys) divided by the systemic vascular impedance (Ztot) as follows: SV = (Asys / Ztot). ^(5)^

Plethysmographic variability index (PVI) is based on the perfusion index (PI) that is an indicator of the PVI signal and represents the ratio between pulsatile direct current (DC) and non-pulsatile alternating current (AC). ^(57)^ PI was calculated as follows: PI (%) = (AC/DC) X 100. ^(57)^ PVI was calculated as the difference in PVI maximal (PPmax) and PVI minimal (PPmin) divided by PVImax, as follows: PVI (%) = [(PImax - PImin) / PImax] X 100. ^(57)^

Inferior vena cava variation (∆IVC) is based on the inferior vena cava (IVC) measurements. IVC could be measured by transthoracic echocardiography with subxyphoidal long axys or subcostal view in the longitudinal section. ^(24, 65, 66)^ The IVC diameter was measured using the M-mode strictly perpendicular to the vessel, above the juncture with the hepatic vein, usually no more than 2-3 centimeters away from the right atrium. ^(24, 65, 66)^ ∆IVC could be calculated by two standard formulas. ^(24, 65, 66)^ ∆IVC could be calculated as the difference in the IVC maximum diameter (IVCmax) and IVC minimum diameter (IVCmin) divided by IVCmin, as follows: ∆IVC (%) = (IVCmax - IVCmin) / (IVCmin) X 100. ^(65)^ ∆IVC could be also calculated as: ∆IVC (%) = (IVCmax - IVCmin) / [(IVCmax + IVCmin) / 2] X 100. ^(66)^

**Figure AF 1.** Flow diagram of database search and study selection.


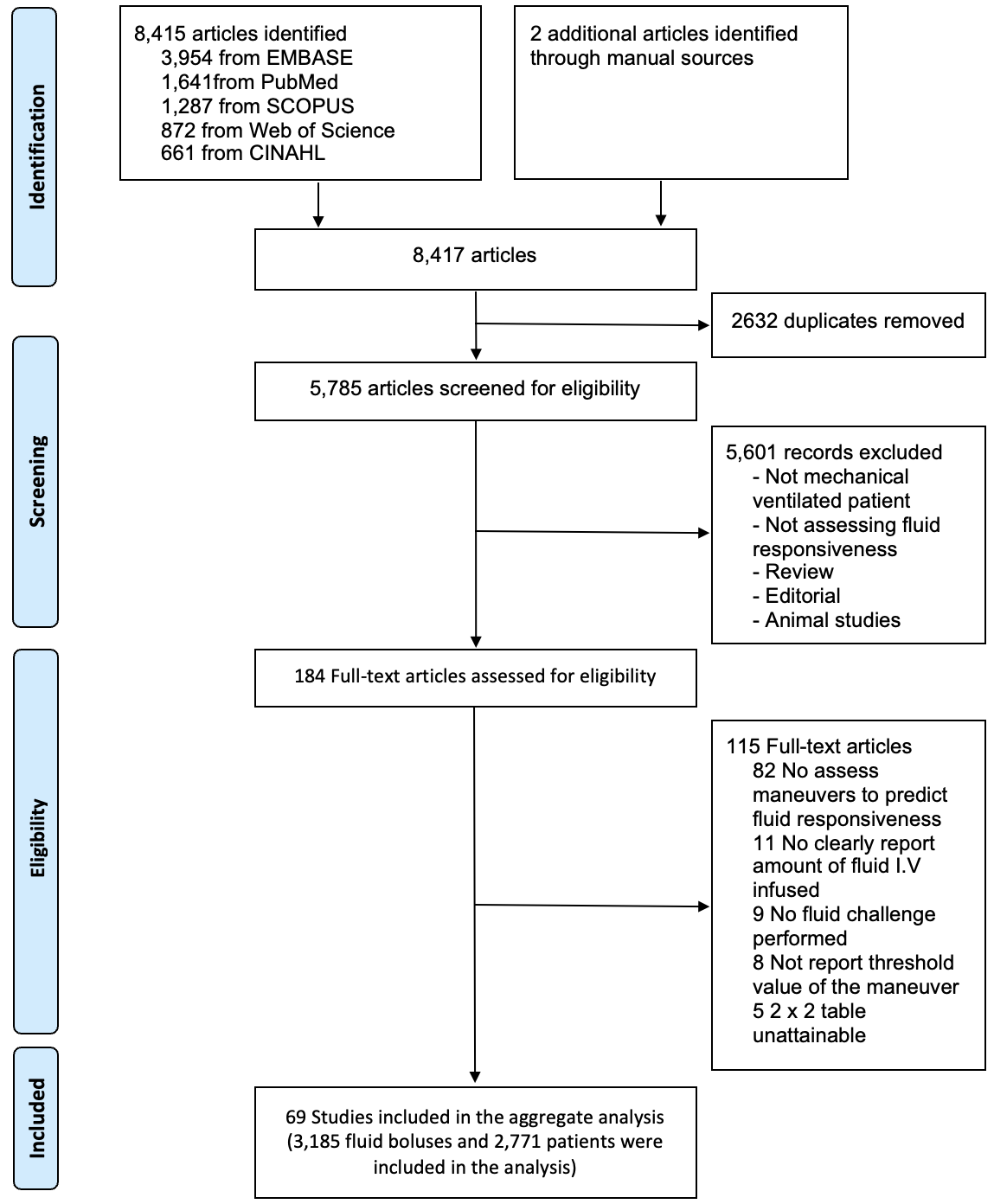


**Figure AF 2.** Funnel plot of pulse pressure variation - PPV.


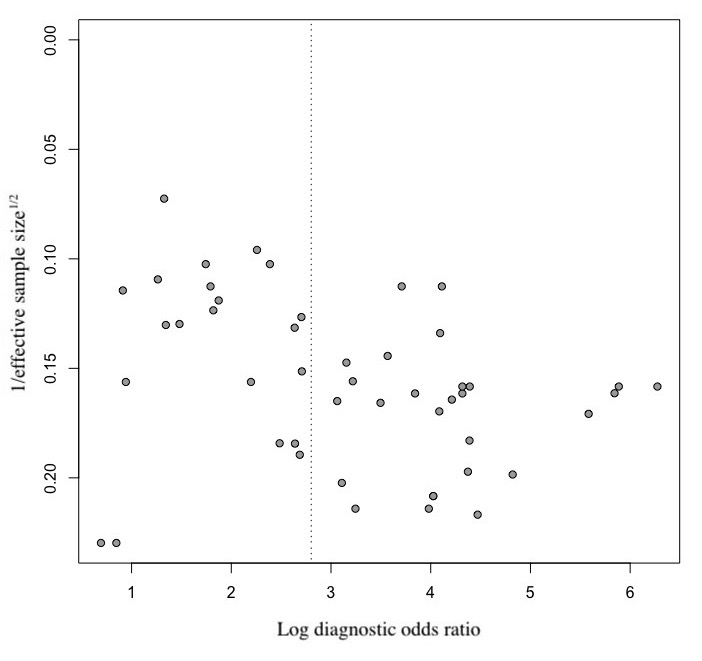


Note. Vertical line was plotted using random effects of log diagnostic odds ratio.

**Figure AF 3.** Funnel plot of stroke volume variation - SVV.


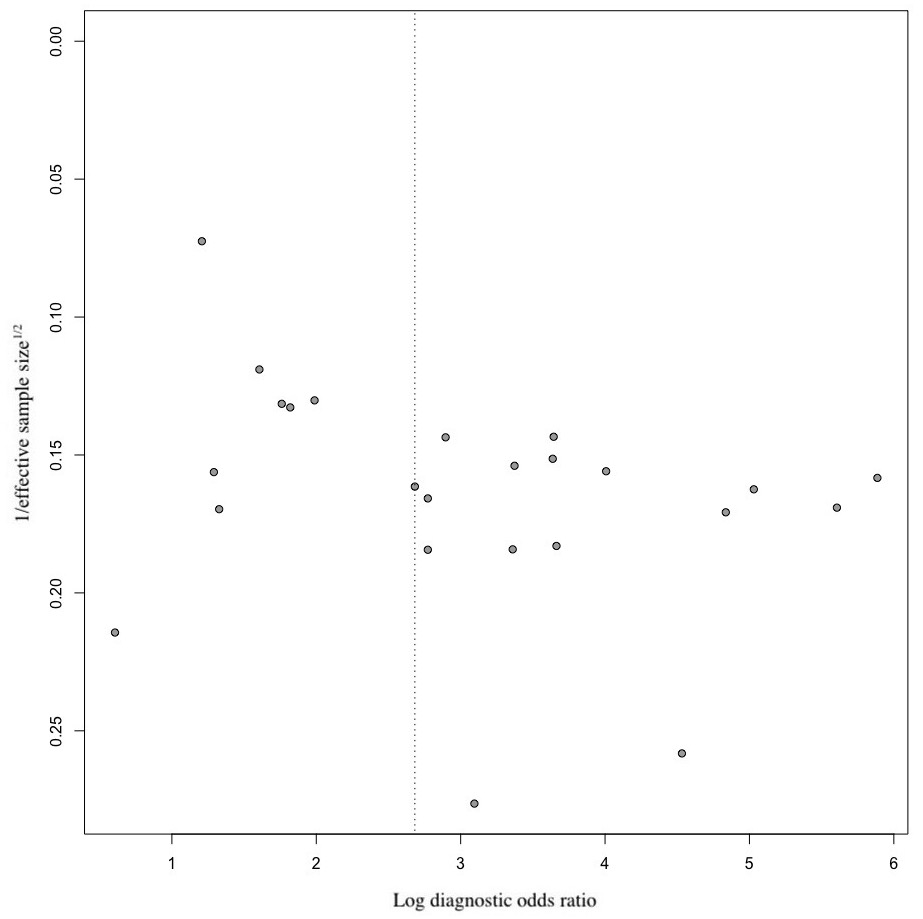


Note. Vertical line was plotted using random effects of log diagnostic odds ratio.

**Figure AF 4**. Funnel plot of plethysmographic variability index - PVI.


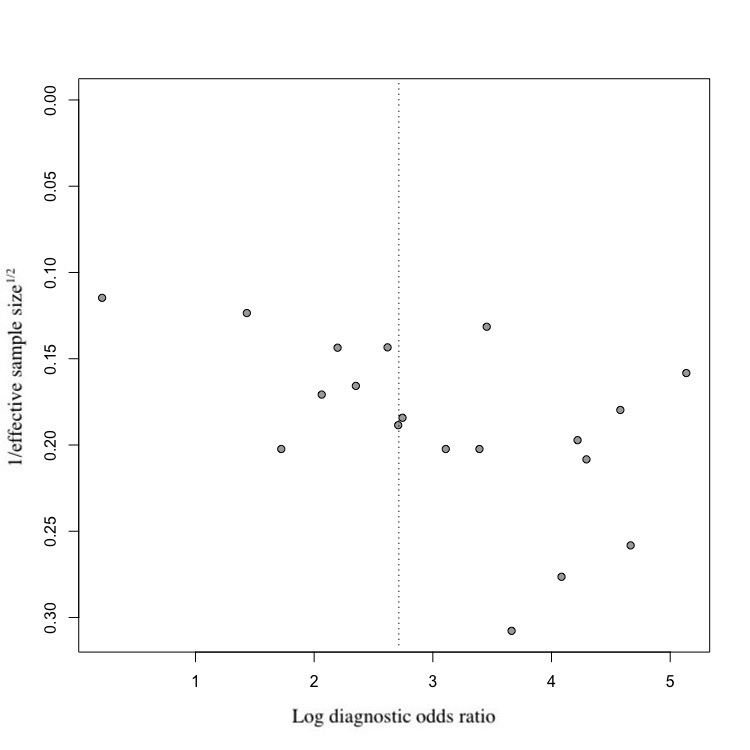


Note. Vertical line was plotted using random effects of log diagnostic odds ratio.

**Figure AF 5**. Funnel plot of central venous pressure - CVP.


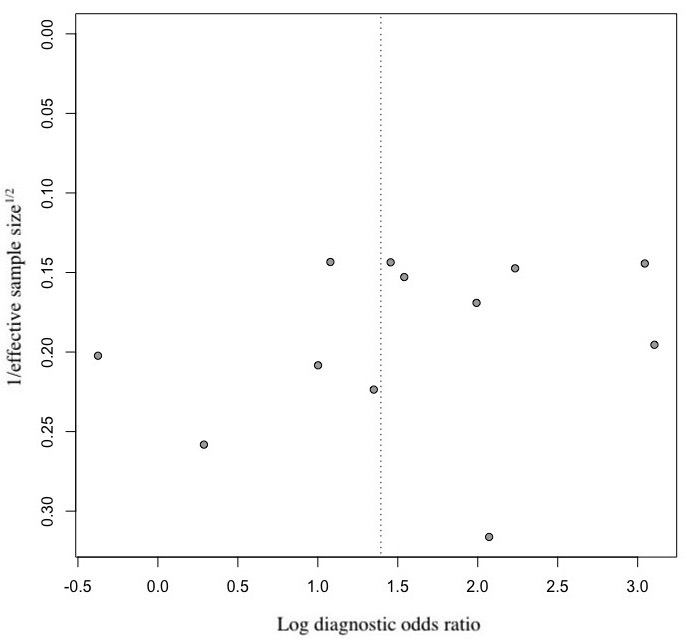


Note. Vertical line was plotted using random effects of log diagnostic odds ratio.

**Figure AF 6.** Funnel plot of inferior vena cava variation - ∆IVC.


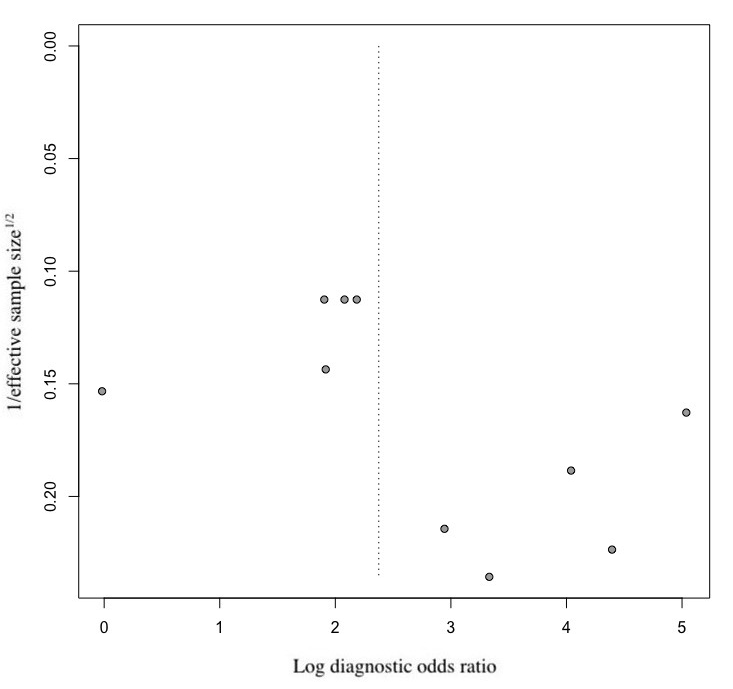


Note. Vertical line was plotted using random effects of log diagnostic odds ratio.

**Figure AF 7.** Paired forest plot of sensitivity and specificity with 95% CI of central venous pressure - CVP.

**
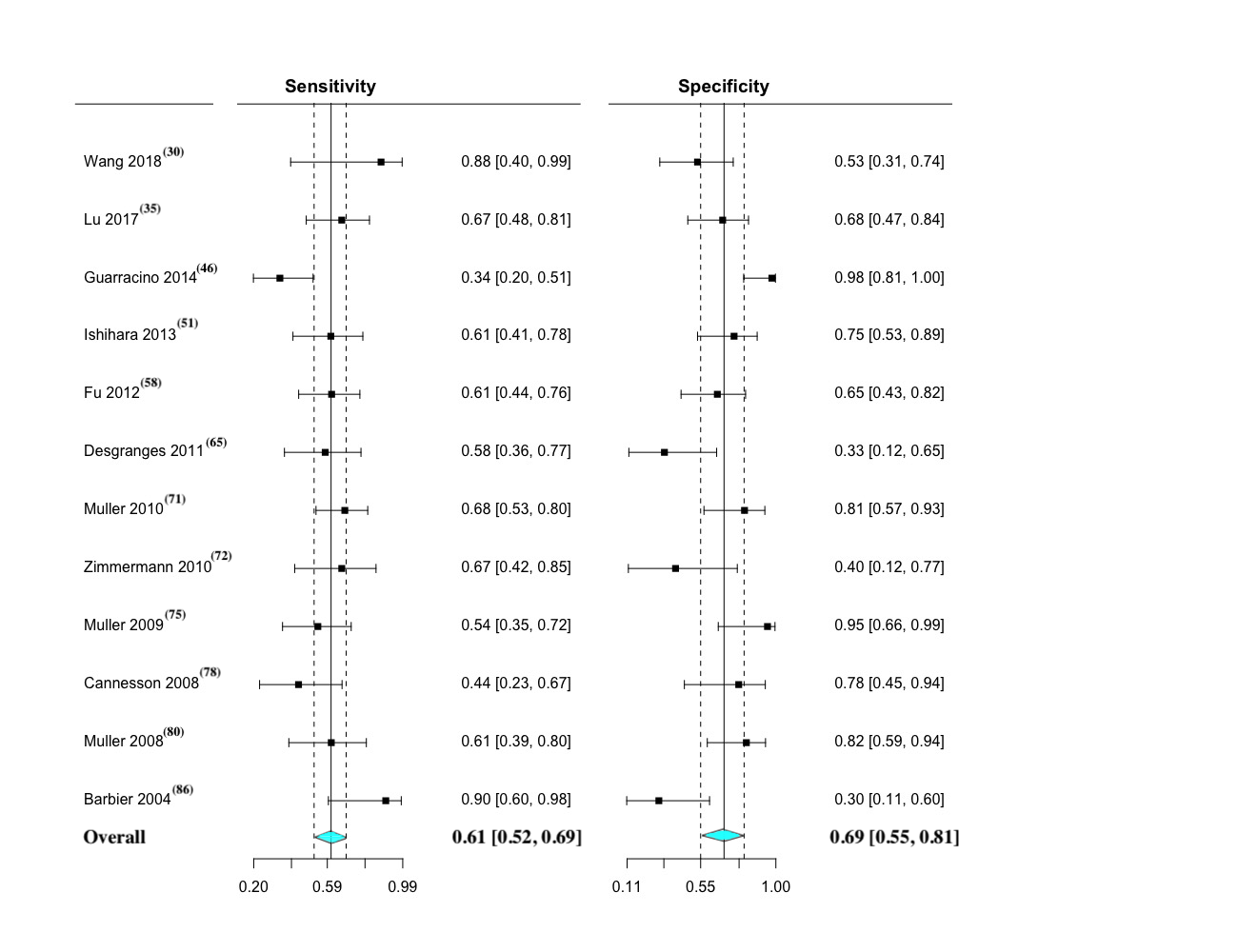
**

**Legend.** The overall result represents a random effect model. Inconsistency (I^2^) with 95% CI = 0% (0%-58%).

**Figure AF 8.** Paired forest plot of sensitivity and specificity with 95% CI of inferior vena cava variation - ∆IVC.

**
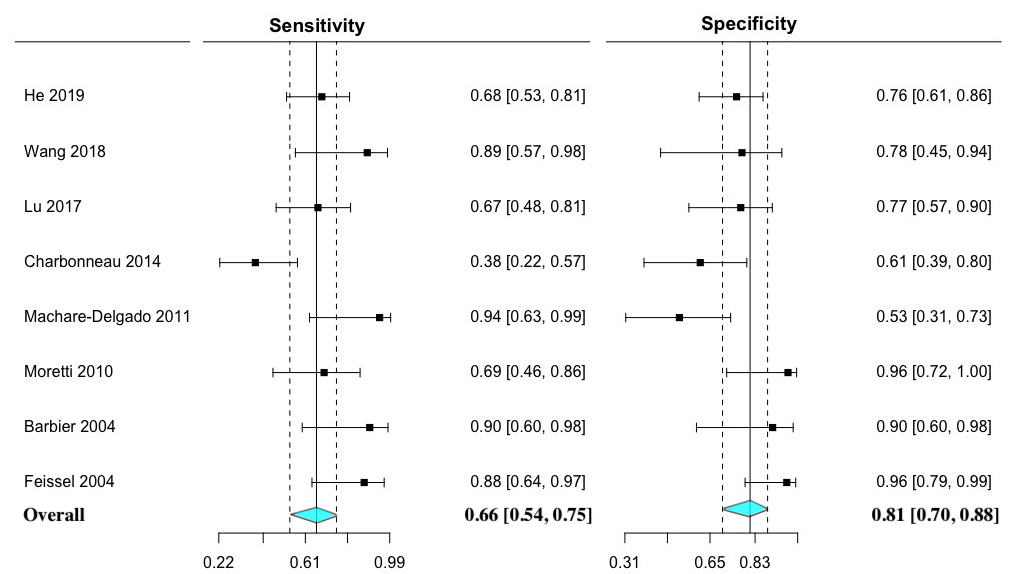
**

**Legend.** The overall result represents a random effect model. Inconsistency (I^2^) with 95% CI = 59% (17%-80%).

**Figure AF 9.** Forest plot of LnDOR with 95% CI of pulse pressure variation – PPV.


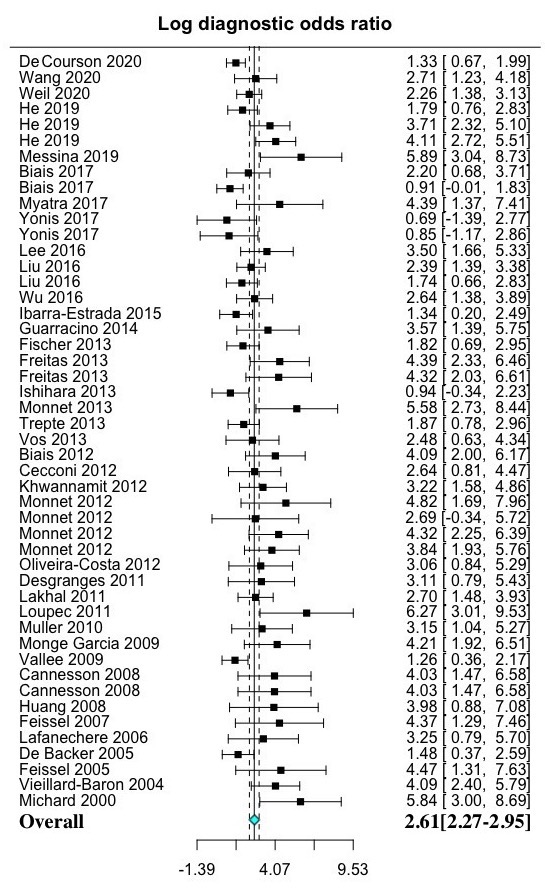


**Figure AF 10.** Forest plot of LnDOR with 95% CI of stroke volume variation - SVV.


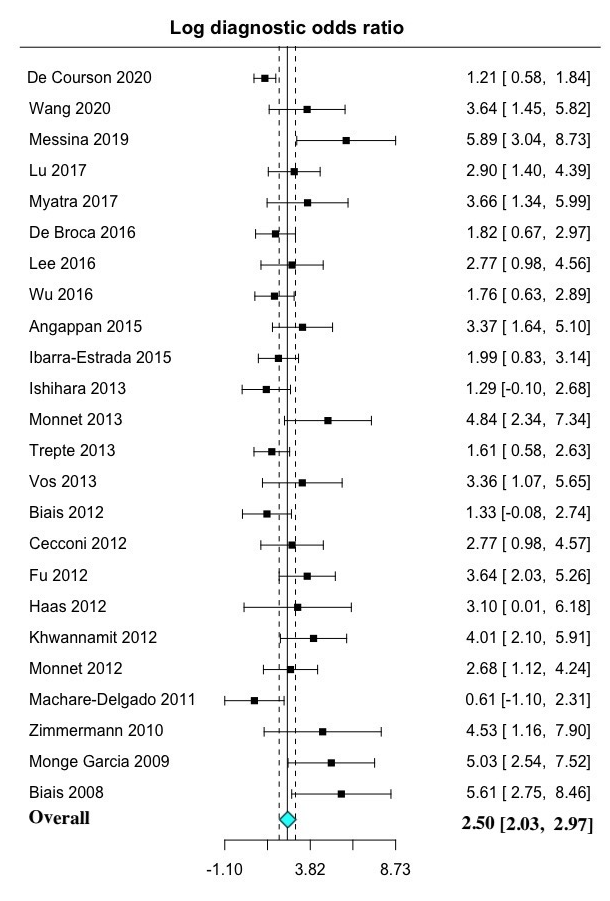


**Figure AF 11.** Forest plot of LnDOR with 95% CI of plethysmographic variability index - PVI.


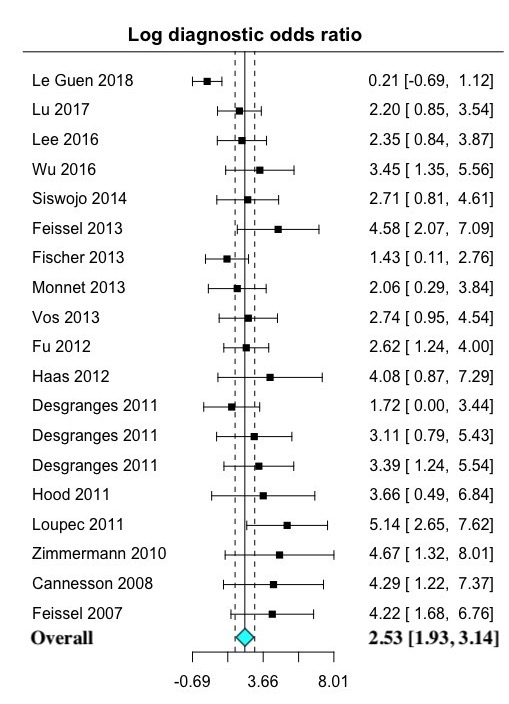


**Figure AF 12.** Forest plot of LnDOR with 95% CI of central venous pressure - CVP.


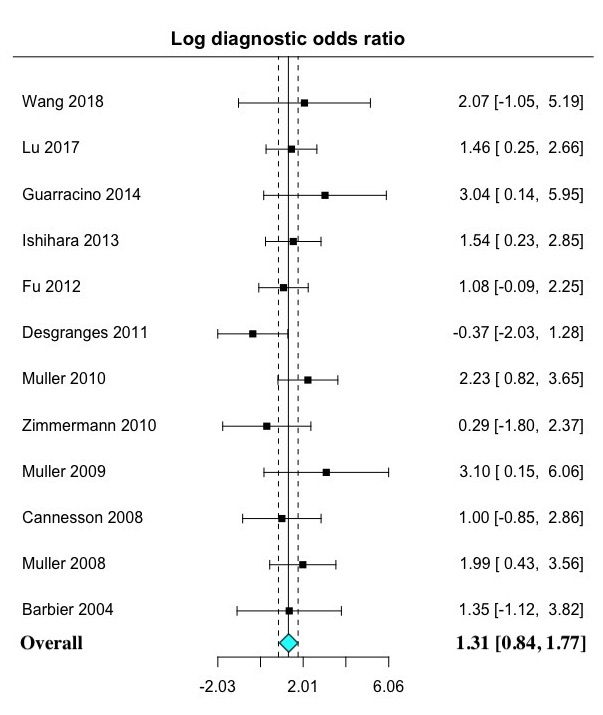


**Figure AF 13.** Forest plot of LnDOR with 95% CI of inferior vena cava variation - ∆IVC.


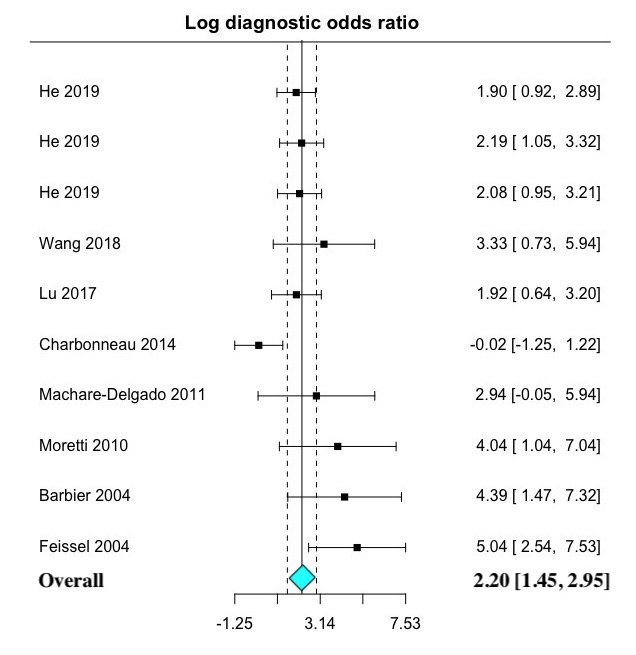


**Figure AF 14.** Bayesian SROC with posterior predictive contour of pulse pressure variation - PPV.


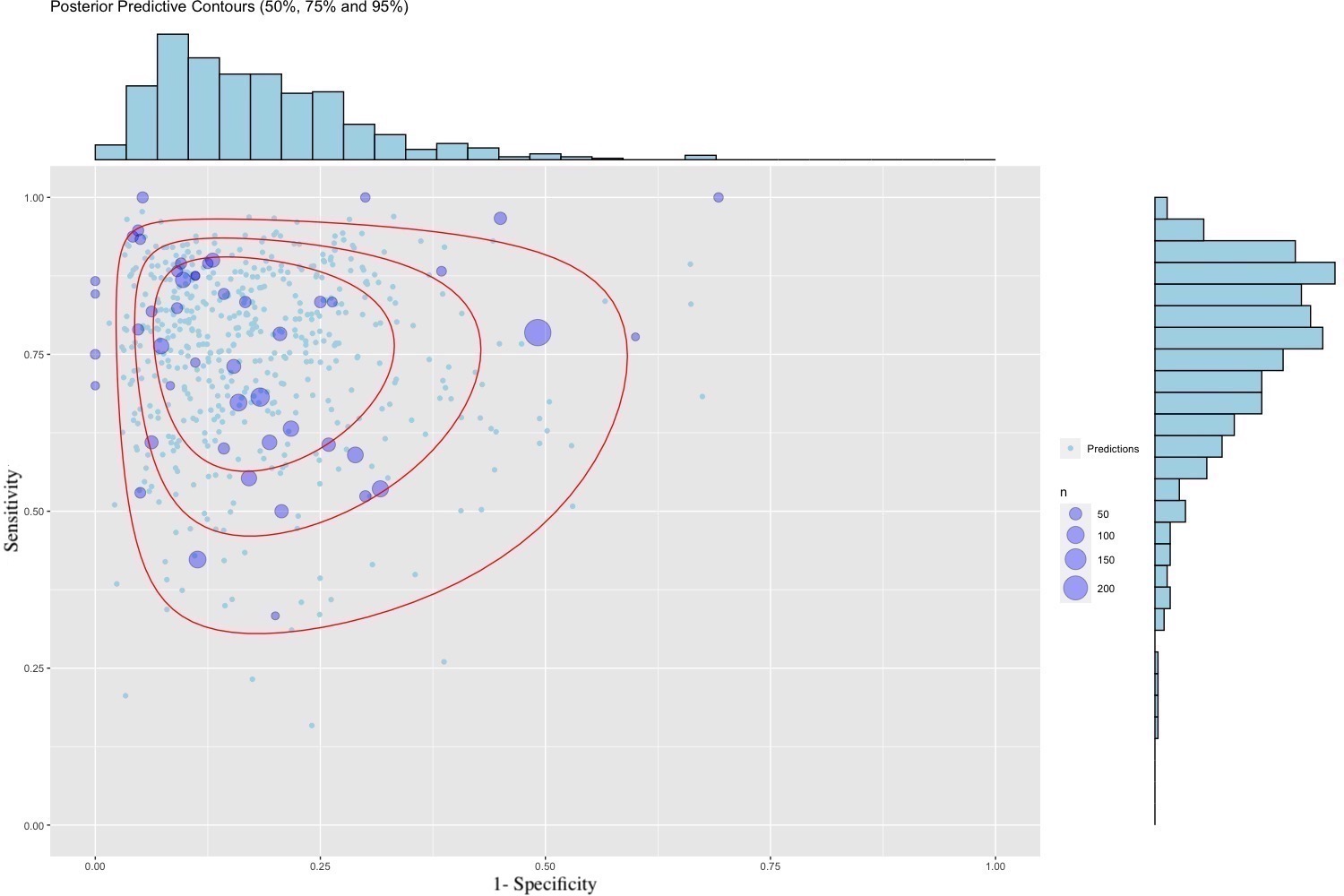


**Figure AF 15.** Bayesian SROC with posterior predictive contour of stroke volume variation - SVV.


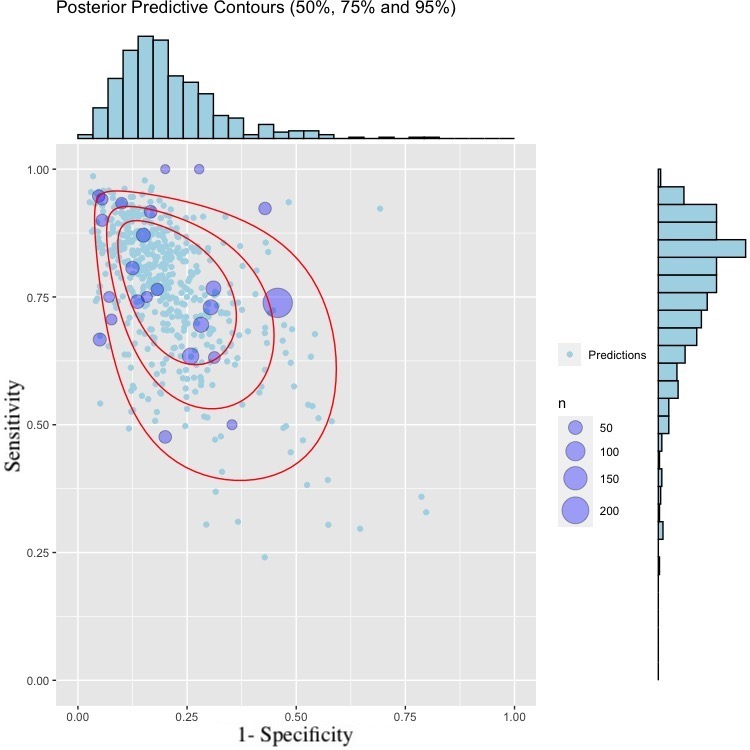


**Figure AF 16**. Bayesian SROC with posterior predictive contour of plethysmographic variability index - PVI.


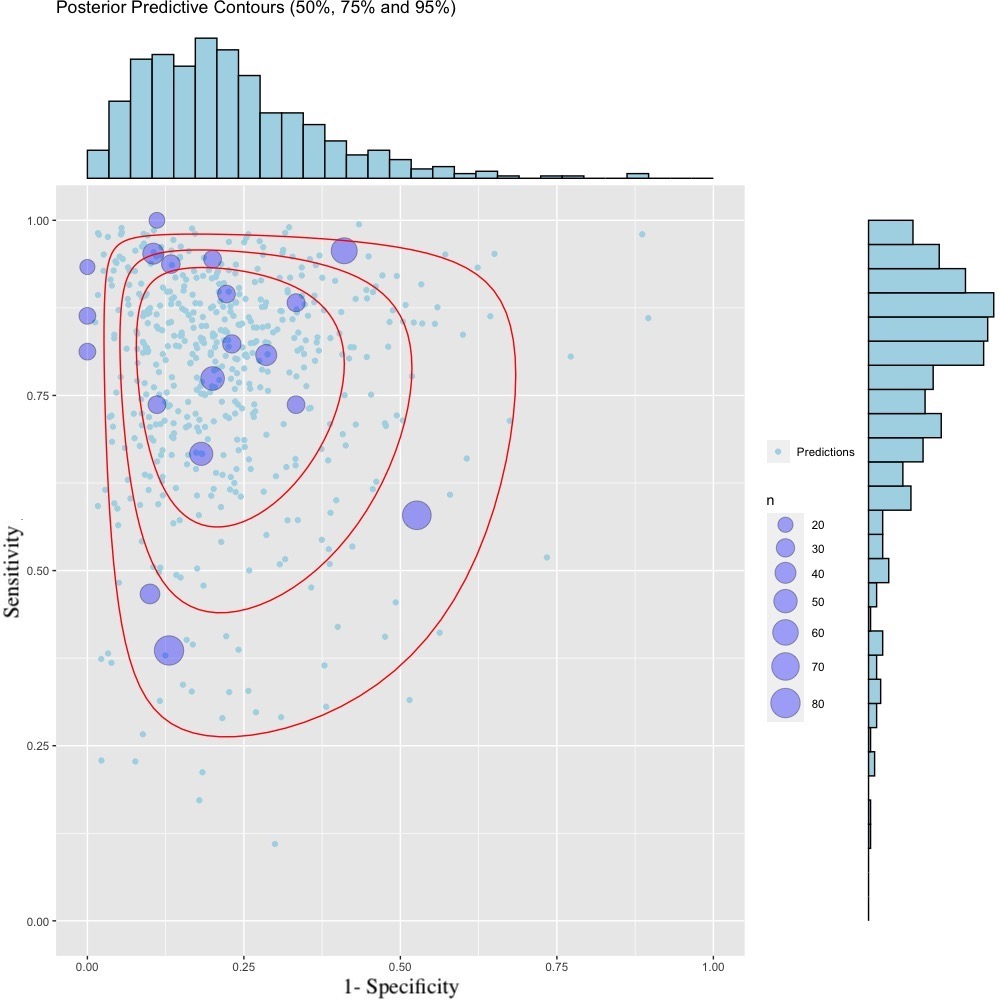


**Figure AF 17.** Bayesian SROC with posterior predictive contour of central venous pressure - CVP.


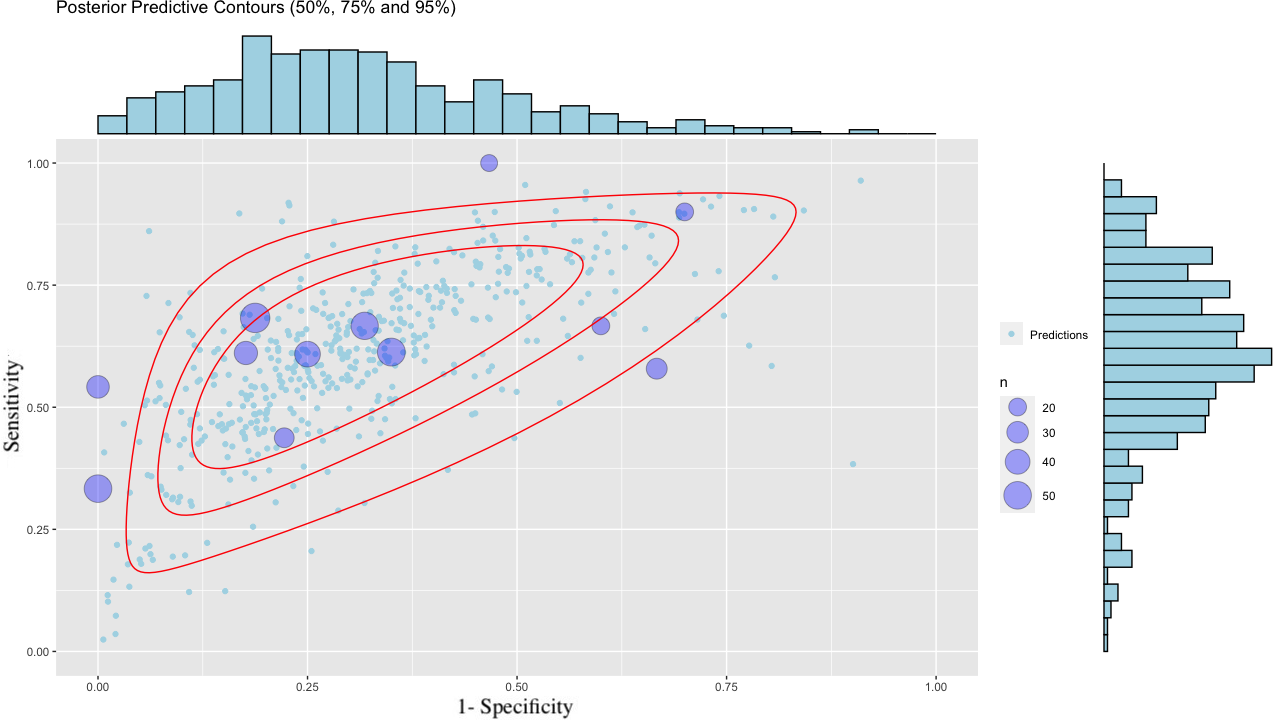


**Figure AF 18.** Bayesian SROC with posterior predictive contour of inferior vena cava variation - ∆IVC.


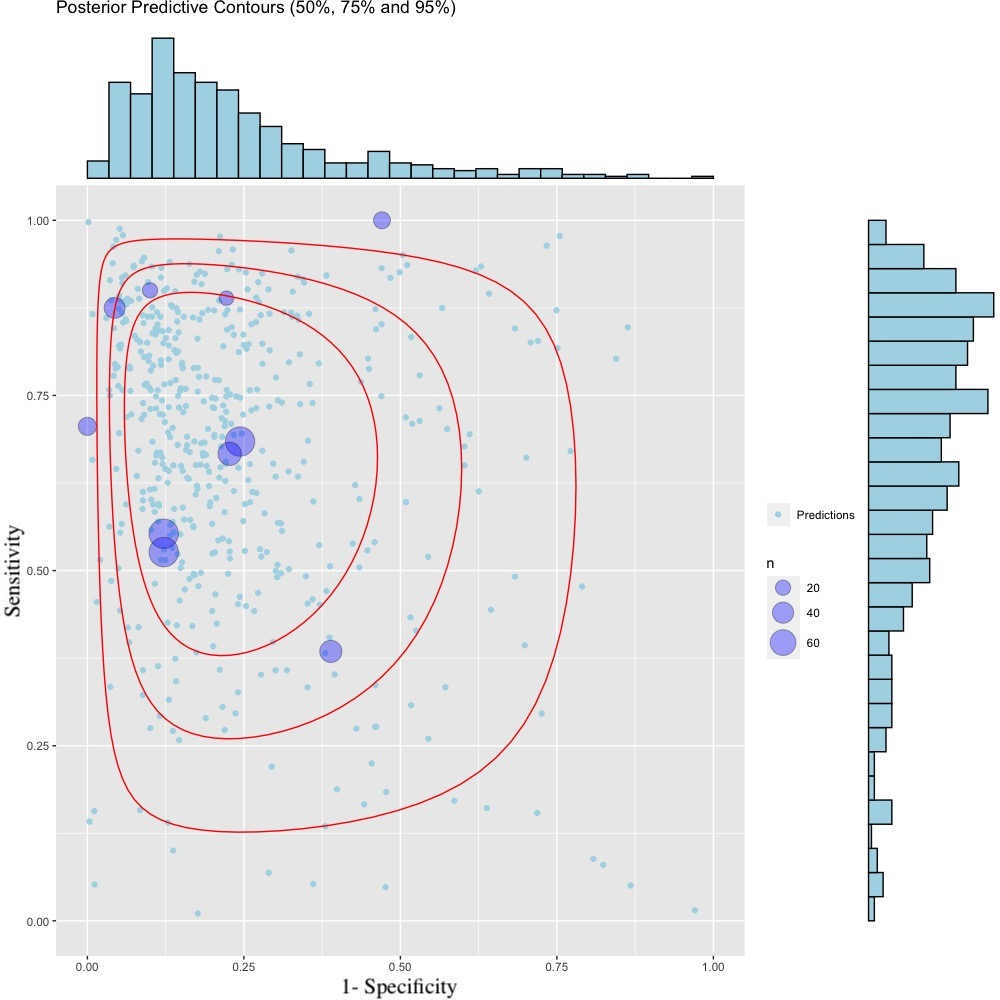


**Figure AF 19.** The use of hydroxyethyl starch (HES) solution and saline solution by year.


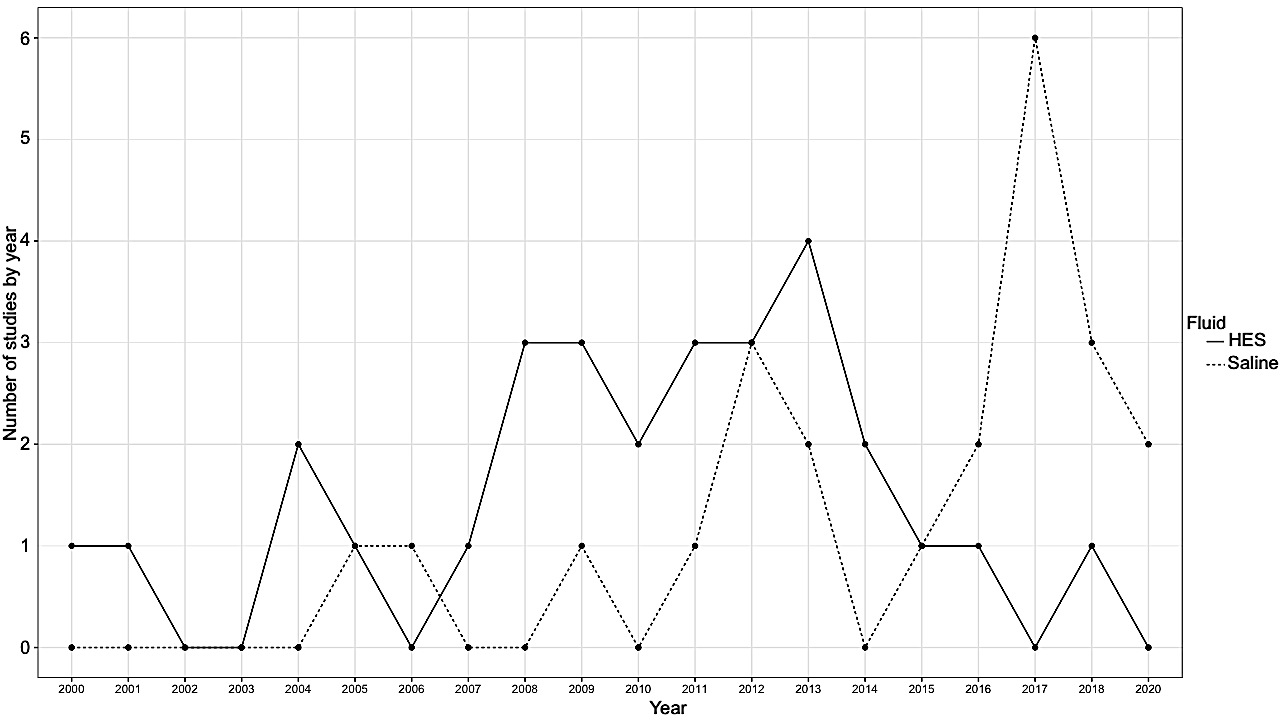


**Figure AF 20.** The use of colloid solution and crystalloid solution by year.


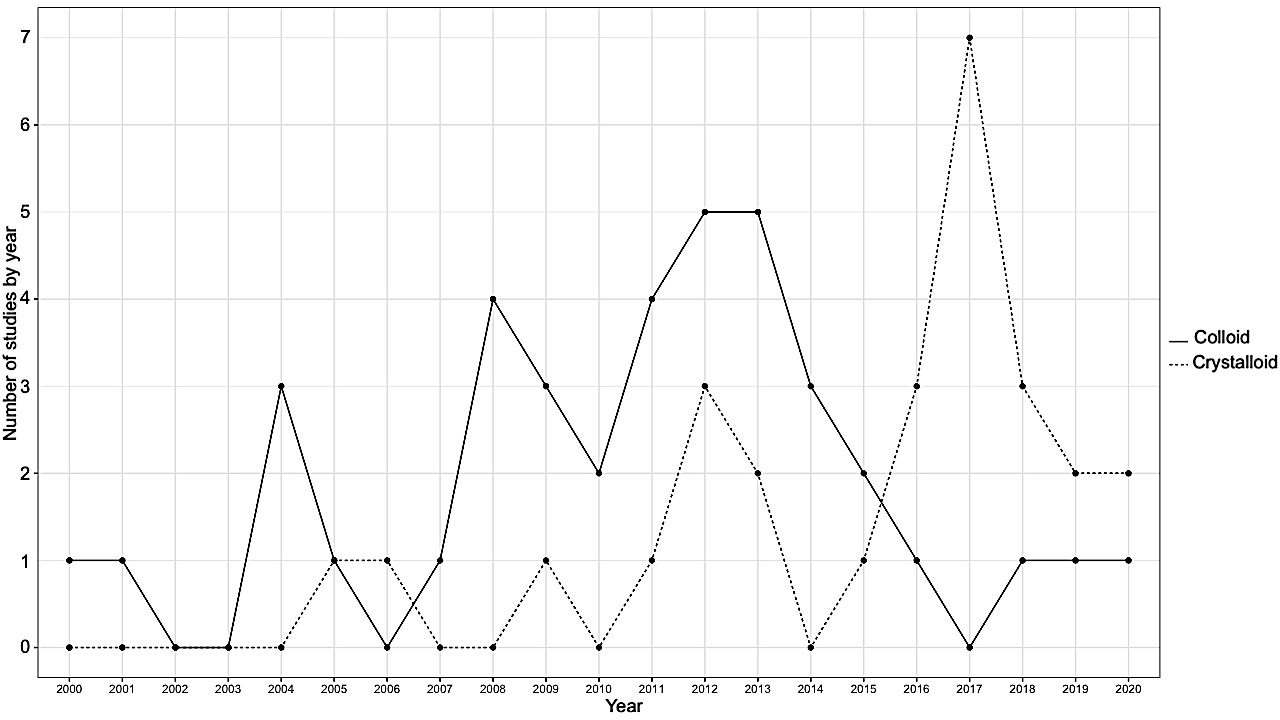


**Table AF 1.** Individual characteristics of included studies.

| **Author, year** | **N** | **Setting** | **Inclusion criteria** | **Exclusion criteria** | **Ventilation** | **Fluid challenge** | **Definition of responders** | **Maneuvers** |
| --- | --- | --- | --- | --- | --- | --- | --- | --- |
| De Courson, 2020 ^(1)^ | 242 (109) | OpR | 1. Age > 18 years  2. Scheduled for neurosurgery in the supine position | 1. COPD  2. Cardiac arrhythmia  3. Right or left heart failure | 1. V_T_ of 6-8 ml/kg IBW  2. PEEP of 6-8 cmH_2_O  3. Volume-controlled mode | 250 ml of saline I.V. over 10 minutes | ↑ SVI >10% | 1. ∆EtCO_2_ = 1.1%  2. PPV = 9.5%  3. SVV = 12.5% |
| Wang, 2020 ^(2)^ | 44 (44) | ICU | 1. Septic shock  2. Age ≥ 18 years | 1. Receiving IABP or ECMO support  2. Cardiac arrhythmia or valvular heart disease  3. Renal failure  4. High abdominal pressure | 1. V_T_ of 8-10 ml/kg | 500 ml of saline I.V. within 15 minutes | ↑ CI ≥15% | 1. ∆VTI = 15.9%  2. SVV = 24.8%  3. PPV = 25.8% |
| Weil, 2020 ^(3)^ | 115 (46) | OpR | 1. Age ≥18 years  2. Scheduled for moderate risk laparotomic surgery | 1. BMI >35 kg/m^2^  2. Laparoscopic surgery  3. Cardiac arrhythmia or right ventricular failure | 1. V_T_ of 8 ml/kg IBW  2. PEEP of 5 cmH_2_O  3. Pressure- controlled mode | 250 ml of colloid I.V. for 5 minutes | ↑ CI >15% | 1. PPV = 14% |
| He, 2019 ^(4)^ | 79 (79) | OpR | 1. General anesthetisia  2. Age 18-60 years  3. ASA grade 1 or 2 | 1. Pregnancy  2. Heart disease  3. Peripheral artery disease or stenosis  4. COPD or pulmonary hypertension or pulmonary embolism  5. High abdominal pressure  6. Receiving IABP | 1. V_T_ was adjusted as part of the protocol | 6 ml/kg of succinyl gelatin I.V. over 10 minutes | ↑ VTI ≥15% | 1. PPV_VT6_ = 8.5%  2. ∆IVC_VT6_ = 11.1%  3. PPV_VT9_ = 12.5%  4. ∆IVC_VT9_ = 15.3%  5. PPV_VT12_ = 15.5%  6. ∆IVC_VT12_ = 13.4% |
| Messina, 2019 ^(5)^ | 40 (40) | OpR | 1. Age >18 years  2. Scheduled for elective spinal surgery | 1. Heart disease  2. Cardiac arrhythmia or valvular heart disease  3. BMI >30 kg/m^2^  4. COPD  5. Preexisting use of β-blocking agents | 1. V_T_ of 6 ml/kg of PBW  2. PEEP between 3 and 6 cm H_2_O  3. Volume-control mode | 250 ml of ringer’s solution I.V. over 10 minutes | ↑ SVI ≥10% | 1. PPV_VT6-8_ = 12.2%  2. SVV_VT6-8_ = 8.0% |
| Georges, 2018 ^(6)^ | 50 (50) | ICU | 1. Sedated patient and undergoing mechanical ventilation  2. Arterial hypotension or  UO < 0.5 ml/kg/h or  mottled skin or  attempt to decrease vasopressor infusion rate | 1. Age <18 years  2. Heart disease  3. Cardiac arrhythmia or valvular heart disease  4. Intra-cranial hypertension  5. Spontaneous breathing activity | 1. V_T_ of 6 to 8 ml/kg IBW  2. Mean PEEP of 6 cm H_2_O  3. Volume-control mode | 500 ml of saline 0.9% I.V over 15 minutes | ↑ CO ≥15% | 1 ∆VTI_EEO_ = 9%  2 ΔVpeak_EEO_ = 8.5% |
| Giraud, 2018 ^(7)^ | 20 (20) | ICU | 1. Fluid challenge was required by the attending physician | 1. Arrhythmia  2. Active bleeding (>100 ml/h)  3. Expected survival of less than 24 hours | 1. Mean VT of 8.5 ml/kg of PBW  2. Mean PEEP of 7.8 cm H_2_O | 500 ml of saline 0.9% I.V. for 10 minutes | ↑ CO ≥15% | 1. ΔSVC = 14.3% |
| Le Guen, 2018 ^(8)^ | 76 (44) | OpR | 1. Adults scheduled for kidney transplantation | 1. Left ventricular ejection fraction <35%  2. BMI <15 or >35 kg/m^2^  3. Cardiac arrhythmia or valvular heart disease  4. Esophageal or aortic disease | 1. V_T_ of 7 ml/kg  2. PEEP of 5 cmH_2_O | 250 ml of saline 0.9% I.V. within 5 minutes | ↑ SV ≥10% | 1. PVI = 10.5% |
| Wang, 2018 ^(9)^ | 18 (18) | ICU | At least one above:  1. heart rate > 100/minutes  2. SBP < 90 mmHg  3. ↓ MAP > 30%  4. UO < 0.5 ml/kg/h  5. Mottled skin | 1. Need protective pulmonary ventilation  2. Cardiac insufficiency  3. Spontaneous breathing | 1. V_T_ of 8 to 12 ml/kg  2. PEEP of 5 - 10 cmH_2_O  3. Assist control mode | 7 ml/kg IBW of 6% HES I.V. over 30 minutes | ↑ CI ≥15% | 1. ΔIVC = 15%  2. CVP = 11 mmHg  3. GEDVI = 517 ml/m^2^  4. ITBVI = 608 ml/m^2^ |
| Biais, 2017 ^(10)^ | 28 (28) | OpR | 1. Age > 18 years  2. Scheduled for neurosurgery  3. Without intracranial hypertension | 1. Require vasopressors or inotropes  2. Infusion of propofol and/or remifentanil was modified | 1. V_T_ of 6 to 8 ml/kg IBW  2. PEEP of 5 cmH_2_O  3. Volume-control mode | 250ml of saline 0.9% I.V. more than 10 minutes | ↑ SV ≥10% | 1. SVV_LRM_ = 30%  2. PPV_LRM_ = 6% |
| Biais, 2017 ^(11)^ | 41 (41) | OpR | 1. Age >18 years  2. Scheduled for neurosurgery | 1. Lung disease  2. Intracranial hypertension  3. Heart disease  4. Arrhythmia  5. BMI <15 or >40 kg/m^2^  6. Require vasopressors or inotropes  7. Infusion of remifentanil or propofol was modified | 1. V_T_ of 6 to 8 ml/kg IBW  2. PEEP of 3 to 5 cmH_2_O  3. Volume-control mode | 250 ml of saline 0.9% I.V. more than 10 minutes | ↑ SV ≥10% | 1. SVV_EEO_ = 5%  2. PPV_EEO_ = 1%  3. PPV = 9% |
| Biais, 2017 ^(12)^ | 88 (44) | OpR | 1. Age >18 years  2. Scheduled for neurosurgery  3. Supine position | 1. Arrhythmia | 1. V_T_ of 6 to 8 ml/kg of IBW  2. PEEP of 3 to 6 cmH_2_O  3. Volume-control mode | 250 ml of saline 0.9% I.V over 10 minutes | ↑ SVI ≥10% | 1. SVVI_50_ = 2%  2. SVVI_100_ = 6%  3. PPV = 10% |
| Jozwiak, 2017 ^(13)^ | 30 (30) | ICU | 1. Mechanically ventilated patients  2. Fluid challenge required by the attending physician | 1. Spontaneous breathing activity impeding the maintenance of respiratory occlusions  2. Arrhythmia  3. Poor echocardiographic echogenicity | 1. Median V_T_ of 6 ml/kg PBW  2. Mean PEEP of 10 cmH_2_O  3. Volume assist-controlled mode | 500 ml of saline I.V. over 10 minutes | ↑ CI >15% | 1. ∆CI_EEO_ = 4%  2. ∆CI_EIO_ = -10%  3. ∆CI_EEO+EIO_ = 11%  4. ∆VTI_EEO_ = 5%  5. ∆VTI_EIO_ = -8%  6. ∆VTI_EEO+EIO_ = 13% |
| Lu, 2017 ^(14)^ | 49 (49) | ICU | 1. Age ≥ 18 years  2. Septic shock | 1. Contraindication to fluid resuscitation  2. Pregnant women  3. Neurogenic shock, cerebrovascular  4. Arrhythmia, heart failure  5. Peripheral vascular disease or stenosis | 1. V_T_ of 8-10 ml/kg  2. PEEP 5-12 cmH_2_O | 200 ml of saline 0.9% I.V. within 10 minutes | ↑ CI ≥10% | 1. CVP = 6.5 mmHg  2. ITBVI = 871 ml/m^2^  3. SVV = 11.5 %  4. PVI = 15.5 %.  5. ∆IVC = 20.5%  6. ∆Vpeak_carot_ = 13%  7. ∆Vpeak_brac_ = 11.7% |
| Myatra, 2017 ^(15)^ | 30 (20) | ICU | 1. Age ≥18 years  2. Acute circulatory failure  3. Mechanical ventilation with low V_T_ without spontaneous breathing  4. Fluid challenge required by the attending physician | 1. Heart disease  2. Air leakage through chest drains  3. Abdominal compartment syndrome  4. Pregnancy  5. Urgently requiring a fluid bolus | 1. V_T_ of 6 ml/kg PBW  2. Mean PEEP of 8.5 cmH_2_O  3. Volume-assist controlled mode | 7 ml/kg of saline I.V. over 10 minutes | ↑ CI>15% | 1. PPV_VT8_ = 11.5%  2. SVV_VT8_ = 10.5%  3. PPV_VT6-8_ = 3.5%  4. SVV_VT6-8_ = 2.5%  5. PPV_7ML/KG_ = 1.5%  6. SVV_7ML/KG_ = 2.5% |
| Yonis, 2017 ^(16)^ | 33 (33) | ICU | 1. ARDS and at least one above:  2. Lactate >2 mmol/L  3. MAP <65 mmHg  4. ↓ cardiac output  5. UO <0.5 ml/kg/h  6. Heart rate >100 min  7. Mottled skin | 1. Age <18 years  2. Contra-indication to the Trendelenburg position  3. Pregnancy  4. Lower limbs amputation  5. Obstruction of inferior vena cava  6. Respiratory effort during EEO | 1. V_T_ of 6 ml/kg of PBW  2. PEEP 5 - 10 cmH_2_O  3. Volume-controlled mode | 500 ml of crystalloids I.V. over 15 minutes | ↑ CI ≥ 15% | 1. ΔCI_TREND_ = 8%  2. PPV_VT6_ = 10%  3. PPV_VT8_ = 9%  4. PPV_VT6-8_ = 29%  5. ΔCI_EEO_ = 10% |
| De Broca, 2016 ^(17)^ | 60 (60) | OpR | 1. Use protective ventilation  2. Fluid challenge in the OpR  3. Hypotension | 1. Ectopic beats  2. Arrhythmia  3. Right ventricular dysfunction  4. Spontaneous ventilation | 1. V_T_ of 6 ml/kg of IBW  2. PEEP of 5-7 cmH_2_O  3. Volume-controlled mode | 500 ml of ringer lactate I.V. over 10 minutes | ↑ SVI ≥15% | 1. SVV_LRM_ = 16%  2. PPV_LRM_ = 25%  3. SVV = 8% |
| Lee, 2016 ^(18)^ | 40 (40) | OpR | 1. Elective arthroscopic shoulder surgery in the beach chair position | 1. End-stage renal disease  2. Heart disease  3. Cerebrovascular disease  4. Age <19 years  5. ASA grade 4 or 5 | 1. V_T_ of 8 ml/kg IBW  2. PEEP of 5 cmH_2_O  3. Volume-controlled mode | 6 ml/kg IBW of 6% HES I.V. for 10 minutes | ↑ SVI ≥15% | 1. SVV = 12%  2. PPV = 15%  3. PVI = 10% |
| Liu, 2016 ^(19)^ | 96 (96) | ICU | 1. Age > 18 years  2. Circulatory failure  3. Hypoperfusion  4. ARDS  5. Fluid challenge required by the attending physician | 1. Hemorrhage  2. Valvular disease, intracardiac shunt or arrhythmia  3. Air leakage through chest drains | 1. V_T_ of 5-8 ml/kg PBW  2. Volume-controlled mode | 500 ml of saline I.V. over 20 minutes | ↑ CO ≥15% | 1. PPV =10%  2. PPV/DP = 0.86%  3. PPV/V_T_ = 1.15%  4. PPV X Ers = 300%  5. PPV/(HR/RR) = 2.12%  6. PPV =12%  7. PPV/Ecw = 0.99%  8. PPV/Ppl = 2% |
| Wu, 2016 ^(20)^ | 62 (31) | OpR | 1. Adult recipients of a living donor orthotopic liver transplant | 1. History of pulmonary resection  2. Chronic respiratory insufficiency  3. Left ventricular ejection fraction < 60%  4. Pulmonary hypertension  5. Arrhythmia | 1. V_T_ of 8 ml/kg  2. PEEP of 5 cmH_2_O | 10 ml/kg of saline I.V. over 15 minutes | ↑ SVI ≥15% | 1. PPV = 10%  2. SVV = 12%  3. PVI = 11% |
| Angappan, 2015 ^(21)^ | 45 (45) | ICU | 1. Acute circulatory failure related to sepsis  2. Relative hemodynamic stability | 1. Arrhythmias  2. Permanent pacemaker  3. Mechanical cardiac support.  4. Hypoxemia | 1. V_T_ < 8 ml/kg  2. Volume-controlled mode | 500 ml of 6% HES I.V. | ↑ CI ≥15% | 1. SVV = 13% |
| Ibarra-Estrada, 2015 ^(22)^ | 59 (19) | ICU | 1. Mechanical ventilation  2. Septic shock  3. Hemodynamic instability  4. Inadequate tissue perfusion | 1. Age <18 years  2. Heart disease  3. Intra-abdominal hypertension  4. Peripheral arterial disease  5. Carotid stenosis > 50%  6. Spontaneous breathing | 1. V_T_ of 6 ml/kg PBW  2. Median PEEP of 6 cmH_2_O  3. Volume-control mode | 7 ml/kg of saline I.V. over 30 minutes | ↑ SVI>15 % | 1. ΔVpeak_carot_ = 14%  2. SVV = 16%  3. SVVI_PLR_ = 15%  4. PPV = 14% |
| Mallat, 2015 ^(23)^ | 49 (49) | ICU | At least one of the following:  1. SBP <90 mmHg  2. MAP <65 mmHg  3. Vasopressor infusion  4. Mottled skin  5. lactate >2 mmol/L  6. UO < 0.5 ml/kg/h | 1 V_T_ ≥8 ml kg of IBW  2. Pregnancy  3. Age <18 years  4. Moribund  5. Arrhythmias  6. Risk of pulmonary oedema | 1. Median V_T_ of 6.8 ml/kg IBW  2. Median PEEP of 8 cmH_2_O | 500 ml of 4% albumin I.V. during 15 minutes | ↑ CI ≥15% | 1. ΔCI_100_ = 5.2%  2. SVV_100_ = -2%  3. PPV_100_ = -2% |
| Charbonneau, 2014 ^(24)^ | 44 (44) | ICU | 1. Mechanically ventilated patients  2. Septic shock  3. Fluid challenge required by the attending physician | 1. Spontaneous breathing  2. Arrhythmias  3. Echocardiographic could not be performed | 1. V_T_ of 8 to 10 ml/kg  2. Median PEEP of 7 cmH_2_O  3. Volume controlled mode | 7 ml/kg of 6% HES I.V. for 15 minutes | ↑ CI ≥15% | 1. ΔSVC = 29%  2. ΔIVC = 21% |
| Guarracino, 2014 ^(25)^ | 50 (50) | ICU | 1. Sepsis  2. Age > 18 years  3. Mechanically ventilated | 1. Cardiac disease  2. Jugular vein thrombosis  3. Atrial fibrillation | 1. V_T_ of 6 to 8 ml/kg  2. Median PEEP of 6 cmH_2_O | 7 ml/kg of crystalloid I.V. for 30 minutes | ↑ CI ≥15% | 1. PPV = 12.5%  2. CVP = 8 mmHg  3. ΔIJV =18%  4. ΔIJV = 9.9% and PPV = 12% |
| Siswojo, 2014 ^(26)^ | 29 (29) | OpR | 1. Noncardiac surgery under general anesthesia and mechanical ventilation | 1. Arrhythmias  2. Ischemic heart disease  3. Cardiac failure  4. Contraindications to esophageal probe insertion | 1. V_T_ of 8 ml/kg IBW  2. PEEP of 0 cmH_2_O  3 volume- controlled mode | 500 ml of HES I.V. over 5 minutes | ↑ SVI ≥10% | 1. PVI = 10.5% |
| Feissel, 2013 ^(27)^ | 31 (31) | ED | 1. Age > 18 years  2. Sinus rhythm  3. Early phase of septic shock  4. Persistent hypotension or oliguria or metabolic acidosis | 1. Left ventricular ejection fraction less than 50%,  2. Echocardiography images insufficient  3. PVI value not displayed | 1. V_T_ of 8-10 ml/kg PBW  2 Volume- controlled mode | 8 ml/kg of 6% HES I.V. over 20 minutes | ↑ VTI ≥15% | 1. PVI = 19% |
| Fischer, 2013 ^(28)^ | 80 (80) | ICU | 1. Elective cardiac surgery  2. Fluid challenge during the initial postoperative period | 1. Emergency surgery  2. Arrhythmia  3. Spontaneous ventilation or tidal volume <7 ml/kg  4. Intracardiac shunt  5. Thoracic compliance < 30 ml/cmH_2_O  6. Abdominal hypertension | 1. Mean V_T_ of 8.2 ml/kg  2. Mean PEEP of 0 cmH_2_O  3. Volume-controlled mode | 500 ml of 6% HES I.V. over 15 minutes | ↑ CI ≥15% | 1. PPV = 14%  2. PVI = 20% |
| Freitas, 2013 ^(29)^ | 40 (40) | ICU | 1. Age >18 years  2. Severe sepsis or septic shock  3. Sedated patient and undergoing mechanical ventilation with a low V_T_  4. Required fluid challenge by the attending physician | 1. Cardiac arrhythmias, valvular disease or intracardiac shunt  2. Acute bleeding  3. Abdominal compartment syndrome  4. Pregnancy | 1. V_T_ of 6 ml/kg PBW  2. Median PEEP of 10 cmH_2_O  3. Volume-controlled mode | 7 ml/kg of HES I.V. over 30 minutes | ↑ CO ≥15% | 1. PPV_VT6_ = 6.5%  2. PPV_VT8_ = 12.3% |
| Ishihara, 2013 ^(30)^ | 43 (43) | ICU | 1. Hypotension  2. Postoperative period of Abdominothoracic  esophagectomy | 1. Aortic aneurysms  2. Sustained arrhythmias | 1. V_T_ >8 ml/kg of IBW  2. PEEP <5 cmH_2_O  3. Controlled mechanical ventilation | 250 ml of 10% dextran I.V. over 20 minutes | ↑ CI >15% | 1. SVV = 10.5 %  2. PPV = 8.5%  3. CVP = 6.5 mmHg  4. ITBVI = 0.78 L/m^2^  5. IDVGI = 4.23 L/m^2^ |
| Monnet, 2013 ^(31)^ | 40 (40) | ICU | 1. Hemodynamic instability  2. Mechanical ventilated without inspiratory effort | 1. Age <18 years  2. Head trauma  3. Deep vein thrombosis of the inferior limbs | 1. Mean V_T_ of 6.4 ml/kg PBW  2. Mean PEEP of 7 cmH_2_O  3. Control assisted mode | 500 ml of saline I.V. over 30 minutes | ↑ CI ≥15 % | 1. ΔCI_PLR_ = 10%  2. ΔEtCO_2PLR_ = 5% |
| Monnet, 2013 ^(32)^ | 35 (35) | ICU | 1. Received norepinephrine  2. Acute circulatory failure  3. Fluid challenge was required by the attending physician | 1. Cardiac arrhythmias  2. Spontaneous triggering of the ventilator  3. V_T_ <8 ml/kg of PBW  4. Compliance of respiratory system was ≤30 cmH_2_O | 1. Mean V_T_ of 9 ml/kg of PBW  2. Mean PEEP of 6.6 cmH_2_O  3. Volume-controlled mode | 500 ml of saline I.V. over 30 minutes | ↑CI ≥15% | 1. PPV = 11%  2. SVV = 10%  3. PVI = 16% |
| Trepte, 2013 ^(33)^ | 72 (24) | OpR | 1. Age >18 years  2. Undergoing major surgery  3. Controlled mechanical ventilation | 1. History of lung surgery or pneumothorax  2. Cardiac arrhythmias  3. Renal failure | 1. V_T_ of 8 ml/kg  2. PEEP of 5 cmH_2_O  3. Pressure- controlled mode | 300 ml of HES I.V. | ↑ CI ≥10% | 1. PPV = 10.1%  2. SVV = 9.9%  3. RSVT = 19.7^0^ |
| Vos, 2013 ^(34)^ | 30 (30) | OpR | 1. Patients undergoing major hepatic resection  2. ASA grade 1, 2, and 3 | 1. Incurable disease  2. Cardiac dysrhythmia  3. Haemodynamic instability | 1. V_T_ of 8 ml/kg lean body mass  2. PEEP of 5 cmH_2_O  3. Volume- controlled mode | 15 ml/kg of fluid (crystalloid or colloid) I.V. in 30 minutes | ↑ SVI ≥20% | 1. SVV = 15%  2. PPV = 14%  3. PVI = 12% |
| Biais, 2012 ^(35)^ | 35 (35) | ICU | At least one above:  1. SBP <90 mmHg  2. UO <0.5 ml/kg/h  3. HR >100 beats/min  4. Mottled skin | 1. Age < 18 years  2. Arrhythmia or cardiac disfunction  3. V_T_ of 8 ml/kg  4. Spontaneous breathing  5. Unsatisfactory cardiac echogenicity  6. High abdominal pressure | 1. V_T_ of 8-10 ml/kg PBW  2. PEEP ≤ 5 cmH_2_O  3. Volume- controlled mode | 500 ml of saline I.V. over 15 minutes | ↑ SV ≥15% | 1. SVV = 12.6%  2. PPV = 10% |
| Cecconi, 2012 ^(36)^ | 31 (31) | ICU | 1. Admission to the ICU following high-risk surgery  2. Sedated patient and undergoing mechanical ventilation | 1. Spontaneous breathing  2. Arrhythmia  3. Pre-existing therapeutic lithium use  4. Aortic regurgitation | 1. V_T_ of 8 ml/kg IBW  2. PEEP of 5 cmH_2_O  3. Pressure- controlled mode | 250 ml of colloid I.V. over 5 minutes | ↑ SV >15% | 1. ΔSBP = 9%  2. PPV =13%  3. SVV = 12.5% |
| Fu, 2012 ^(37)^ | 51(51) | OpR | 1. Patients undergoing resection of primary retroperitoneal tumors | 1. Age < 18 years  2. Arrhythmias  3. Intracardiac shunts | 1. V_T_ of 8 to 10 ml/kg  2. PEEP of 0 cmH_2_O  3. Volume- controlled mode | 8 ml/kg of I.V. HES between 20 to 30 min | ↑ SVI ≥10% | 1. SVV = 12.5%  2. PVI = 13.5%  3. SVI = 43.5 ml/m^2^  4. CI = 2.85 L/min/m^2^  5. CVP = 7.5 mmHg  6. MAP = 67.5 mmHg  7. PI = 3.49% |
| Haas, 2012 ^(38)^ | 22 (22) | OpR | 1. Elective cardiac surgery with the use of cardiopulmonary bypass | 1. Atrial fibrillation  2. Arterial occlusive disease of the upper limb. | 1. V_T_ of 8 ml/kg  2. PEEP of 5 cmH_2_O | 4 ml/kg of colloid I.V. | ↑ CI ≥10% | 1. PVI = 16%  2. SVV = 11% |
| Khwannimit, 2012 ^(39)^ | 42 (42) | ICU | 1. Septic shock  2. Patient undergoing mechanical ventilation  3. Require a rapid volume challenge | 1. Acute pulmonary oedema  2. Arrhythmia or cardiac disfunction  3. Severe peripheral vascular disease  4. Spontaneous breathing efforts | 1. V_T_ ≥ 8 ml/kg  2. Controlled mode | 500ml of 6% HES I.V. over 30 minutes | ↑ SVI ≥15% | 1. SVV = 10%  2. PPV = 12% |
| Monge García, 2012 ^(40)^ | 37 (37) | ICU | 1. Patient undergoing control mechanical ventilation and at least one above:  2. SBP ≤90 mmHg  3. Need of vasopressor  4. UO ≤0.5 ml/kg/h  5. HR >100 beats/min  6. Mottled skin  7. Delayed CRT | 1. Age <18 years  2. Pregnancy  3. Contraindication for the use of esophageal Doppler  4. Contraindication to perform passive leg raising | 1. Mean V_T_ of 8.1 ml/kg PBW  2. Median PEEP of 6.4 cmH_2_O  3. Volume- controlled mode | 500 ml of 6% HES I.V. over 30 minutes | ↑ CO ≥15% | 1. ΔCO_PLR_ = 12%  2. ΔEtCO_2PLR_ = 5%  3. FTc = 300ms  4. PPV_PLR_ = 11% |
| Monnet, 2012 ^(41)^ | 54 (54) | ICU | 1. Circulatory failure  2. Fluid challenge was required by the attending physician  3. Patient undergoing control assisted mechanical ventilation | 1. Age < 18 years  2. Moribund  3. Passive leg raising was contraindicated  4. Absence of cardiac arrhythmias and spontaneous triggering of the ventilator | 1. Mean V_T_ of 7.9 ml/kg PBW  2. Mean PEEP of 6.5 cmH_2_O  3. Volume-controlled mode | 500 ml of saline I.V. over 20 minutes | ↑ CI ≥15% | 1. PPV_crs>30_ = 12%  2. ΔCI_PLRcrs>30_ = 10%  3. ΔCI_EEOcrs>30_ = 5%  4. PPV_crs≤30_ = 4%  5. ΔCI_PLRcrs≤30_ = 10%  6. ΔCI_EEO crs≤30_ = 5% |
| Monnet, 2012 ^(42)^ | 39 (39) | ICU | 1. Acute circulatory failure  2. Inadequate tissue perfusion | 1. Cardiac arrhythmias  2. Spontaneous triggering of the ventilator  3. Hydrostatic pulmonary oedema | 1. Mean V_T_ of 7.9 ml/kg  2. Mean PEEP of 6.4 cmH_2_O  3. Volume-controlled mode | 500 ml of saline I.V. over 30 minutes | ↑ CI ≥15% | 1. SVV = 14%  2. PPV = 10%  3. PPVni = 11%  4. ΔCI_PLR_ = 11%  5. ΔCI_EEO_ = 5% |
| Oliveira-Costa, 2012 ^(43)^ | 37 (37) | ICU | 1. Age ≥16 years  2. Hemodynamic instability | 1. Cardiac arrhythmia  2. Pneumothorax  3. Heart valve disease  4. Intracardiac shunt  5. Right ventricular insufficiency | 1. V_T_ <8 ml/kg IBW  2. Median PEEP < 8 cmH_2_O | 1000 ml of saline or lactated Ringer’s or 500ml of 6% HES I.V. for 30 minutes | ↑ CI ≥15% | 1. PPV = 10% |
| Desgranges, 2011 ^(44)^ | 28 (28) | OpR | 1. Patients referred for cardiac surgery | 1. Cardiac arrhythmias  2. Left ventricular ejection fraction <40%  3. Right ventricular dysfunction | 1. V_T_ of 8 ml/kg  2. PEEP of 0 cmH_2_O  3. Volume-controlled mode | 500 ml of 6% HES I.V. over 10 minutes | ↑ CI ≥15% | 1. PVI = 12%  2. PVI_EAR_ = 16%  3. PVI_FOREHEAD_ = 15%  4. PI = 4.2%  5. PI_EAR_ = 0.36  6. PI_FOREHEAD_ = 1.37%  7. PPV = 11%  8. CVP = 8mmHg |
| Hood, 2011 ^(45)^ | 25 (25) | OpR | 1. ASA grade 1, 2, and 3  2. Age ≥18 years  3. Elective major colorectal surgery  4. Oxygen uptake at anaerobic threshold >11 ml/kg/min on preoperative testing | 1. Needed an arterial catheter or central venous catheter for intraoperative monitoring | 1. V_T_ of 8 to 10 ml/kg  2. PEEP of 0 cmH_2_O  3. volume-controlled mode | 500 ml of HES I.V. rapidly via pressurized infusion | ↑ SV >10% | 1. PVI = 10% |
| Lakhal, 2011 ^(46)^ | 65 (65) | ICU | 1. Adults  2. Acute circulatory failure  3. ARDS | 1. Arrhythmia  2. Diuretic treatment  3. Uncontrolled hemorrhage  4. Brain death  5. Receiving IABP  6. Risk of fluid loading  7. Hypoxemia | 1. Mean V_T_ <6.5 ml/kg PBW  2. Mean PEEP < 8.5 cmH_2_O  3. Volume-controlled mode | 500 ml of fluid gelatin I.V. over 30 minutes | ↑ CO >10% | 1. PPV = 5% |
| Loupec, 2011 ^(47)^ | 40 (40) | ICU | 1. Age >17 yeas  2. Clinical sign of inadequate tissue perfusion | 1. Spontaneous respiratory activity  2. Cardiac arrhythmia  3. Intracardiac shunt  4. Contraindication for PLR  5. Left ventricular ejection fraction <50%  6. Hemodynamic instability during the procedure | 1. V_T_ of 8-10 ml/kg PBW  2. PEEP ≤ 8 cmH_2_O  3. Volume-controlled mode | 500 ml of HES I.V. infused in 10 minutes | ↑ CO ≥15% | 1. PVI = 17%  2. PPV = 10% |
| Machare-Delgado, 2011 ^(48)^ | 25 (25) | ICU | 1. Sedated patient and undergoing mechanical ventilation without asynchronous  2. Using vasopressor  3. Low blood pressure or poor organ perfusion | 1. Patient on hemodialysis  2. Ascites  3. Atrial fibrillation  4. Rapidly changing vasopressor doses  5. Spontaneous modes of mechanical ventilation | 1. V_T_ of 8 ml/kg of PBW  2. Mean PEEP of 6.8 cmH_2_O  3. Assist-control mode | 500 ml of saline I.V. over 10 minutes | ↑ SVI ≥10% | 1. ΔIVC = 12%  2. SVV = 12% |
| Moretti, 2010 ^(49)^ | 29 (29) | ICU | 1. Subarachnoid hemorrhage (Fisher group 3 and 4)  2. Sedated patient and undergoing mechanical ventilation | 1. Age <18  2. Heart failure or cardiac arrhythmias  3. ARDS  4. Extravascular lung water (ELWI) >14 ml/kg | 1. V_T_ of 8 ml/kg  2. PEEP of 0 cmH_2_O  3. Volume-controlled mode | 7 ml/kg of 6% HES I.V. over 30 minutes | ↑ CI ≥15% | 1. ΔIVC = 16% |
| Muller, 2010 ^(50)^ | 57 (57) | ICU | 1. Acute circulatory failure  2. Signs of hypoperfusion  3. Sedated patient and undergoing mechanical ventilation | 1. Cardiac arrhythmias  2. Tricuspid insufficiency  3. Ventricular dysfunction  4. Moribund  5. Parturient  6. Age <18 years | 1. Median V_T_ < 6 ml/kg IBW  2. Median PEEP < 6 cmH_2_O | 250 or 500 ml of saline or HES I.V. between 15 to 30 minutes | ↑ SVI ≥15% | 1. PPV = 7%  2. CVP = 9mmHg |
| Zimmermann, 2010 ^(51)^ | 20 (20) | OpR | 1. Elective major abdominal surgery | 1. Heart disease  2. Intracardiac shunts  3. Peripheral vascular disease  4. Preoperative dysrhythmias | 1. V_T_ of 7 ml/kg  2. PEEP of 5 cmH_2_O  3. Volume-controlled mode | 7 ml/kg of 6% HES I.V. in 7 minutes | ↑ SVI ≥15% | 1. SVV = 11%  2. PVI = 9.5%  3. CVP = 10.5 mmHg |
| Monge García, 2009 ^(52)^ | 38 (38) | ICU | At least one above:  1. SBP <90 mmHg  2. Need vasopressor  3. UO <0.5 ml/kg/min  4. Tachycardia  5. Delayed CRT  6. Mottled skin | 1. Fluid overload  2. Pulmonary edema  3. Arrhythmia | 1. V_T_ of 8 - 10 ml/kg  2. PEEP of 4 - 6 cmH_2_O  3. Controlled-volume mode | 500 ml of 6% HES I.V. over 30 minutes | ↑ SVI ≥15% | 1. ΔVpeak_brac_ = 10%  2. PPV =10%  3. ΔSV = 11% |
| Monnet, 2009 ^(53)^ | 34 (34) | ICU | 1. Patient undergoing mechanical ventilation and at least one above:  2. SBP <90 mmHg  3. Need vasopressor  4 UO <0.5 ml/kg/h  4. Heart rate >100/min  5. Mottling skin | 1. Inspiratory effort during the end-expiratory occlusion | 1. Mean V_T_ of 6.8 ml/kg  2. Mean PEEP of 8 cm H_2_O  3. Volume assist-controlled mode | 500 ml of saline I.V. for 10 minutes | ↑ CI >15% | 1. PPV_EEO_ of 5%  2. ΔCI_EEO_ = 5%  3. ΔSBP_EEO_ = 4%  4. ΔCI_PLR_ = 10%  5. PPV_PLR_ = 11%  6. CI = 2.8 L/min/m^2^ |
| Muller, 2009 ^(54)^ | 33 (33) | ICU | 1. Sedated patient and undergoing mechanical ventilation  2. Acute circulatory failure  3. Fluid challenge was indicated by the attending physician | 1. Severe cardiac failure  2. Cardiogenic pulmonary edema  3. Age < 18 years  4. Pregnant | 1. Median V_T_ <7.3 ml/kg  2. Median PEEP <8 cmH_2_O | 250 or 500 ml of saline or HES I.V. between 15 to 30 minutes | ↑ SVI ≥15% | 1. BNP = 193 pg/ml  2. CVP = 7 mmHg |
| Vallée, 2009 ^(55)^ | 84 (84) | ICU | 1. Patient undergoing mechanical ventilation  2. Fluid challenge required according to the attending physician | 1. Cardiac arrhythmia | 1. Mean V_T_ of 7.8 ml/kg  2. Mean PEEP of 5 cmH_2_O  3. Controlled mode | 6 ml/kg of HES I.V. over 30 minutes | ↑ CI >15% | 1. PPV = 15%  2. PPV/DP = 0.9 |
| Biais, 2008 ^(56)^ | 35 (35) | ICU | 1. Postoperative period of liver transplantation  2. Signs of acute circulatory failure | 1. Hypoxemia  2. Blood volume overload  3. Age <18 years  4. Arrhythmias  5. Aortic or mitral valvulopathy  6. Intracardiac shunt  7. BMI >40 or <15 kg/m^2^  8. Spontaneous breathing | 1. V_T_ of 8 - 10 ml/kg  2. PEEP of 3 cmH_2_O  3. Volume-controlled mode | 20 ml/BMI of 4% albumin I.V. over 20 minutes | ↑ CO ≥15% | 1. SVV = 10% |
| Cannesson, 2008 ^(57)^ | 25 (25) | OP | 1. Patients undergoing coronary artery bypass grafting | 1. Cardiac arrhythmias  2. Intracardiac shunt | 1. V_T_ of 8 - 10 ml/kg  2. PEEP of 0 - 2 cmH_2_O  3. Volume-controlled mode | 500 ml of 6% HES I.V. give more than 10 minutes | ↑ CI ≥15% | 1. PPV = 12.5%  2. ΔPOP = 12%  3. PPVa = 10.5%  4. PVI = 14%  5. CVP = 12.5 mmHg  6. PCWP = 14.5 mmHg  7. CI = 2.8 L/min/m^2^  8. PI = 1.43% |
| Huang, 2008 ^(58)^ | 22 (22) | ICU | 1. ARDS  2. Acute Lung Injury score greater than 2.5 | Not clearly described | 1. Mean V_T_ of 6.4 ml/kg  2. Mean PEEP of 14 cmH_2_O  3. Pressure-controlled mode | 500 ml of 10% HES I.V. at a rate of 10ml/kg/h | ↑ CI ≥15% | 1. PPV = 11.8% |
| Muller, 2008 ^(59)^ | 35 (35) | ICU | 1. Acute circulatory failure  2. Signs of inadequate tissue perfusion | 1. Cardiac arrhythmias  2. Tricuspid insufficiency  3. Ventricular dysfunction  4. Parturient  5. Age <18 years | 1. Median V_T_ <8 ml/kg IBW  2. Median PEEP <6 cmH_2_O | 250 or 500 ml of HES I.V. at a rate of 999ml/h | ↑ SVI ≥15% | 1. CVP = 9 mmHg  2. ITBVI = 928 ml/m^2^ |
| Feissel, 2007 ^(60)^ | 28 (23) | ICU | 1. Mechanically ventilated patients  2. Septic shock  3. Fluid challenge required according to the attending physician | 1. Heart valve disease  2. Inspiratory efforts  3. Cardiac arrhythmias | 1. V_T_ of 8 - 10 ml/kg | 8 ml/kg of 6% HES I.V. | ↑ CI ≥15% | 1. PPV = 12%  2. PVI = 14% |
| Lafanechère, 2006 ^(61)^ | 22 (22) | ICU | 1. Sedated patient and undergoing mechanical ventilation  2. Acute circulatory failure  3. Fluid challenge required according to the attending physician | 1. Spontaneous breathing activity  2. Cardiac arrhythmias  3. Contraindication for the use of esophageal doppler  4. Incapacity to practice PLR | 1. Median V_T_ of 7 ml/kg  2. Median PEEP of 8 cmH_2_O  3. Volume-controlled mode | 500 ml of saline I.V. | ↑ Aortic blood flow >15% | 1. ΔABF_PLR_ = 8%  2. PPV = 12%  3. LVETc = 245 ms |
| De Backer, 2005 ^(62)^ | 60 (60) | ICU | 1. ARDS  2. Hypovolemia  3. Mechanically ventilated without spontaneous respiratory movements | 1. Cardiac arrhythmias  2. Heart valve disease  3. Intracardiac shunt | 1. Median V_T_ <7.5 ml/kg  2. Median PEEP < 6 cmH_2_O | 1000 ml of crystalloids or 500 ml of HES I.V. over 30 minutes | ↑ CI ≥15% | 1. PPV = 11.8% |
| Feissel, 2005 ^(63)^ | 22 (20) | ICU | 1. Septic shock  2. Potentially needing a volume challenge | 1. Heart valve disease  2. Contraindication to a fluid challenge  3. Inspiratory efforts  4. Absence of sinus rhythm  5. Hemodynamic instability | 1. V_T_ of 8 - 10 ml/kg  2. Controlled mode | 8 ml/kg of 6% HES I.V. | ↑ CI ≥15% | 1. PPV = 17%  2. ΔPEP_KT_ = 4%  3. ΔPEP_PLET_ = 4% |
| Monnet, 2005 ^(64)^ | 38 (38) | ICU | 1. Mechanically ventilated patients and at least one above:  2. SBP <90 mmHg  3. Need of vasopressors  4. UO <0.5 ml/kg/min  5. Tachycardia  6. Mottled skin | 1. PaO_2_/FIO_2_ <100 mmHg  2. Evidence of blood volume overload  3. Spontaneous breathing  4. Cardiac arrhythmias  5. Contraindication for esophageal Doppler monitoring | 1. V_T_ of 6 - 10 ml/kg  2. PEEP of 2 - 10 cmH_2_O  3. Volume-controlled mode | 500 ml of saline 0.9% I.V. over 10 minutes | ↑ Aortic blood flow ≥15% | 1. FTc = 277 ms  2. ΔABF =18%  3. ΔVpeak_aor_ = 13% |
| Barbier, 2004 ^(65)^ | 20 (20) | ICU | 1. Mechanically ventilated patients  2. Age >18 years  3. Severe sepsis  4. Circulatory failure | 1. Poor response to echocardiography rendered | 1. V_T_ of 7 - 10 ml/kg  2. PEEP of 2 - 6 cmH_2_O  3. Volume controlled mode | 7 ml/kg of 4% gelatin I.V. over 30 minutes | ↑ CI ≥15% | 1. ΔIVC = 18%  2. CVP = 12 mmHg |
| Feissel, 2004 ^(66)^ | 39 (39) | ICU | 1. Septic shock  2. Clinical requirement for a rapid volume challenge  3. Acute circulatory failure | 1. Life-threatening hypoxemia  2. Echocardiographic evidence of right ventricular failure | 1. V_T_ of 8 - 10 ml/kg  2. volume-controlled mode | 8 ml/kg of 6% HES I.V. over 20 minutes | ↑ CO ≥15% | 1. ΔIVC = 12% |
| Vieillard-Baron, 2004 ^(67)^ | 66 (66) | ICU | 1. Sepsis  2. Acute circulatory failure  3. Mechanical ventilation  4. SDRA  5. Without spontaneous breathing efforts | 1. Inadequate Doppler aortic signal  2. Cardiac arrhythmias | 1. Mean V_T_ of 8 ml/kg  2. PEEP of 5 - 7 cmH_2_O  3. Controlled mode | 10 ml/kg of 6% HES I.V. over 30 minutes | ↑ CI >11% | 1. ΔSVC = 36%  2. PPV = 12% |
| Feissel, 2001 ^(68)^ | 19 (19) | ICU | 1. Septic shock  2. Mechanical ventilation  3. Hemodynamic stability | 1. Arrhythmias  2. Severe hypoxemia  3. Contraindication to transesophageal echocardiography  4. Aortic valvulopathy  5. Left ventricular dysfunction | 1. V_T_ of 8 - 10 ml/kg  2. Controlled-volume mode | 8 ml/kg of 6% HES I.V. over 30 minutes | ↑ CI ≥15% | 1. ΔVpeak_aor_ = 12% |
| Michard, 2000 ^(69)^ | 40 (40) | ICU | 1. Sepsis  2. Acute circulatory failure  3. Hemodynamic stability | 1. Arrhythmias  2. Severe hypoxemia  3. Pulmonary artery occlusion pressure > 18 mmHg | 1. V_T_ of 8 - 12 ml/kg  2. Mean PEEP of 7 cmH_2_O  3. Volume-controlled mode | 500 ml of 6% HES I.V. over 20 minutes | ↑ CI ≥15% | 1. PPV =13% |

**Table AF 1.** ΔABF_PLR_ = aortic blood flow variation induced by passive leg raising. ARDS = acute respiratory distress syndrome. ASA = American society of anesthesiologists. BMI = body mass index. BNP = B-type natriuretic peptide. CI = cardiac index. ΔCI = cardiac index variation. ΔCI_100_ = ΔCI induced by 100ml of volume expansion. ΔCI_EEO_ = ΔCI induced by end-expiratory occlusion. ΔCI_EEOcrs>30_ = ΔCI_EEO_ with compliance of the respiratory system higher than 30. ΔCI_EEOcrs≤30_ = ΔCI_EEO_ with compliance of the respiratory system lower or equal than 30. ΔCI_EEO+EIO_ =ΔCI induced by end-expiratory occlusion and end-inspiratory occlusion. ∆CI_EIO_ = ΔCI induced by end-inspiratory occlusion. ΔCI_PLR_ = ΔCI induced by passive leg raising. ΔCI_PLRcrs>30_ = ∆CI_PLR_ with compliance of the respiratory system higher than 30. ΔCI_PLRcrs≤30_ = ∆CI_PLR_ with compliance of the respiratory system lower or equal than 30. ΔCI_TREND_ = ΔCI induced by trendelenburg maneuver. CO = cardiac output. ΔCO = cardiac output variation. ΔCO_PLR_ = ΔCO induced by passive leg raising. COPD = chronic obstructive pulmonary disease. CRT = capillary refill time. CVP = central venous pressure. DP = driving pressure (calculated as the difference between plateau pressure and PEEP). ECMO = extracorporeal membrane oxygenation. Ecw = chest wall elastance. ED = emergency department. Ers = total respiratory system elastance. ΔEtCO_2_ = end-tidal carbon dioxide variation. ΔEtCO_2PLR_ = ΔEtCO_2_ induced by passive leg raising. FTc = corrected flow time. GEDVI = global end diastolic volume index. HES = hydroxyethyl starch. HR: heart rate. IABP = intra-aortic balloon pump. IBW = ideal body weight. ICU = intensive care unit. IDVGI = initial distribution volume of glucose index. ΔIJV = internal jugular vein variation. I.V. = intravenous. ITBVI = intrathoracic blood volume index. ΔIVC = inferior vena cava variation. ΔIVC_VT6_ = ΔIVC with tidal volume of 6 ml/kg. ΔIVC_VT9_ = ΔIVC with tidal volume of 9 ml/kg. ΔIVC_VT12_ = ΔIVC with tidal volume of 12 ml/kg. LVETc = left ventricular ejection time corrected for heart rate. MAP = mean arterial pressure. N = number of volume expansion and (number of patient). OpR = operating room. PBW = predicted body weight. PCWP = pulmonary capillary wedge pressure. PEEP = positive end-expiratory pressure. ∆PEP_KT_ = pre-ejection period of the radial arterial pressure curve variation. ∆PEP_PLET_ = pre-ejection period pulse of the plethysmographic curve variation. PI = perfusion index. PI_EAR_ = PI measured at the ear. PI_FOREHEAD_ = PI measured at the forehead. ΔPOP = pulse oximetry plethysmographic waveform amplitude variation. PPV = pulse pressure variation. PPV_7ML/KG_ = PPV induced by 7ml/kg volume expansion. PPV_100_ = PPV induced by 100-mL volume expansion. PPVa = PPV with automated measurement. PPV_crs>30_ = PPV with compliance of the respiratory system higher than 30. PPV_crs≤30_ = PPV with compliance of the respiratory system lower or equal than 30. PPV_EEO_ = PPV induced by end-expiratory occlusion. PPV_LRM_ = PPV induced by lung recruitment maneuvers. PPVni = PPV non-invasive. PPV_PLR_ = PPV induced by passive leg raising. PPV_VT6_ = PPV with tidal volume of 6 ml/kg. PPV_VT6-8_ = PPV induced by increasing the V_T_ from 6 ml/kg to 8 ml/kg. PPV_VT8_ = PPV with tidal volume of 8 ml/kg. PPV_VT9_ = PPV with tidal volume of 9 ml/kg. PPV_VT12_ = PPV with tidal volume of 12 ml/kg. Ppl = pleural pressure. PVI = plethysmographic variability index. PVI_EAR_ = PVI measured at the ear. PVI_FOREHEAD_ = PVI measured at the forehead. RR = respiratory rate. RSVT = automated respiratory systolic variation test. SBP = systolic blood pressure. ΔSBP = systolic blood pressure variation. ΔSBP_EEO_ = ΔSBP induced by end-expiratory occlusion. SV = stroke volume. SVV = stroke volume variation. SVV_7ML/KG_ = SVV induced by 7ml/kg volume expansion. SVV_EEO_ = SVV induced by end-expiratory occlusion. ΔSVC = superior vena cava variation. SVI = stroke volume index. SVVI = stroke volume index variation. SVVI_50_ = SVVI induced by 50-ml volume expansion. SVVI_100_= SVVI induced by 100-ml volume expansion. SVV_LRM_ = SVV induced by lung recruitment maneuvers. SVVI_PLR_ = SVVI induced by passive leg raising. SVV_VT6-8_ = SVV induced by increasing the V_T_ from 6 ml/kg to 8 ml/kg. SVV_VT8_ = SVV with tidal volume of 8 ml/kg. UO = urine output. ΔVpeak = peak velocity variation. ∆Vpeak_aor_ = ΔVpeak of aorta artery. ΔVpeak_brac_ = ΔVpeak of brachial artery. ∆Vpeak_carot_ = ΔVpeak of carotid artery. ΔVpeak_EEO_ = ΔVpeak induced by end-expiratory occlusion. V_T_ = tidal volume. VTI = velocity time integral. ∆VTI = velocity time integral variation. ∆VTI_EEO_ = ∆VTI induced by end-expiratory occlusion. ∆VTI_EIO_ = ∆VTI induced by end-inspiratory occlusion. ∆VTI_EEO+EIO_ = ∆VTI induced by end-expiratory occlusion test and end-inspiratory occlusion test.

**Table AF 2.** The Quality Assessment of Diagnostic Accuracy Studies tool (QUADAS) of each individual study.

| The QUADAS tool | | | | | | | | | | | | | | | | |
| --- | --- | --- | --- | --- | --- | --- | --- | --- | --- | --- | --- | --- | --- | --- | --- | --- |
| **Studies** | **1** | **2** | **3** | **4** | **5** | **6** | **7** | **8** | **9** | **10** | **11** | **12** | **13** | **14** | Quality |  |
| De Courson, 2020 ^(1)^ | Y | Y | Y | Y | Y | Y | N | Y | Y | N | N | Y | Y | Y | High |  |
| Wang, 2020 ^(2)^ | N | Y | Y | Y | Y | Y | Y | Y | Y | N | N | Y | Y | Y | High |  |
| Weil, 2020 ^(3)^ | Y | Y | Y | Y | Y | Y | Y | Y | Y | N | N | Y | Y | Y | High |  |
| He, 2019 ^(4)^ | Y | Y | Y | Y | Y | Y | Y | N | Y | Y | Y | Y | N | Y | High |  |
| Messina, 2019 ^(5)^ | N | Y | Y | Y | Y | Y | N | Y | Y | N | N | Y | Y | Y | High |  |
| Georges, 2018 ^(6)^ | Y | Y | Y | Y | Y | Y | N | Y | Y | Y | Y | Y | Y | N | High |  |
| Giraud, 2018 ^(7)^ | N | Y | Y | Y | Y | Y | Y | Y | Y | N | N | Y | Y | N | High |  |
| Le Guen, 2018 ^(8)^ | Y | Y | Y | Y | Y | Y | Y | Y | N | Y | Y | Y | Y | Y | High |  |
| Wang, 2018 ^(9)^ | N | Y | Y | Y | Y | Y | Y | Y | Y | N | N | Y | Y | Y | High |  |
| Biais, 2017 ^(10)^ | N | Y | Y | Y | Y | Y | N | Y | Y | N | N | Y | N | Y | Low |  |
| Biais, 2017 ^(11)^ | N | Y | Y | Y | Y | Y | N | Y | Y | N | N | Y | N | N | Low |  |
| Biais, 2017 ^(12)^ | Y | Y | Y | Y | Y | Y | N | Y | Y | N | N | Y | N | N | Low |  |
| Jozwiak, 2017 ^(13)^ | N | Y | Y | Y | Y | Y | N | Y | Y | Y | Y | Y | Y | Y | High |  |
| Lu, 2017 ^(14)^ | N | Y | Y | Y | Y | Y | Y | Y | Y | N | N | Y | Y | Y | High |  |
| Myatra, 2017 ^(15)^ | N | Y | Y | Y | Y | Y | N | Y | Y | N | N | Y | Y | Y | High |  |
| Yonis, 2017 ^(16)^ | N | Y | Y | Y | Y | Y | N | Y | Y | N | N | Y | Y | Y | High |  |
| De Broca, 2016 ^(17)^ | Y | Y | Y | Y | Y | Y | N | Y | Y | N | N | Y | Y | Y | High |  |
| Lee, 2016 ^(18)^ | N | Y | Y | Y | Y | Y | N | Y | Y | N | N | Y | Y | Y | High |  |
| Liu, 2016 ^(19)^ | Y | Y | Y | Y | Y | Y | Y | Y | Y | Y | Y | Y | Y | Y | High |  |
| Wu, 2016 ^(20)^ | Y | Y | Y | Y | Y | Y | N | Y | Y | N | N | Y | Y | Y | High |  |
| Angappan, 2015 ^(21)^ | N | Y | Y | Y | Y | Y | N | Y | Y | N | N | Y | Y | Y | High |  |
| Ibarra-Estrada, 2015 ^(22)^ | Y | Y | Y | Y | Y | Y | N | Y | Y | Y | Y | Y | Y | N | High |  |
| Mallat, 2015 ^(23)^ | N | Y | Y | Y | Y | Y | N | Y | Y | N | N | Y | Y | N | Low |  |
| Charbonneau, 2014 ^(24)^ | N | Y | Y | Y | Y | Y | Y | Y | Y | Y | Y | Y | Y | N | High |  |
| Guarracino, 2014 ^(25)^ | Y | Y | Y | Y | Y | Y | Y | Y | Y | Y | Y | Y | Y | N | High |  |
| Siswojo 2014 ^(26)^ | N | Y | Y | Y | Y | Y | Y | Y | N | N | N | Y | Y | Y | High |  |
| Feissel, 2013 ^(27)^ | N | Y | Y | Y | Y | Y | Y | Y | Y | N | N | Y | Y | Y | High |  |
| Fischer, 2013 ^(28)^ | Y | Y | Y | Y | Y | Y | Y | Y | Y | Y | Y | Y | Y | Y | High |  |
| Freitas, 2013 ^(29)^ | N | Y | Y | Y | Y | Y | Y | Y | Y | N | N | Y | Y | Y | High |  |
| Ishihara, 2013 ^(30)^ | N | Y | Y | Y | Y | Y | N | Y | Y | N | N | Y | Y | Y | High |  |
| Monnet, 2013 ^(31)^ | N | Y | Y | Y | Y | Y | N | Y | Y | N | N | Y | Y | N | Low |  |
| Monnet, 2013 ^(32)^ | Y | Y | Y | Y | Y | Y | N | Y | Y | N | N | Y | Y | Y | High |  |
| Trepte, 2013 ^(33)^ | N | Y | Y | Y | Y | Y | Y | Y | Y | N | N | Y | Y | Y | High |  |
| Vos, 2013 ^(34)^ | N | N | Y | Y | Y | Y | N | Y | Y | N | N | Y | Y | N | Low |  |
| Biais, 2012 ^(35)^ | N | Y | Y | Y | Y | Y | N | Y | Y | Y | Y | Y | Y | Y | High |  |
| Cecconi, 2012 ^(36)^ | N | Y | Y | Y | Y | Y | Y | N | N | N | N | Y | Y | Y | Low |  |
| Fu, 2012 ^(37)^ | Y | Y | Y | Y | Y | Y | N | N | Y | N | N | Y | Y | Y | High |  |
| Haas, 2012 ^(38)^ | N | Y | Y | Y | Y | Y | Y | Y | Y | N | N | Y | Y | N | High |  |
| Khwannimit, 2012 ^(39)^ | N | Y | Y | Y | Y | N | Y | Y | Y | N | N | Y | Y | N | Low |  |
| Monge García, 2012 ^(40)^ | N | Y | Y | Y | Y | Y | N | Y | Y | N | N | Y | Y | Y | High |  |
| Monnet, 2012 ^(41)^ | Y | Y | Y | Y | Y | Y | N | Y | Y | N | N | Y | Y | Y | High |  |
| Monnet, 2012 ^(42)^ | N | Y | Y | Y | Y | Y | N | Y | Y | Y | Y | Y | Y | Y | High |  |
| Oliveira-Costa, 2012 ^(43)^ | N | Y | Y | Y | Y | Y | Y | Y | Y | N | N | Y | Y | Y | High |  |
| Desgranges, 2011 ^(44)^ | N | Y | Y | Y | Y | Y | Y | Y | Y | Y | Y | Y | Y | Y | High |  |
| Hood, 2011 ^(45)^ | N | Y | Y | Y | Y | Y | Y | Y | N | N | N | Y | N | N | Low |  |
| Lakhal, 2011 ^(46)^ | Y | Y | Y | Y | Y | N | Y | Y | Y | Y | Y | Y | Y | Y | High |  |
| Loupec, 2011 ^(47)^ | N | Y | Y | Y | Y | Y | Y | Y | Y | Y | Y | Y | Y | Y | High |  |
| Machare-Delgado, 2011 ^(48)^ | N | Y | Y | Y | Y | Y | N | Y | Y | Y | Y | Y | N | Y | High |  |
| Moretti, 2010 ^(49)^ | N | Y | Y | Y | Y | Y | Y | Y | Y | Y | Y | Y | Y | Y | High |  |
| Muller, 2010 ^(50)^ | Y | Y | Y | Y | Y | Y | Y | Y | Y | N | N | Y | Y | Y | High |  |
| Zimmermann, 2010 ^(51)^ | N | Y | Y | Y | Y | Y | N | Y | Y | N | N | Y | Y | N | Low |  |
| Monge García, 2009 ^(52)^ | N | Y | Y | Y | Y | Y | N | Y | Y | Y | Y | Y | Y | Y | High |  |
| Monnet, 2009 ^(53)^ | N | Y | Y | Y | Y | Y | N | Y | Y | N | N | Y | Y | N | Low |  |
| Muller, 2009 ^(54)^ | N | Y | Y | Y | Y | Y | Y | Y | Y | Y | U | Y | Y | U | High |  |
| Vallée, 2009 ^(55)^ | Y | Y | Y | Y | Y | Y | Y | Y | Y | N | N | Y | Y | Y | High |  |
| Biais, 2008 ^(56)^ | N | Y | Y | Y | Y | Y | N | Y | Y | Y | Y | Y | Y | Y | High |  |
| Cannesson, 2008 ^(57)^ | N | Y | Y | Y | Y | Y | N | Y | Y | Y | Y | Y | Y | Y | High |  |
| Huang, 2008 ^(58)^ | Y | N | Y | Y | Y | Y | Y | Y | Y | N | N | Y | Y | Y | High |  |
| Muller, 2008 ^(59)^ | N | Y | Y | Y | Y | Y | Y | Y | Y | N | N | Y | Y | Y | High |  |
| Feissel, 2007 ^(60)^ | N | Y | Y | Y | Y | Y | Y | Y | Y | Y | Y | Y | Y | Y | High |  |
| Lafanechère, 2006 ^(61)^ | N | Y | Y | U | Y | Y | N | Y | Y | N | N | Y | Y | Y | Low |  |
| De Backer, 2005 ^(62)^ | Y | Y | Y | Y | Y | Y | Y | Y | Y | N | N | Y | Y | Y | High |  |
| Feissel, 2005 ^(63)^ | N | Y | Y | Y | Y | Y | Y | Y | Y | Y | Y | Y | N | N | High |  |
| Monnet, 2005 ^(64)^ | N | Y | Y | Y | Y | Y | N | Y | Y | N | N | Y | Y | Y | High |  |
| Barbier, 2004 ^(65)^ | N | Y | Y | Y | Y | Y | Y | Y | Y | N | N | Y | Y | N | High |  |
| Feissel, 2004 ^(66)^ | N | Y | Y | Y | Y | Y | Y | Y | Y | N | N | Y | N | N | Low |  |
| Vieillard-Baron, 2004 ^(67)^ | Y | Y | Y | Y | Y | Y | Y | Y | Y | N | N | Y | N | Y | High |  |
| Feissel, 2001 ^(68)^ | N | Y | Y | Y | Y | Y | Y | Y | Y | N | N | Y | Y | N | High |  |
| Michard, 2000 ^(69)^ | N | Y | Y | Y | Y | Y | Y | Y | Y | N | N | Y | N | N | Low |  |

**Table AF 2.** N = No. U = Unclear. Y = Yes.

The author's subjective categorized studies as either high or low quality.

1. Was the spectrum of patients representative of the patients who will receive the test in practice?

2. Were selection criteria clearly described?

3. Is the reference standard likely to correctly classify the target condition?

4. Is the time period between reference standard and index test short enough to be reasonably sure that the target condition did not change between the two tests?

5. Did the whole sample or a random selection of the sample, receive verification using a reference standard of diagnosis?

6. Did patients receive the same reference standard regardless of the index test result?

7. Was the reference standard independent of the index test (i.e. the index test did not form part of the reference standard)?

8. Was the execution of the index test described in sufficient detail to permit replication of the test?

9. Was the execution of the reference standard described in sufficient detail to permit its replication?

10. Were the index test results interpreted without knowledge of the results of the reference standard?

11. Were the reference standard results interpreted without knowledge of the results of the index test?

12. Were the same clinical data available when test results were interpreted as would be available when the test is used in practice?

13. Were uninterpretable/ intermediate test results reported?

14. Were withdrawals from the study explained?

**Table AF 3**. Bayesian approach to summary the performance of maneuvers to predict fluid responsiveness in mechanically ventilated patients.

| **Maneuver** | **N^o^ of studies** | **N^o^ of patients** | **N^o^ of fluid challenges (responders /**  **non-responders)** | **Range**  **Threshold** | **Mean (SD)**  **Threshold** | **Sensitivity**  **(95%CI)** | **Specificity (95%CI)** | **References** |
| --- | --- | --- | --- | --- | --- | --- | --- | --- |
| PPV | 40 | 1,936 | 2,318  (1,118 / 1,200) | 4 to 25.8% | 11.5 (3.3) % | 75 (67-86) | 82 (76-90) | 22-26, 32, 33, 36, 37, 39-41, 43, 46, 49-51, 53-57, 60, 62-65, 67, 68, 71, 73, 76, 78, 79, 81-84, 88, 90 |
| SVV | 24 | 1,043 | 1,305  (614 / 691) | 8 to 24.8% | 12.1 (3.3) % | 77 (71-86) | 78 (72-87) | 22, 23, 26, 35, 36, 38, 39, 41-43, 51, 53-60, 63, 69, 72, 73, 77 |
| PVI | 17 | 603 | 671  (382 / 289) | 9.5 to 20% | 13.8 (3.1) % | 77 (69-89) | 77 (70-88) | 29, 35, 39, 41, 47, 48, 49, 53, 55, 58, 59, 65, 66, 68, 72, 78, 81 |
| CVP | 12 | 429 | 429  (264 / 165) | 6.5 to 12.5 mmHg | 9.0 (2.1) mmHg | 63 (50-75) | 68 (57-81) | 30, 35, 46, 51, 58, 65, 71, 72, 75, 78, 80, 86 |
| ∆IVC | 8 | 303 | 303  (152 / 151) | 11.1 to 21% | 15.4 (3.5) % | 66 (55-81) | 77 (69-89) | 25, 30, 35, 45, 69, 70, 86, 87 |

Note: The reported threshold value point represents the mean value of the maneuver. Sensitivity and specificity are reported along with their respective 95% confidence interval and expressed as percentages. Abbreviations: CVP = central venous pressure. ∆IVC = inferior vena cava variation. N^o^ = number. PPV = pulse pressure variation. PVI = plethysmographic variability index. SVV = stroke volume variation.

**Table AF 4**. Individual data of each maneuver to assess fluid responsiveness in mechanically ventilated patients.

| **Author, year** | **Maneuver** | **Sens (%)** | **Spec (%)** | **PV + (%)** | **PV - (%)** | **LR +** | **LR -** | **Accuracy (%)** | **Youden index** | **DOR** | **AUC**  **(95%CI) or ± SD** |
| --- | --- | --- | --- | --- | --- | --- | --- | --- | --- | --- | --- |
| De Courson, 2020 ^(1)^ | ∆EtCO_2_ = 1.1% | 63 | 78 | 51 | 85 | 2.98 | 0.47 | 74 | 0.41 | 6.04 | 0.68 (0.68-0.69) |
|  | PPV = 9.5% | 79 | 51 | 37 | 87 | 1.63 | 0.41 | 58 | 0.28 | 3.77 | 0.64 (0.64-0.64) |
|  | SVV = 12.5% | 73 | 54 | 37 | 86 | 1.71 | 0.75 | 60 | 0.25 | 3.35 | 0.65 (0.65-0.65) |
| Wang, 2020 ^(2)^ | ∆VTI = 15.9% | 88 | 95 | 95 | 86 | 17.50 | 0.13 | 91 | 0.83 | 133.00 | 0.96 (0.90-1.00) |
|  | SVV = 24.8% | 67 | 95 | 94 | 70 | 13.33 | 0.35 | 80 | 0.62 | 38.00 | 0.80 (0.66-0.94) |
|  | PPV = 25.8% | 83 | 75 | 80 | 79 | 3.33 | 0.22 | 80 | 0.58 | 15.00 | 0.84 (0.73-0.96) |
| Weil, 2020 ^(3)^ | PPV = 14% | 68 | 81 | 70 | 81 | 3.72 | 0.39 | 77 | 0.49 | 9.56 | 0.78 (0.64-0.89) |
| He, 2019 ^(4)^ | PPV_VT6_ = 8.5% | 55 | 83 | 75 | 67 | 3.24 | 0.54 | 70 | 0.38 | 6.00 | 0.79 (0.70-0.89) |
|  | ∆IVC_VT6_ = 11.1% | 68 | 76 | 72 | 72 | 2.81 | 0.42 | 72 | 0.44 | 6.72 | 0.71 (0.60-0.83) |
|  | PPV_VT9_ = 12.5% | 76 | 93 | 91 | 81 | 10.43 | 0.26 | 85 | 0.69 | 40.81 | 0.91 (0.85-0.98) |
|  | ∆IVC_VT9_ = 15.3% | 55 | 88 | 81 | 68 | 4.53 | 0.51 | 72 | 0.43 | 8.89 | 0.79 (0.70-0.89) |
|  | PPV_VT12_ = 15.5% | 87 | 90 | 89 | 88 | 8.90 | 0.15 | 89 | 0.77 | 61.05 | 0.93 (0.88-0.99) |
|  | ∆IVC_VT12_ = 13.4% | 53 | 88 | 80 | 67 | 4.32 | 0.54 | 71 | 0.41 | 8.00 | 0.73 (0.62-0.84) |
| Messina, 2019 ^(5)^ | PPV_VT6-8_ = 12.2% | 95 | 95 | 95 | 95 | 19.89 | 0.06 | 95 | 0.92 | 360.00 | 0.96 (0.87-1.00) |
|  | SVV_VT6-8_ = 8.0% | 95 | 95 | 95 | 95 | 19.89 | 0.06 | 95 | 0.92 | 360.00 | 0.96 (0.89-1.00) |
| Georges, 2018 ^(6)^ | ∆VTI_EEO_ = 9% | 89 | 95 | 96 | 88 | 19.64 | 0.11 | 92 | 0.85 | 175.00 | 0.96 ± 0.03 |
|  | ΔVpeak_EEO_ = 8.5% | 64 | 77 | 78 | 63 | 2.83 | 0.46 | 70 | 0.42 | 6.12 | 0.70 ± 0.07 |
| Giraud, 2018 ^(7)^ | ΔSVC = 14.3% | 100 | 91 | 90 | 1.00 | 11.00 | 0.00 | 95 | 0.91 | 180.00 | 0.97 (0.90-1.00) |
| Le Guen, 2018 ^(8)^ | PVI = 10.5% | 59 | 47 | 52 | 53 | 1.10 | 0.89 | 53 | 0.06 | 1.24 | 0.49 (0.36-0.62) |
| Wang, 2018 ^(9)^ | ΔIVC = 15% | 82 | 86 | 89 | 78 | 6.40 | 0.23 | 83 | 0.68 | 28.00 | 0.86 |
|  | CVP = 11mmHg | 33 | 100 | 100 | 53 | --- | 0.70 | 61 | 0.33 | 6.86 | 0.60 |
|  | GEDVI = 517 ml/m^2^ | 89 | 46 | 69 | 80 | 1.80 | 0.20 | 72 | 0.35 | 9.00 | 0.63 |
|  | ITBVI = 608 ml/m^2^ | 100 | 27 | 63 | 100 | 1.33 | 0.00 | 67 | 0.27 | 6.67 | 0.54 |
| Biais, 2017 ^(10)^ | SVV_LRM_ = 30% | 88 | 92 | 93 | 85 | 10.50 | 0.14 | 89 | 0.80 | 77.00 | 0.96 (0.81-0.99) |
|  | PPV_LRM_ = 6% | 69 | 75 | 79 | 64 | 2.75 | 0.42 | 71 | 0.44 | 6.60 | 0.72 (0.52-0.88) |
| Biais, 2017 ^(11)^ | SVV_EEO_ = 5% | 100 | 81 | 84 | 100 | 5.25 | 0.00 | 90 | 0.81 | 170.00 | 0.91 (0.81-1.00) |
|  | PPV_EEO_ = 1% | 55 | 71 | 65 | 61 | 1.93 | 0.63 | 63 | 0.26 | 3.06 | 0.62 (0.44-0.80) |
|  | PPV = 9% | 60 | 86 | 81 | 68 | 4.20 | 0.47 | 73 | 0.46 | 9.00 | 0.75 (0.60-0.90) |
| Biais, 2017 ^(12)^ | SVVI_50_ = 2% | 89 | 67 | 56 | 93 | 2.68 | 0.16 | 74 | 0.56 | 16.67 | 0.83 (0.75-0.92) |
|  | SVVI_100_ = 6% | 93 | 85 | 74 | 96 | 6.19 | 0.08 | 88 | 0.78 | 73.67 | 0.95 (0.90-0.99) |
|  | PPV = 10% | 54 | 68 | 44 | 76 | 1.69 | 0.68 | 64 | 0.22 | 2.49 | 0.65 (0.53-0.78) |
| Jozwiak, 2017 ^(13)^ | ∆CI_EEO_ = 4% | 93 | 100 | 100 | 94 | --- | 0.07 | 97 | 0.93 | 420.00 | 0.98 (0.85-1.00) |
|  | ∆CI_EIO_ = -10% | 60 | 93 | 90 | 70 | 9.00 | 0.43 | 77 | 0.53 | 21.00 | 0.76 (0.57-0.90) |
|  | ∆CI_EEO+EIO_ = 11% | 93 | 80 | 82 | 92 | 4.67 | 0.08 | 87 | 0.73 | 56.00 | 0.92 (0.77-0.99) |
|  | ∆VTI_EEO_ = 5% | 93 | 100 | 100 | 94 | --- | 0.07 | 97 | 0.93 | 420.00 | 0.94 (0.79-0.99) |
|  | ∆VTI_EIOT_ = -8% | 80 | 87 | 86 | 81 | 6.00 | 0.23 | 83 | 0.67 | 26.00 | 0.90 (0.74-0.98) |
|  | ∆VTI_EEO+EIO_ = 13% | 93 | 93 | 93 | 93 | 14.00 | 0.07 | 93 | 0.86 | 196.00 | 0.97 (0.84-1.00) |
| Lu, 2017 ^(14)^ | CVP = 6.5 mmHg | 65 | 70 | 72 | 63 | 2.10 | 0.49 | 67 | 0.35 | 4.29 | 0.68 (0.51-0.84) |
|  | ITBVI = 871 ml/m^2^ | 55 | 65 | 65 | 54 | 1.53 | 0.70 | 59 | 0.20 | 2.19 | 0.66 (0.49-0.84) |
|  | SVV = 11.5% | 75 | 85 | 87 | 73 | 5.43 | 0.30 | 80 | 0.60 | 18.10 | 0.85 (0.73-0.97) |
|  | PVI = 15.5% | 65 | 80 | 82 | 67 | 3.67 | 0.41 | 73 | 0.45 | 9.00 | 0.82 (0.69-0.95) |
|  | ∆IVC = 20.5% | 67 | 77 | 78 | 65 | 2.93 | 0.43 | 71 | 0.44 | 6.80 | 0.81 (0.67-0.94) |
|  | ∆Vpeak_carot_ = 13.0% | 78 | 90 | 91 | 77 | 8.56 | 0.24 | 84 | 0.68 | 35.00 | 0.91 (0.82-1.00) |
|  | ∆Vpeak_brac_ = 11.7% | 70 | 80 | 83 | 69 | 3.87 | 0.36 | 76 | 0.50 | 10.69 | 0.76 (0.60-0.92) |
| Myatra, 2017 ^(15)^ | PPV_VT8_ = 11.5% | 75 | 100 | 100 | 78 | --- | 0.25 | 87 | 0.75 | 84.00 | 0.91 (0.81-1.00) |
|  | SVV_VT8_ = 10.5% | 75 | 93 | 92 | 76 | 10.50 | 0.27 | 83 | 0.68 | 39.00 | 0.92 (0.82-1.00) |
|  | PPV_VT6-8_ = 3.5% | 94 | 100 | 100 | 93 | --- | 0.06 | 97 | 0.94 | 420.00 | 0.99 (0.98-1.00) |
|  | SVV_VT6-8_ = 2.5% | 88 | 100 | 100 | 88 | --- | 0.13 | 93 | 0.88 | 196.00 | 0.97 (0.92-1.00) |
|  | PPV_7ML/KG_ = 1.5% | 94 | 100 | 100 | 91 | --- | 0.06 | 97 | 0.94 | 420.00 | 0.98 (0.95-1.00) |
|  | SVV_7ML/KG_ = 2.5% | 75 | 71 | 92 | 60 | 2.63 | 0.35 | 73 | 0.46 | 7.50 | 0.71 (0.52-0.92) |
| Yonis, 2017 ^(16)^ | ΔCI_TREND_ = 8% | 87 | 89 | 0.87 | 0.89 | 7.90 | 0.15 | 88 | 0.76 | 52.00 | 0.90 (0.80-1.00) |
|  | PPV_VT6_ = 10% | 33 | 80 | 0.60 | 0.57 | 1.65 | 0.84 | 58 | 0.13 | 2.00 | 0.49 (0.21-0.77) |
|  | PPV_VT8_ = 9% | 78 | 40 | 0.54 | 0.67 | 1.30 | 0.56 | 58 | 0.18 | 2.34 | 0.52 (0.24-0.80) |
|  | PPV_VT6-8_ = 29% | 100 | 40 | 0.60 | 1.00 | 1.67 | 0.00 | 68 | 0.40 | 12.00 | 0.59 (0.31-0.88) |
|  | ΔCI_EEO_ = 10% | 33 | 100 | 100 | 0.64 | --- | 0.67 | 70 | 0.33 | 18.00 | 0.65 (0.46-0.84) |
| De Broca, 2016 ^(17)^ | SVV_LRM_ = 16% | 92 | 96 | 100 | 89 | 21.14 | 0.08 | 93 | 0.92 | 249.33 | 0.95 (0.91-0.99) |
|  | PPV_LRM_ = 25% | 62 | 96 | 96 | 61 | 14.30 | 0.40 | 75 | 0.62 | 36.14 | 0.81 (0.70-0.91) |
|  | SVV = 8% | 71 | 71 | 81 | 60 | 2.40 | 0.39 | 72 | 0.43 | 6.17 | 0.80 (0.70-0.94) |
| Lee, 2016 ^(18)^ | SVV = 12% | 92 | 57 | 80 | 80 | 2.15 | 0.13 | 80 | 0.49 | 16.00 | 0.83 (0.65-0.94) |
|  | PPV = 15% | 86 | 85 | 92 | 75 | 5.92 | 0.18 | 85 | 0.71 | 33.00 | 0.81 (0.77-1.00) |
|  | PVI = 10% | 80 | 70 | 84 | 67 | 2.83 | 0.27 | 78 | 0.50 | 10.50 | 0.79 (0.56-0.92) |
| Liu, 2016 ^(19)^ | PPV = 10% | 67 | 84 | 83 | 69 | 4.20 | 0.40 | 75 | 0.51 | 10.88 | 0.78 (0.69-0.86) |
|  | PPV/DP = 0.86% | 42 | 98 | 96 | 59 | 18.62 | 0.59 | 68 | 0.40 | 31.53 | 0.73 (0.60-0.81) |
|  | PPV/VT = 1.15% | 89 | 68 | 77 | 83 | 2.80 | 0.17 | 79 | 0.57 | 16.43 | 0.81 (0.71-0.88) |
|  | PPV X Ers = 300% | 75 | 75 | 78 | 72 | 3.00 | 0.33 | 75 | 0.50 | 9.00 | 0.80 (0.70-0.87) |
|  | PPV/(HR/RR) = 2.12% | 73 | 68 | 73 | 68 | 2.40 | 0.40 | 71 | 0.41 | 5.82 | 0.75 (0.65-0.84) |
|  | PPV = 12% | 42 | 89 | 82 | 57 | 3.72 | 0.65 | 64 | 0.31 | 5.72 | 0.78 (0.69-0.86) |
|  | PPV/Ecw = 0.99% | 83 | 93 | 93 | 82 | 12.13 | 0.20 | 88 | 0.76 | 65.30 | 0.94 (0.87-0.97) |
|  | PPV/Ppl = 2% | 92 | 93 | 94 | 91 | 13.54 | 0.08 | 93 | 0.85 | 164.00 | 0.94 (0.88-0.98) |
| Wu, 2016 ^(20)^ | PPV = 10% | 78 | 80 | 69 | 86 | 3.82 | 0.27 | 79 | 0.58 | 13.95 | 0.79 (0.67-0.89) |
|  | SVV = 12% | 70 | 72 | 59 | 80 | 2.47 | 0.42 | 71 | 0.42 | 5.82 | 0.75 (0.63-0.85) |
|  | PVI = 11% | 96 | 59 | 58 | 96 | 2.33 | 0.07 | 73 | 0.55 | 31.63 | 0.80 (0.68-0.89) |
| Angappan, 2015 ^(21)^ | SVV = 13% | 78 | 89 | 92 | 70 | 6.34 | 0.24 | 82 | 0.67 | 26.83 | 0.72 (0.56-0.84) |
| Ibarra-Estrada, 2015 ^(22)^ | ∆Vpeak_carot_ = 14% | 86 | 86 | 86 | 85 | 6.28 | 0.15 | 86 | 0.72 | 40.63 | 0.88 (0.77-0.95) |
|  | SVV = 16% | 76 | 68 | 71 | 73 | 2.47 | 0.34 | 73 | 0.44 | 7.30 | 0.72 (0.59-0.83) |
|  | SVVI_PLR_ = 15% | 63 | 72 | 70 | 65 | 2.30 | 0.51 | 68 | 0.35 | 4.53 | 0.69 (0.56-0.80) |
|  | PPV = 14% | 50 | 79 | 71 | 60 | 2.42 | 0.63 | 64 | 0.29 | 3.83 | 0.63 (0.49-0.75) |
| Mallat, 2015 ^(23)^ | ΔCI_100_ = 5.2% | 77 | 74 | 71 | 80 | 2.98 | 0.31 | 76 | 0.51 | 9.71 | 0.78 (0.64-0.88) |
|  | SVV_100_ = -2% | 86 | 89 | 86 | 89 | 7.77 | 0.15 | 88 | 0.75 | 50.67 | 0.91 (0.80-0.97) |
|  | PPV_100_ = -2% | 86 | 85 | 83 | 88 | 5.83 | 0.16 | 86 | 0.71 | 36.42 | 0.92 (0.81-0.98) |
| Charbonneau, 2014 ^(24)^ | ΔSVC = 29% | 54 | 89 | 88 | 57 | 4.85 | 0.52 | 68 | 0.43 | 9.33 | 0.74(0.59-0.88) |
|  | ΔIVC = 21% | 38 | 61 | 59 | 41 | 0.99 | 1.00 | 48 | -0.01 | 0.98 | 0.43 (0.25-0.61) |
| Guarracino, 2014 ^(25)^ | PPV = 12.5% | 96 | 55 | 76 | 92 | 2.15 | 0.06 | 80 | 0.51 | 35.44 | 0.85 (0.72-0.94) |
|  | CVP = 8mmHg | 33 | 100 | 100 | 50 | --- | 0.67 | 60 | 0.33 | 20.00 | 0.68 (0.45-0.75) |
|  | ΔIJV = 18% | 80 | 95 | 96 | 76 | 16.00 | 0.21 | 86 | 0.75 | 76.00 | 0.92 (0.80-0.98) |
|  | ΔIJV = 9.9% and PPV = 12% | 100 | 95 | 97 | 100 | 20.00 | 0.00 | 98 | 0.95 | 1140.00 | --- |
| Siswojo, 2014 ^(26)^ | PVI = 10.5% | 88 | 67 | 79 | 80 | 2.65 | 0.18 | 79 | 0.55 | 15.00 | 0.84 (0.69-0.99) |
| Feissel, 2013 ^(27)^ | PVI = 19% | 94 | 87 | 88 | 93 | 7.03 | 0.07 | 90 | 0.81 | 97.50 | 0.97 (0.83-0.99) |
| Fischer, 2013 ^(28)^ | PPV = 14% | 64 | 78 | 88 | 47 | 2.91 | 0.47 | 68 | 0.42 | 6.17 | 0.73 (0.63-0.83) |
|  | PVI = 20% | 38 | 87 | 88 | 36 | 2.96 | 0.71 | 53 | 0.25 | 4.19 | 0.60 (0.48-0.71) |
| Freitas, 2013 ^(29)^ | PPV_VT6_ = 6.5% | 89 | 90 | 89 | 90 | 9.39 | 0.12 | 90 | 0.79 | 80.75 | 0.91 (0.82-1.00) |
|  | PPV_VT8_ = 12.3% | 80 | 95 | 93 | 95 | 16.58 | 0.22 | 88 | 0.75 | 75.00 | --- |
| Ishihara, 2013 ^(30)^ | SVV = 10.5 % | 45 | 81 | 70 | 60 | 2.38 | 0.65 | 63 | 0.26 | 3.64 | 0.61 |
|  | PPV = 8.5% | 50 | 71 | 64 | 59 | 1.74 | 0.68 | 61 | 0.21 | 2.57 | 0.65 |
|  | CVP = 6.5 mmHg | 60 | 74 | 70 | 65 | 2.43 | 0.52 | 67 | 0.35 | 4.67 | 0.69 |
|  | ITBVI = 0.78 L/m^2^ | 65 | 48 | 55 | 58 | 1.30 | 0.70 | 58 | 0.13 | 1.88 | 0.58 |
|  | IDVGI = 4.23 L/m^2^ | 85 | 57 | 66 | 79 | 1.93 | 0.24 | 72 | 0.42 | 8.15 | 0.67 |
| Monnet, 2013 ^(31)^ | ∆CI_PLR_ = 10% | 95 | 95 | 95 | 95 | 19.00 | 0.10 | 95 | 0.90 | 360.00 | 0.98 (0.88-1.00) |
|  | ∆EtCO_2PLR_ = 5% | 71 | 100 | 100 | 76 | 4.50 | 0.30 | 85 | 0.71 | 95.00 | 0.93 (0.81-0.99) |
| Monnet, 2013 ^(32)^ | PPV = 11% | 93 | 95 | 93 | 95 | 17.70 | 0.10 | 94 | 0.88 | 266.00 | 0.93 ± 0.06 |
|  | SVV = 10% | 93 | 90 | 88 | 95 | 9.33 | 0.10 | 91 | 0.83 | 126.00 | 0.89 ± 0.07 |
|  | PVI = 16% | 47 | 90 | 61 | 80 | 1.90 | 0.30 | 71 | 0.37 | 7.88 | 0.68 ± 0.09 |
| Trepte, 2013 ^(33)^ | PPV = 10.1% | 61 | 81 | 81 | 61 | 3.15 | 0.48 | 69 | 0.42 | 6.51 | 0.70 (0.21-0.85) |
|  | SVV = 9.9% | 63 | 74 | 76 | 61 | 2.46 | 0.49 | 68 | 0.38 | 4.98 | 0.72 (0.21-0.85) |
|  | RSVT = 19.7^0^ | 78 | 68 | 76 | 70 | 2.42 | 0.32 | 74 | 0.46 | 7.47 | 0.77 (0.55-0.80) |
| Vos, 2013 ^(34)^ | SVV = 15% | 70 | 92 | 92 | 71 | 9.18 | 0.32 | 80 | 0.62 | 28.80 | 0.81 (0.65-0.98) |
|  | PPV = 14% | 88 | 62 | 75 | 80 | 2.29 | 0.19 | 77 | 0.50 | 12.00 | 0.77 (0.59-0.94) |
|  | PVI = 12% | 82 | 77 | 82 | 77 | 3.57 | 0.23 | 80 | 0.59 | 15.56 | 0.78 (0.59-0.96) |
| Biais, 2012 ^(35)^ | SVV = 12.6% | 63 | 69 | 71 | 61 | 2.02 | 0.54 | 66 | 0.32 | 3.77 | 0.60 (0.43-0.76) |
|  | PPV = 10% | 89 | 88 | 89 | 88 | 7.16 | 0.12 | 89 | 0.77 | 59.50 | 0.95 (0.82-0.99) |
| Cecconi, 2012 ^(36)^ | ∆SBP = 9% | 73 | 76 | 50 | 90 | 2.85 | 0.34 | 74 | 0.49 | 8.40 | 0.70 (0.52-0.88) |
|  | PPV =13% | 83 | 74 | 53 | 93 | 3.17 | 0.23 | 77 | 0.57 | 14.00 | 0.87 (0.76-0.99) |
|  | SVV = 12.5% | 75 | 83 | 60 | 90 | 4.75 | 0.30 | 81 | 0.58 | 16.00 | 0.84 (0.71-0.96) |
| Fu, 2012 ^(37)^ | SVV = 12.5% | 88 | 83 | 90 | 81 | 5.81 | 0.15 | 86 | 0.71 | 38.25 | 0.86 (0.76-0.96) |
|  | PVI = 13.5% | 77 | 80 | 86 | 70 | 3.87 | 0.28 | 78 | 0.57 | 13.71 | 0.79 (0.65-0.92) |
|  | SVI = 43.5 ml/m^2^ | 83 | 91 | 93 | 78 | 8.39 | 0.18 | 86 | 0.74 | 46.80 | 0.73 (0.58-0.88) |
|  | CI = 2.85 L/min/m^2^ | 72 | 76 | 81 | 63 | 2.84 | 0.39 | 73 | 0.48 | 7.33 | 0.65 (0.49-0.81) |
|  | CVP = 7.5 mmHg | 61 | 64 | 73 | 52 | 1.75 | 0.60 | 63 | 0.25 | 2.94 | 0.61 (0.45-0.78) |
|  | MAP = 67.5 mmHg | 67 | 70 | 78 | 58 | 2.26 | 0.46 | 69 | 0.37 | 4.90 | 0.69 (0.52-0.78) |
|  | PI = 3.49% | 61 | 67 | 73 | 52 | 1.75 | 0.60 | 63 | 0.28 | 2.94 | 0.65 (0.49-0.81) |
| Haas, 2012 ^(38)^ | PVI = 16% | 100 | 89 | 67 | 100 | 9.00 | 0.00 | 91 | 0.89 | 64.00 | 0.95 |
|  | SVV = 11% | 100 | 72 | 44 | 100 | 3.60 | 0.00 | 77 | 0.72 | 20.80 | 0.87 |
| Khwannimit, 2012 ^(39)^ | SVV = 10% | 92 | 83 | 88 | 88 | 5.50 | 0.10 | 88 | 0.75 | 55.00 | 0.92 (0.83-1.00) |
|  | PPV = 12% | 83 | 83 | 87 | 79 | 5.00 | 0.20 | 83 | 0.66 | 25.00 | 0.92 (0.83-1.00) |
| Monge García, 2012 ^(40)^ | ∆CO_PLR_ = 12% | 95 | 94 | 95 | 94 | 15.24 | 0.05 | 95 | 0.89 | 300.00 | 0.97 (0.85-0.99) |
|  | ∆EtCO_2PLR_ = 5% | 91 | 94 | 95 | 88 | 14.48 | 0.10 | 92 | 0.85 | 142.50 | 0.94 (0.82-0.99) |
|  | FTc = 300ms | 67 | 81 | 82 | 65 | 3.56 | 0.41 | 73 | 0.48 | 8.67 | 0.75 (0.58-0.88) |
|  | PPV_PLR_ = 11% | 67 | 81 | 82 | 65 | 3.56 | 0.41 | 73 | 0.48 | 8.67 | 0.73 (0.55-0.86) |
| Monnet, 2012 ^(41)^ | PPV_crs>30_ = 12% | 85 | 100 | 100 | 84 | --- | 0.13 | 92 | 0.85 | 143.00 | 0.98 ± 0.03 |
|  | ∆CI_PLRcrs>30_ = 10% | 93 | 91 | 93 | 91 | 10.27 | 0.07 | 92 | 0.84 | 140.00 | 0.91 ± 0.06 |
|  | ∆CI_EEOcrs>30_ = 5% | 93 | 91 | 93 | 91 | 10.27 | 0.07 | 92 | 0.84 | 140.00 | 0.97 ± 0.03 |
|  | PPV_crs≤30_ = 4% | 100 | 31 | 64 | 100 | 1.44 | 0.00 | 68 | 0.31 | 13.33 | 0.69 ± 0.10 |
|  | ∆CI_PLRcrs≤30_ = 10% | 94 | 100 | 100 | 93 | --- | 0.07 | 96 | 0.94 | 364.00 | 0.94 ± 0.05 |
|  | ∆CI_EEO crs≤30_ = 5% | 93 | 92 | 93 | 92 | 12.13 | 0.07 | 93 | 0.85 | 168.00 | 0.93 ± 0.05 |
| Monnet, 2012 ^(42)^ | SVV = 14% | 76 | 82 | 76 | 82 | 4.21 | 0.29 | 79 | 0.58 | 14.63 | 0.84 (0.71-0.97) |
|  | PPV = 10% | 88 | 91 | 88 | 91 | 9.71 | 0.13 | 90 | 0.79 | 75.00 | 0.89 (0.77-1.00) |
|  | PPVni = 11% | 82 | 91 | 88 | 87 | 9.06 | 0.19 | 87 | 0.73 | 46.67 | 0.89 (0.78-1.00) |
|  | ∆CI_PLR_ = 11% | 100 | 91 | 89 | 100 | 11.00 | 0.00 | 95 | 0.91 | 340.00 | 0.95 (0.88-1.00) |
|  | ΔCI_EEO_ = 5% | 100 | 91 | 89 | 100 | 11.00 | 0.00 | 95 | 0.91 | 340.00 | 0.97 (0.91-1.00) |
| Oliveira-Costa, 2012 ^(43)^ | PPV = 10% | 53 | 95 | 90 | 70 | 9.40 | 0.34 | 76 | 0.48 | 21.38 | 0.74 (0.56-0.90) |
| Desgranges, 2011 ^(44)^ | PVI = 12% | 74 | 67 | 82 | 55 | 2.21 | 0.39 | 71 | 0.41 | 5.60 | 0.84 (0.69-0.99) |
|  | PVI_EAR_ = 16% | 74 | 89 | 93 | 62 | 6.63 | 0.30 | 79 | 0.63 | 22.40 | 0.88 (0.75-1.00) |
|  | PVI_FOREHEAD_ = 15% | 89 | 78 | 89 | 78 | 4.03 | 0.14 | 86 | 0.67 | 29.75 | 0.91 (0.78-1.00) |
|  | PI = 4.2% | 58 | 56 | 73 | 38 | 1.30 | 0.76 | 57 | 0.14 | 1.72 | 0.54 (0.29-0.78) |
|  | PI_EAR_ = 0.36 | 42 | 89 | 89 | 42 | 3.79 | 0.65 | 57 | 0.31 | 5.82 | 0.62 (0.41-0.83) |
|  | PI_FOREHEAD_ = 1.37% | 95 | 67 | 86 | 86 | 2.84 | 0.08 | 86 | 0.62 | 36.00 | 0.85 (0.70-1.00) |
|  | PPV = 11% | 74 | 89 | 93 | 62 | 6.63 | 0.30 | 79 | 0.63 | 22.40 | 0.84 (0.68-1.00) |
|  | CVP = 8mmHg | 58 | 33 | 65 | 27 | 0.87 | 1.26 | 50 | -0.09 | 0.69 | 0.42 (0.17-0.67) |
| Hood, 2011 ^(45)^ | PVI of 10% | 86 | 100 | 100 | 50 | --- | 0.14 | 88 | 0.86 | 38.00 | 0.96 (0.88-1.00) |
| Lakhal, 2011 ^(46)^ | PPV = 5% | 73 | 85 | 76 | 83 | 4.80 | 0.32 | 80 | 0.58 | 14.93 | 0.75 (0.62-0.85) |
| Loupec, 2011 ^(47)^ | PVI = 17% | 95 | 91 | 91 | 94 | 9.05 | 0.05 | 93 | 0.86 | 170.00 | 0.88 (0.74-0.96) |
|  | PPV = 10% | 100 | 95 | 95 | 100 | 19.00 | 0.00 | 98 | 0.95 | 756.00 | 0.97 (0.86-0.99) |
| Machare-Delgado, 2011 ^(48)^ | ΔIVC = 12% | 100 | 53 | 50 | 100 | 2.13 | 0.00 | 68 | 0.53 | 18.00 | 0.81 (0.64-0.99) |
|  | SVV = 12% | 50 | 65 | 40 | 73 | 1.42 | 0.77 | 60 | 0.15 | 1.83 | 0.57 (0.29-0.84) |
| Moretti, 2010 ^(49)^ | ∆IVC = 16% | 71 | 100 | 100 | 71 | --- | 0.29 | 83 | 0.71 | 57.60 | 0.90 (0.73-0.98) |
| Muller, 2010 ^(50)^ | PPV = 7% | 61 | 94 | 96 | 52 | 9.76 | 0.42 | 70 | 0.55 | 23.44 | 0.77 (0.65-0.90) |
|  | CVP = 9mmHg | 68 | 81 | 90 | 50 | 3.64 | 0.39 | 72 | 0.49 | 9.33 | 0.76 (0.64-0.89) |
| Zimmermann,  2010 ^(51)^ | SVV = 11% | 100 | 80 | 94 | 100 | 5.00 | 0.00 | 95 | 0.80 | 120.00 | 0.99 (0.97-1.00) |
|  | PVI = 9.5% | 93 | 100 | 100 | 83 | --- | 0.07 | 95 | 0.93 | 140.00 | 0.97 (0.91-1.00) |
|  | CVP = 10.5mmHg | 66 | 40 | 77 | 29 | 1.11 | 0.83 | 60 | 0.06 | 1.33 | 0.55 (0.27-0.83) |
| Monge García, 2009 ^(52)^ | ΔVpeak_brac_ = 10% | 74 | 95 | 93 | 78 | 14.00 | 0.28 | 84 | 0.69 | 50.40 | 0.88 (0.74-0.96) |
|  | PPV = 10% | 95 | 79 | 82 | 94 | 4.50 | 0.07 | 87 | 0.74 | 67.50 | 0.97 (0.86-0.99) |
|  | SVV = 11% | 95 | 89 | 90 | 94 | 9.00 | 0.06 | 92 | 0.84 | 153.00 | 0.89 (0.75-0.97) |
| Monnet, 2009 ^(53)^ | PPV_EEO_ = 5% | 87 | 100 | 100 | 79 | --- | 0.13 | 91 | 0.87 | 146.67 | 0.96 (0.83-0.99) |
|  | ∆CI_EEO_ = 5% | 91 | 100 | 100 | 85 | --- | 0.09 | 94 | 0.91 | 231.00 | 0.97 (0.85-1.00) |
|  | ∆SBP_EEO_ = 4% | 67 | 82 | 88 | 53 | 3.59 | 0.43 | 71 | 0.49 | 8.44 | 0.71 (0.53-0.86) |
|  | ∆CI_PLR_ = 10% | 91 | 100 | 100 | 85 | --- | 0.09 | 94 | 0.91 | 231.00 | 0.94 (0.80-0.99) |
|  | PPV_PLR_ = 11% | 48 | 91 | 92 | 45 | 5.26 | 0.57 | 62 | 0.39 | 9.17 | 0.68 (0.50-0.83) |
|  | CI = 2.8 L/min/m^2^ | 78 | 54 | 78 | 55 | 1.72 | 0.40 | 71 | 0.32 | 4.32 | 0.65 (0.47-0.80) |
| Muller, 2009 ^(54)^ | BNP = 193 pg/ml | 38 | 100 | 100 | 38 | --- | 0.63 | 55 | 0.38 | 10.80 | 0.74 ± 0.11 |
|  | CVP = 7 | 54 | 100 | 100 | 45 | --- | 0.46 | 67 | 0.54 | 21.27 | 0.77 ± 0.10 |
| Vallée, 2009 ^(55)^ | PPV = 15% | 60 | 71 | 64 | 67 | 2.04 | 0.57 | 65 | 0.31 | 3.54 | 0.72 (0.59-0.82) |
|  | PPV/DP = 0.9 | 67 | 84 | 79 | 75 | 4.28 | 0.39 | 76 | 0.51 | 10.86 | 0.81 (0.71-0.90) |
| Biais, 2008 ^(56)^ | SVV = 10% | 94 | 94 | 94 | 94 | 16.94 | 0.06 | 94 | 0.88 | 272.00 | 0.95 (0.81-0.99) |
| Cannesson, 2008 ^(57)^ | PPV = 12.5% | 87 | 89 | 93 | 80 | 7.88 | 0.14 | 88 | 0.76 | 56.00 | 0.94 (0.85-1.00) |
|  | ∆POP = 12% | 87 | 89 | 93 | 80 | 7.88 | 0.14 | 88 | 0.76 | 56.00 | 0.94 (0.86-1.00) |
|  | PPVa = 10.5% | 87 | 89 | 93 | 80 | 7.88 | 0.14 | 88 | 0.76 | 56.00 | 0.94 (0.85-1.00) |
|  | PVI = 14% | 81 | 100 | 100 | 75 | --- | 0.19 | 88 | 0.81 | 78.00 | 0.93 (0.83-1.00) |
|  | CVP = 12.5 mmHg | 44 | 78 | 78 | 44 | 1.97 | 0.72 | 56 | 0.22 | 2.72 | 0.42 (0.18-0.65) |
|  | PCWP = 14.5 mmHg | 50 | 67 | 73 | 43 | 1.50 | 0.75 | 56 | 0.17 | 2.00 | 0.40 (0.16-0.63) |
|  | CI = 2.8 L/min/m^2^ | 44 | 89 | 88 | 47 | 3.94 | 0.63 | 60 | 0.33 | 6.22 | 0.56 (0.32-0.79) |
|  | PI = 1.43% | 94 | 22 | 68 | 67 | 1.21 | 0.29 | 68 | 0.16 | 4.29 | 0.44 (0.18-0.69) |
| Huang, 2008 ^(58)^ | PPV = 11.8% | 68 | 100 | 100 | 0.80 | --- | 0.30 | 86 | 0.68 | 56.00 | 0.77 |
| Muller, 2008 ^(59)^ | CVP = 9 mmHg | 61 | 82 | 79 | 67 | 3.46 | 0.47 | 71 | 0.43 | 7.33 | 0.68 (0.50-0.83) |
|  | ITBVI = 928 ml/m^2^ | 78 | 53 | 64 | 69 | 1.65 | 0.42 | 66 | 0.31 | 3.94 | 0.64 (0.46-0.80) |
| Feissel, 2007 ^(60)^ | PPV = 12% | 100 | 70 | 86 | 100 | 3.33 | 0.00 | 89 | 0.70 | 84.00 | 0.94 ± 0.05 |
|  | PVI = 14% | 94 | 80 | 89 | 89 | 4.72 | 0.07 | 89 | 0.74 | 68.00 | 0.94 ± 0.05 |
| Lafanechère, 2006 ^(61)^ | ΔABF_PLR_ = 8% | 90 | 83 | 82 | 91 | 5.40 | 0.12 | 86 | 0.73 | 45.00 | 0.95 ± 0.04 |
|  | PPV = 12% | 70 | 92 | 87 | 78 | 8.40 | 0.33 | 82 | 0.62 | 25.67 | 0.78 ± 0.12 |
|  | LVETc = 245 ms | 70 | 67 | 60 | 66 | 2.10 | 0.45 | 68 | 0.37 | 4.67 | 0.29 ± 0.12 |
| De Backer, 2005 ^(62)^ | PPV = 11.8% | 60 | 74 | 74 | 61 | 2.34 | 0.53 | 67 | 0.34 | 4.40 | 0.76 ± 0.06 |
| Feissel, 2005 ^(63)^ | PPV = 17% | 85 | 100 | 100 | 82 | --- | 0.15 | 91 | 0.85 | 99.00 | 0.96 ± 0.03 |
|  | ∆PEP_KT_ = 4% | 92 | 89 | 92 | 89 | 8.31 | 0.09 | 91 | 0.81 | 96.00 | 0.97 ± 0.03 |
|  | ∆PEP_PLET_ = 4% | 100 | 67 | 81 | 100 | 3.00 | 0.00 | 86 | 0.67 | 52.00 | 0.94 ± 0.05 |
| Monnet, 2005 ^(64)^ | FTc = 277 ms | 55 | 94 | 92 | 65 | 99.00 | 0.48 | 74 | 0.49 | 20.78 | 0.76 ± 0.08 |
|  | ∆ABF = 18% | 90 | 94 | 95 | 89 | 16.20 | 0.11 | 92 | 0.84 | 153.00 | 0.93 ± 0.04 |
|  | ΔVpeak_aor_ = 13% | 80 | 72 | 76 | 76 | 2.88 | 0.28 | 76 | 0.52 | 10.40 | 0.82 ± 0.07 |
| Barbier, 2004 ^(65)^ | ∆IVC = 18% | 90 | 90 | 90 | 90 | 9.00 | 0.11 | 90 | 0.80 | 81.00 | 0.91 ± 0.07 |
|  | CVP = 12 mmHg | 90 | 30 | 56 | 75 | 1.29 | 0.33 | 60 | 0.20 | 3.86 | 0.57 ± 0.13 |
| Feissel, 2004 ^(66)^ | ΔIVC = 12% | 88 | 96 | 93 | 92 | 20.13 | 0.13 | 92 | 0.84 | 154.00 | --- |
| Vieillard-Baron, 2004 ^(67)^ | ΔSVC = 36% | 90 | 100 | 100 | 96 | --- | 0.10 | 97 | 0.90 | 828.00 | 0.99 ± 0.01 |
|  | PPV = 12% | 90 | 87 | 75 | 95 | 6.90 | 0.12 | 88 | 0.77 | 60.00 | 0.94 ± 0.04 |
| Feissel, 2001 ^(68)^ | ΔVpeak_aor_ = 12% | 100 | 89 | 91 | 100 | 9.00 | 0.00 | 95 | 0.89 | 160.00 | --- |
| Michard, 2000 ^(69)^ | PPV = 13% | 94 | 96 | 94 | 96 | 22.50 | 0.07 | 95 | 0.90 | 345.00 | 0.98 ± 0.03 |

**Table AF 3.** ΔABF_PLR_ = aortic blood flow variation induced by passive leg raising. BNP = B-type natriuretic peptide. CI = cardiac index. ΔCI = cardiac index variation. ΔCI_100_ = ΔCI induced by 100ml of volume expansion. ΔCI_EEO_ = ΔCI induced by end-expiratory occlusion. ΔCI_EEOcrs>30_ = ΔCI_EEO_ with compliance of the respiratory system higher than 30. ΔCI_EEOcrs≤30_ = ΔCI_EEO_ with compliance of the respiratory system lower or equal than 30. ΔCI_EEO+EIO_ =ΔCI induced by end-expiratory occlusion and end-inspiratory occlusion. ∆CI_EIO_ = ΔCI induced by end-inspiratory occlusion. ΔCI_PLR_ = ΔCI induced by passive leg raising. ΔCI_PLRcrs>30_ = ∆CI_PLR_ with compliance of the respiratory system higher than 30. ΔCI_PLRcrs≤30_ = ∆CI_PLR_ with compliance of the respiratory system lower or equal than 30. ΔCI_TREND_ = ΔCI induced by trendelenburg maneuver. ΔCO = cardiac output variation. ΔCO_PLR_ = ΔCO induced by passive leg raising. CVP = central venous pressure. DP = driving pressure (calculated as the difference between plateau pressure and PEEP). Ecw = chest wall elastance. Ers = total respiratory system elastance. ΔEtCO_2_ = end-tidal carbon dioxide variation. ΔEtCO_2PLR_ = ΔEtCO_2_ induced by passive leg raising. FTc = corrected flow time. GEDVI = global end diastolic volume index. HR: heart rate. IDVGI = initial distribution volume of glucose index. ΔIJV = internal jugular vein variation. I.V. = intravenous. ITBVI = intrathoracic blood volume index. ΔIVC = inferior vena cava variation. ΔIVC_VT6_ = ΔIVC with tidal volume of 6 ml/kg. ΔIVC_VT9_ = ΔIVC with tidal volume of 9 ml/kg. ΔIVC_VT12_ = ΔIVC with tidal volume of 12 ml/kg. LR- = negative likelihood ratio. LR+ = positive likelihood ratio. LVETc = left ventricular ejection time corrected for heart rate. MAP = mean arterial pressure. PCWP = pulmonary capillary wedge pressure. ∆PEP_KT_ = pre-ejection period of the radial arterial pressure curve variation. ∆PEP_PLET_ = pre-ejection period pulse of the plethysmographic curve variation. PI = perfusion index. PI_EAR_ = PI measured at the ear. PI_FOREHEAD_ = PI measured at the forehead. ΔPOP = pulse oximetry plethysmographic waveform amplitude variation. PPV = pulse pressure variation. PPV_7ML/KG_ = PPV induced by 7ml/kg volume expansion. PPV_100_ = PPV induced by 100-mL volume expansion. PPVa = PPV with automated measurement. PPV_crs>30_ = PPV with compliance of the respiratory system higher than 30. PPV_crs≤30_ = PPV with compliance of the respiratory system lower or equal than 30. PPV_EEO_ = PPV induced by end-expiratory occlusion. PPV_LRM_ = PPV induced by lung recruitment maneuvers. PPVni = PPV non-invasive. PPV_PLR_ = PPV induced by passive leg raising. PPV_VT6_ = PPV with tidal volume of 6 ml/kg. PPV_VT6-8_ = PPV induced by increasing the V_T_ from 6 ml/kg to 8 ml/kg. PPV_VT8_ = PPV with tidal volume of 8 ml/kg. PPV_VT9_ = PPV with tidal volume of 9 ml/kg. PPV_VT12_ = PPV with tidal volume of 12 ml/kg. Ppl = pleural pressure. PV- = negative predictive value. PV+ = positive predictive value. PVI = plethysmographic variability index. PVI_EAR_ = PVI measured at the ear. PVI_FOREHEAD_ = PVI measured at the forehead. RR = respiratory rate. RSVT = automated respiratory systolic variation test. ΔSBP = systolic blood pressure variation. ΔSBP_EEO_ = ΔSBP induced by end-expiratory occlusion. SVV = stroke volume variation. SVV_7ML/KG_ = SVV induced by 7ml/kg volume expansion. SVV_EEO_ = SVV induced by end-expiratory occlusion. ΔSVC = superior vena cava variation. SVI = stroke volume index. SVVI = stroke volume index variation. SVVI_50_ = SVVI induced by 50-ml volume expansion. SVVI_100_= SVVI induced by 100-ml volume expansion. SVV_LRM_ = SVV induced by lung recruitment maneuvers. SVVI_PLR_ = SVVI induced by passive leg raising. SVV_VT6-8_ = SVV induced by increasing the V_T_ from 6 ml/kg to 8 ml/kg. SVV_VT8_ = SVV with tidal volume of 8 ml/kg. ΔVpeak = peak velocity variation. ∆Vpeak_aor_ = ΔVpeak of aorta artery. ΔVpeak_brac_ = ΔVpeak of brachial artery. ∆Vpeak_carot_ = ΔVpeak of carotid artery. ΔVpeak_EEO_ = ΔVpeak induced by end-expiratory occlusion. V_T_ = tidal volume. VTI = velocity time integral. ∆VTI = velocity time integral variation. ∆VTI_EEO_ = ∆VTI induced by end-expiratory occlusion. ∆VTI_EIO_ = ∆VTI induced by end-inspiratory occlusion. ∆VTI_EEO+EIO_ = ∆VTI induced by end-expiratory occlusion test and end-inspiratory occlusion test.

**Table AF 5.** Fluid challenge characteristics, definition of fluid responsiveness and devices adopted to define fluid responsiveness.

| Variable | Main finding | Description |
| --- | --- | --- |
| Type of fluid | Colloids solutions remain the most frequently used fluid employed for performing fluid challenge compared to crystalloids solution. | Among the 69 studies included, colloids solutions were used in 37 studies [37 of 69 (53.6%)] studies; and crystalloids solutions were used in 27 studies [27 of 69 (39.1%)]. Additionally, crystalloid or colloid was adopted in 5 studies [5 of 69 (7.2%)]. No study conducted volume expansion through blood transfusion. Colloids solutions were used in a total of 37 studies. Out of these, hydroxyethyl starch was used in 28 studies [28 of 69 (40.6%)]; gelatin was used in 3 studies [3 of 69 (4.3%)]; albumin was used in 2 studies [2 of 69 (2.9%)]; and dextran was used in1 study [1 of 69 (1.4%). Additionally, 3 studies [3 of 69 (4.3%)] did not specify the type of colloid used. Crystalloid solutions were used in a total of 27 studies. Out of these, saline solution was used in 23 studies [23 of 69 (33.3%)]; and ringer's solution or ringer lactate were used in 2 studies [2 of 69 (2.9%)]. Additionally, 2 studies [2 of 69 (2.9%)] did not specify the type of crystalloid used. |
| Amount of fluid | The most frequently amount of fluid infused for conducting a fluid challenge via I.V. administration was 500 ml. | In total, the infusion of 500 ml of fluid was used in 30 studies [30 of 69 (43.5%)]; 250 ml was used in 9 studies [9 of 69 (13.0%)]; 7 ml/kg was used in 9 studies [9 of 69 (13.0%)]; and 8 ml/kg was used in 6 studies [6 of 69 (8.7%)]. Other variations in the amount of infused fluid included: 200 ml; 250 or 500 ml; 300 ml; 500 or 1000 ml; 4ml/kg; 6 ml/kg; 10ml/kg; 15ml/kg; and 20 ml/BMI. |
| Definition of fluid responsiveness | The most frequently adopted definition for fluid responsiveness is an increase in cardiac index ≥15%. | Assessing the hemodynamic variable adopted to define fluid responsiveness, an increased cardiac index or cardiac output was used in 42 studies [42 of 69 (60.1%)]; an increase in stroke volume index or stroke volume was used in 23 studies [23 of 69 (33.3%)]. Other variations in the definition of fluid responsiveness included an increase in VTI, and an increased in aortic blood flow. Assessing the hemodynamic variable and their corresponding threshold value employed to define fluid responsiveness, 15% changes in cardiac index was used in 29 study [29 of 69 (42.0%)]; 15% changes in stroke volume index was used in 10 study [10 of 69 (14.5%)]; and 15% changes in cardiac output was adopted in 8 study [8 of 69 (11.6%)]. Other variations in the definition of hemodynamic variable and their corresponding threshold value included 10% changes in SVI; 10% changes in SV; 10% changes in CI; 15% changes in VTI; 15% changes in aortic blood flow; 15% changes in SV; 11% changes in CI; 10% changes in CO; 20% changes in SVI. |
| Devices adopted to define fluid responsiveness | The most frequently device adopted is pulse indicator continuous cardiac output (PiCCO). | Several devices are available to measure the hemodynamic variable that defines fluid responsiveness. These devices must measure at least one of the following variables before and after an appropriate fluid challenge: cardiac index, cardiac output, stroke volume, stroke volume index, VTI, or aortic blood flow. Assessing the devices used to define fluid responsiveness, PiCCO was used in 22 studies [22 of 69 (31.9%)]; transthoracic echocardiography was used in 11 studies [11 of 69 (15.9%)]; transesophageal echocardiography was used in 10 study [10 of 69 (14.5%)]. Other devices used in the context of defining fluid responsiveness included pulmonary artery catheter; FloTrac sensor^TM^; ProAQT sensor ^TM^; PiCCO or pulmonary artery catheter; pressure recording analytical method (PRAM); transesophageal or transthoracic echocardiography; pulse power analysis calibrated using lithium dilution (LiDCO)^TM^; and PiCCO or volume View^TM^. |

**Table AF 6.** The baseline value and the variation induced by the fluid challenge of heart rate, mean arterial pressure, cardiac output, cardiac index, and central venous pressure.

| Variable | Main finding | Description |
| --- | --- | --- |
| Heart rate | Baseline heart rate or the heart rate variation induced by the fluid challenge does not allow the categorization of patients as fluid responders or fluid non-responders | A total of 60 studies [60 of 69 (86.9%)] encompassing 2,512 patients and 2,871 fluid challenges (1,425 responders; and 1,446 non-responders) reported baseline heart rate between fluid responders and fluid non-responders. Of these 60 studies, 51 studies [51 of 60 (85.0%)] did not report a significant baseline heart rate difference between fluid responders and fluid non-responders. In comparison, 9 studies [9 of 60 (15.0%)] reported a higher heart rate in fluid responders compared to non-responders. To calculate baseline heart rate values before the fluid challenge, 42 studies [42 of 69 (60.8%)] provided data enabling the calculation for each group. Of these 42 studies, the mean and standard deviation of baseline heart rate were 91 ± 19 beats/minute (93 ± 20 beats/minute for fluid responders vs. 88 ± 18 beats/minute for fluid non-responders). To assess heart rate variation induced by the fluid challenge, among the 69 studies included in the analysis, 46 studies [46 of 69 (66.7%)] provided data about heart rate variation induced by the fluid challenge. Of these 46 studies, 26 studies [26 of 46 (56.5%)] reported that heart rate did not change significantly between fluid responders and fluid non-responders; 11 studies [11 of 46 (23.9%)] reported that heart rate significantly decreased in fluid responder while remaining unchanged in fluid non-responders; 8 studies [8 of 46 (17.3%)] reported that heart rates significantly decreased in fluid responder and fluid non-responders; and 1 study [1 of 46 (2.1%)] reported that heart rates significantly increased in fluid responders while remaining unchanged in fluid non-responders. |
| Mean arterial pressure | Baseline mean arterial pressure or the mean arterial pressure variation induced by the fluid challenge do not allow the categorization of patients as fluid responders or non-responders. | A total of 56 studies [56 of 69 (81.1%)] encompassing 2,351 patients and 2,710 fluid challenges (1,343 responders; and 1,367 non-responders) reported baseline mean arterial pressure between fluid responders and fluid non-responders. Of these 56 studies, 49 studies [49 of 56 (87.5%)] did not report a significant baseline mean arterial pressure difference between fluid responders and fluid non-responders; while 7 studies [7 of 56 (12.5%)] reported a lower mean arterial pressure in fluid responders compared to non-responders. To calculate baseline mean arterial pressure before the fluid challenge, 39 studies [39 of 69 (56.5%)] provided data enabling the calculation for each group. Of these 39 studies, baseline mean arterial pressure’s mean and standard deviation were 72 ± 6 mmHg (71 ± 6 mmHg for fluid responders vs. 74 ± 7 mmHg for fluid non-responders). To assess mean arterial pressure variation induced by the fluid challenge, among the 69 studies included in the analysis, 45 studies [45 of 69 (65.2%)] provided data about mean arterial pressure variation induced by the fluid challenge. Of these 45 studies, 18 studies [18 of 45 (40.0%)] reported that mean arterial pressure significantly increased in fluid responder while remaining unchanged in fluid non-responders; 16 studies [16 of 45 (35.5%)] reported that mean arterial pressure significantly increased in fluid responder and fluid non-responders; and 11 studies [11 of 45 (24.4%)] reported that mean arterial pressure did not change significantly between fluid responders and fluid non-responders. |
| Cardiac output and cardiac index | Baseline cardiac output or cardiac index does not allow the categorization of patients as fluid responders or fluid non-responders. Furthermore, the variation in cardiac output or cardiac index induced by the fluid challenge between fluid responders and non-responders will not be compared, as these hemodynamics variables are the basis of the fluid responder definition and consequently naturally differ between the two groups. | A total of 11 studies [11 of 69 (15.9%)] encompassing 473 patients and 473 fluid challenges (252 responders; and 221 non-responders) reported baseline cardiac output between fluid responders and fluid non-responders. Of these 11 studies, 6 studies [6 of 11 (54.5%)] did not report a significant baseline cardiac output difference between fluid responders and fluid non-responders; while 5 studies [5 of 11 (45.5%)] reported a lower cardiac output in fluid responders compared to non-responders. To calculate baseline cardiac output values before the fluid challenge, 7 studies [7 of 69 (10.1%)] provided data enabling the calculation for each group. Of these 7 studies, the mean and standard deviation of baseline cardiac output were 5.8 ± 0.7 L/min (5.4 ± 0.6 L/min for fluid responders vs. 6.3 ± 1.1 L/min for fluid non-responders). A total of 36 studies [36 of 69 (52.2%)] encompassing 1536 patients and 1609 fluid challenges (847 responders; and 762 non-responders) reported baseline cardiac index between fluid responders and fluid non-responders. Of these 36 studies, 27 studies [27 of 36 (75.0%)] did not report a significant baseline cardiac index difference between fluid responders and fluid non-responders; while 9 studies [9 of 36 (25.0%)] reported a lower cardiac index in fluid responders compared to non-responders. To calculate baseline cardiac index values before the fluid challenge, 26 studies [26 of 69 (37.7%)] provided data enabling the calculation for each group. Of these 26 studies, the mean and standard deviation of baseline cardiac index were 3.1 ± 0.9 L/min/m^2^ (3.0 ± 0.8 L/min/m^2^ for fluid responders vs. 3.4 ± 1 L/min/m^2^ for fluid non-responders). |
| Central venous pressure | Baseline CVP or the CVP variation induced by the fluid challenge do not allow the categorization of patients as fluid responders or fluid non-responders. | A total of 36 studies [36 of 69 (52.2%)] encompassing 1,499 patients and 1,580 fluid challenges (834 responders; and 746 non-responders) reported baseline CVP between fluid responders and fluid non-responders. Of these 36 studies, 30 studies [30 of 36 (83.3%)] did not report a significant baseline CVP difference between fluid responders and fluid non-responders; while 6 studies [6 of 36 (16.7%)] reported a lower CVP in fluid responders compared to non-responders. To calculate baseline CVP before the fluid challenge, 25 studies [25 of 69 (36.2%)] provided data enabling the calculation for each group. Of these 25 studies, the mean and standard deviation of baseline CVP were 10±2 mmHg (9±2 mmHg for fluid responders vs. 10±3 mmHg for fluid non-responders). To assess CVP variation induced by the fluid challenge, among the 69 studies included in the analysis, 25 studies [25 of 69 (36.2%)] provided data about CVP variation induced by fluid challenge. Of these 25 studies, 18 studies [18 of 25 (72.0%)] reported that CVP significantly increased in fluid responder and in fluid non-responders; 5 studies [5 of 25 (20.0%)] reported that CVP did not change significantly between fluid responders and fluid non-responders; and 2 studies [2 of 25 (8.0%)] reported that CVP significantly increased in fluid responder while remaining unchanged in fluid non-responders. |

**Reference**

1. De Courson H, Chauvet J, Le Gall L, Georges D, et al. Utility of changes in end-tidal carbon dioxide after volume expansion to assess fluid responsiveness in the operating room: a prospective observational study. British journal of anaesthesia. 2020;125(5):672-9.
2. Wang J, Zhou D, Gao Y, Wu Z, et al. Effect of VTILVOT variation rate on the assessment of fluid responsiveness in septic shock patients. Medicine. 2020;99(47):e22702-e.
3. Weil G, Motamed C, Monnet X, Eghiaian A, et al. End-Expiratory Occlusion Test to Predict Fluid Responsiveness Is Not Suitable for Laparotomic Surgery. Anesthesia and analgesia. 2020;130(1):151-8.
4. He F, Li X, Thapa S, Li C, et al. Evaluation of volume responsiveness by pulse pressure variability and inferior vena cava dispensability index at different tidal volumes by mechanical ventilation. Braz J Med Biol Res. 2019;52(9):e8827.
5. Messina A, Montagnini C, Cammarota G, Giuliani F, et al. Assessment of Fluid Responsiveness in Prone Neurosurgical Patients Undergoing Protective Ventilation: Role of Dynamic Indices, Tidal Volume Challenge, and End-Expiratory Occlusion Test. Anesthesia and analgesia. 2019.
6. Georges D, De Courson H, Lanchon R, Sesay M, et al. End-expiratory occlusion maneuver to predict fluid responsiveness in the intensive care unit: an echocardiographic study. Critical care. 2018;22(1):32.
7. Giraud R, Abraham PS, Brindel P, Siegenthaler N, et al. Respiratory changes in subclavian vein diameters predicts fluid responsiveness in intensive care patients: a pilot study. J Clin Monit Comput. 2018;32(6):1049-55.
8. Le Guen M, Follin A, Gayat E, Fischler M. The plethysmographic variability index does not predict fluid responsiveness estimated by esophageal Doppler during kidney transplantation: A controlled study. Medicine. 2018;97(20):e10723.
9. Wang Y, Jiang Y, Wu H, Wang R, et al. Assessment of fluid responsiveness by inferior vena cava diameter variation in post-pneumonectomy patients. Echocardiography. 2018;35(12):1922-5.
10. Biais M, Lanchon R, Sesay M, Le Gall L, et al. Changes in Stroke Volume Induced by Lung Recruitment Maneuver Predict Fluid Responsiveness in Mechanically Ventilated Patients in the Operating Room. Anesthesiology. 2017;126(2):260-7.
11. Biais M, Larghi M, Henriot J, De Courson H, et al. End-Expiratory Occlusion Test Predicts Fluid Responsiveness in Patients With Protective Ventilation in the Operating Room. Anesthesia and analgesia. 2017;125(6):1889-95.
12. Biais M, De Courson H, Lanchon R, Pereira B, et al. Mini-fluid Challenge of 100 ml of Crystalloid Predicts Fluid Responsiveness in the Operating Room. Anesthesiology. 2017;127(3):450-6.
13. Jozwiak M, Depret F, Teboul JL, Alphonsine JE, et al. Predicting Fluid Responsiveness in Critically Ill Patients by Using Combined End-Expiratory and End-Inspiratory Occlusions With Echocardiography. Critical care medicine. 2017;45(11):e1131-e8.
14. Lu N, Xi X, Jiang L, Yang D, et al. Exploring the best predictors of fluid responsiveness in patients with septic shock. Am J Emerg Med. 2017;35(9):1258-61.
15. Myatra SN, Prabu NR, Divatia JV, Monnet X, et al. The Changes in Pulse Pressure Variation or Stroke Volume Variation After a "Tidal Volume Challenge" Reliably Predict Fluid Responsiveness During Low Tidal Volume Ventilation. Critical care medicine. 2017;45(3):415-21.
16. Yonis H, Bitker L, Aublanc M, Perinel Ragey S, et al. Change in cardiac output during Trendelenburg maneuver is a reliable predictor of fluid responsiveness in patients with acute respiratory distress syndrome in the prone position under protective ventilation. Critical care. 2017;21(1):295.
17. De Broca B, Garnier J, Fischer MO, Archange T, et al. Stroke volume changes induced by a recruitment maneuver predict fluid responsiveness in patients with protective ventilation in the operating theater. Medicine. 2016;95(28):e4259.
18. Lee SH, Chun YM, Oh YJ, Shin S, et al. Prediction of fluid responsiveness in the beach chair position using dynamic preload indices. J Clin Monit Comput. 2016;30(6):995-1002.
19. Liu Y, Wei LQ, Li GQ, Yu X, et al. Pulse Pressure Variation Adjusted by Respiratory Changes in Pleural Pressure, Rather Than by Tidal Volume, Reliably Predicts Fluid Responsiveness in Patients With Acute Respiratory Distress Syndrome. Critical care medicine. 2016;44(2):342-51.
20. Wu CY, Cheng YJ, Liu YJ, Wu TT, et al. Predicting stroke volume and arterial pressure fluid responsiveness in liver cirrhosis patients using dynamic preload variables: A prospective study of diagnostic accuracy. European journal of anaesthesiology. 2016;33(9):645-52.
21. Angappan S, Parida S, Vasudevan A, Badhe AS. The comparison of stroke volume variation with central venous pressure in predicting fluid responsiveness in septic patients with acute circulatory failure. Indian J Crit Care Med. 2015;19(7):394-400.
22. Ibarra-Estrada MA, Lopez-Pulgarin JA, Mijangos-Mendez JC, Diaz-Gomez JL, et al. Respiratory variation in carotid peak systolic velocity predicts volume responsiveness in mechanically ventilated patients with septic shock: a prospective cohort study. Critical ultrasound journal. 2015;7(1):29.
23. Mallat J, Meddour M, Durville E, Lemyze M, et al. Decrease in pulse pressure and stroke volume variations after mini-fluid challenge accurately predicts fluid responsiveness†. British journal of anaesthesia. 2015;115(3):449-56.
24. Charbonneau H, Riu B, Faron M, Mari A, et al. Predicting preload responsiveness using simultaneous recordings of inferior and superior vena cavae diameters. Critical care. 2014;18(5):473.
25. Guarracino F, Ferro B, Forfori F, Bertini P, et al. Jugular vein distensibility predicts fluid responsiveness in septic patients. Critical care. 2014;18(6):647.
26. Siswojo AS, Wong DM, Phan TD, Kluger R. Pleth variability index predicts fluid responsiveness in mechanically ventilated adults during general anesthesia for noncardiac surgery. Journal of cardiothoracic and vascular anesthesia. 2014;28(6):1505-9.
27. Feissel M, Kalakhy R, Banwarth P, Badie J, et al. Plethysmographic variation index predicts fluid responsiveness in ventilated patients in the early phase of septic shock in the emergency department: a pilot study. J Crit Care. 2013;28(5):634-9.
28. Fischer MO, Pelissier A, Bohadana D, Gérard JL, et al. Prediction of responsiveness to an intravenous fluid challenge in patients after cardiac surgery with cardiopulmonary bypass: a comparison between arterial pulse pressure variation and digital plethysmographic variability index. Journal of cardiothoracic and vascular anesthesia. 2013;27(6):1087-93.
29. Freitas FG, Bafi AT, Nascente AP, Assunção M, et al. Predictive value of pulse pressure variation for fluid responsiveness in septic patients using lung-protective ventilation strategies. British journal of anaesthesia. 2013;110(3):402-8.
30. Ishihara H, Hashiba E, Okawa H, Saito J, et al. Neither dynamic, static, nor volumetric variables can accurately predict fluid responsiveness early after abdominothoracic esophagectomy. Perioperative medicine. 2013;2(1):3.
31. Monnet X, Bataille A, Magalhaes E, Barrois J, et al. End-tidal carbon dioxide is better than arterial pressure for predicting volume responsiveness by the passive leg raising test. Intensive care medicine. 2013;39(1):93-100.
32. Monnet X, Guerin L, Jozwiak M, Bataille A, et al. Pleth variability index is a weak predictor of fluid responsiveness in patients receiving norepinephrine. British journal of anaesthesia. 2013;110(2):207-13.
33. Trepte CJ, Eichhorn V, Haas SA, Stahl K, et al. Comparison of an automated respiratory systolic variation test with dynamic preload indicators to predict fluid responsiveness after major surgery. British journal of anaesthesia. 2013;111(5):736-42.
34. Vos JJ, Kalmar AF, Struys MM, Wietasch JK, et al. Comparison of arterial pressure and plethysmographic waveform-based dynamic preload variables in assessing fluid responsiveness and dynamic arterial tone in patients undergoing major hepatic resection. British journal of anaesthesia. 2013;110(6):940-6.
35. Biais M, Cottenceau V, Stecken L, Jean M, et al. Evaluation of stroke volume variations obtained with the pressure recording analytic method. Critical care medicine. 2012;40(4):1186-91.
36. Cecconi M, Monti G, Hamilton MA, Puntis M, et al. Efficacy of functional hemodynamic parameters in predicting fluid responsiveness with pulse power analysis in surgical patients. Minerva Anestesiol. 2012;78(5):527-33.
37. Fu Q, Mi WD, Zhang H. Stroke volume variation and pleth variability index to predict fluid responsiveness during resection of primary retroperitoneal tumors in Hans Chinese. Bioscience trends. 2012;6(1):38-43.
38. Haas S, Trepte C, Hinteregger M, Fahje R, et al. Prediction of volume responsiveness using pleth variability index in patients undergoing cardiac surgery after cardiopulmonary bypass. J Anesth. 2012;26(5):696-701.
39. Khwannimit B, Bhurayanontachai R. Prediction of fluid responsiveness in septic shock patients: comparing stroke volume variation by FloTrac/Vigileo and automated pulse pressure variation. European journal of anaesthesiology. 2012;29(2):64-9.
40. Monge García MI, Gil Cano A, Gracia Romero M, Monterroso Pintado R, et al. Non-invasive assessment of fluid responsiveness by changes in partial end-tidal CO2 pressure during a passive leg-raising maneuver. Annals of intensive care. 2012;2:9-.
41. Monnet X, Bleibtreu A, Ferré A, Dres M, et al. Passive leg-raising and end-expiratory occlusion tests perform better than pulse pressure variation in patients with low respiratory system compliance. Critical care medicine. 2012;40(1):152-7.
42. Monnet X, Dres M, Ferré A, Le Teuff G, et al. Prediction of fluid responsiveness by a continuous non-invasive assessment of arterial pressure in critically ill patients: comparison with four other dynamic indices. British journal of anaesthesia. 2012;109(3):330-8.
43. Oliveira-Costa CD, Friedman G, Vieira SR, Fialkow L. Pulse pressure variation and prediction of fluid responsiveness in patients ventilated with low tidal volumes. Clinics (Sao Paulo). 2012;67(7):773-8.
44. Desgranges FP, Desebbe O, Ghazouani A, Gilbert K, et al. Influence of the site of measurement on the ability of plethysmographic variability index to predict fluid responsiveness. British journal of anaesthesia. 2011;107(3):329-35.
45. Hood JA, Wilson RJ. Pleth variability index to predict fluid responsiveness in colorectal surgery. Anesthesia and analgesia. 2011;113(5):1058-63.
46. Lakhal K, Ehrmann S, Benzekri-Lefèvre D, Runge I, et al. Respiratory pulse pressure variation fails to predict fluid responsiveness in acute respiratory distress syndrome. Critical care. 2011;15(2):R85.
47. Loupec T, Nanadoumgar H, Frasca D, Petitpas F, et al. Pleth variability index predicts fluid responsiveness in critically ill patients. Critical care medicine. 2011;39(2):294-9.
48. Machare-Delgado E, Decaro M, Marik PE. Inferior vena cava variation compared to pulse contour analysis as predictors of fluid responsiveness: a prospective cohort study. Journal of intensive care medicine. 2011;26(2):116-24.
49. Moretti R, Pizzi B. Inferior vena cava distensibility as a predictor of fluid responsiveness in patients with subarachnoid hemorrhage. Neurocrit Care. 2010;13(1):3-9.
50. Muller L, Louart G, Bousquet P-J, Candela D, et al. The influence of the airway driving pressure on pulsed pressure variation as a predictor of fluid responsiveness. Intensive care medicine. 2010;36(3):496-503.
51. Zimmermann M, Feibicke T, Keyl C, Prasser C, et al. Accuracy of stroke volume variation compared with pleth variability index to predict fluid responsiveness in mechanically ventilated patients undergoing major surgery. European journal of anaesthesiology. 2010;27(6):555-61.
52. Monge Garcia MI, Gil Cano A, Diaz Monrove JC. Brachial artery peak velocity variation to predict fluid responsiveness in mechanically ventilated patients. Critical care. 2009;13(5):R142.
53. Monnet X, Osman D, Ridel C, Lamia B, et al. Predicting volume responsiveness by using the end-expiratory occlusion in mechanically ventilated intensive care unit patients. Critical care medicine. 2009;37(3):951-6.
54. Muller L, Louart G, Teboul JL, Mahamat A, et al. Could B-type Natriuretic Peptide (BNP) plasma concentration be useful to predict fluid responsiveness [corrected] in critically ill patients with acute circulatory failure? Ann Fr Anesth Reanim. 2009;28(6):531-6.
55. Vallee F, Richard JC, Mari A, Gallas T, et al. Pulse pressure variations adjusted by alveolar driving pressure to assess fluid responsiveness. Intensive care medicine. 2009;35(6):1004-10.
56. Biais M, Nouette-Gaulain K, Cottenceau V, Revel P, et al. Uncalibrated pulse contour-derived stroke volume variation predicts fluid responsiveness in mechanically ventilated patients undergoing liver transplantation. British journal of anaesthesia. 2008;101(6):761-8.
57. Cannesson M, Desebbe O, Rosamel P, Delannoy B, et al. Pleth variability index to monitor the respiratory variations in the pulse oximeter plethysmographic waveform amplitude and predict fluid responsiveness in the operating theatre. British journal of anaesthesia. 2008;101(2):200-6.
58. Huang CC, Fu JY, Hu HC, Kao KC, et al. Prediction of fluid responsiveness in acute respiratory distress syndrome patients ventilated with low tidal volume and high positive end-expiratory pressure. Critical care medicine. 2008;36(10):2810-6.
59. Muller L, Louart G, Bengler C, Fabbro-Peray P, et al. The intrathoracic blood volume index as an indicator of fluid responsiveness in critically ill patients with acute circulatory failure: a comparison with central venous pressure. Anesthesia and analgesia. 2008;107(2):607-13.
60. Feissel M, Teboul JL, Merlani P, Badie J, et al. Plethysmographic dynamic indices predict fluid responsiveness in septic ventilated patients. Intensive care medicine. 2007;33(6):993-9.
61. Lafanechère A, Pène F, Goulenok C, Delahaye A, et al. Changes in aortic blood flow induced by passive leg raising predict fluid responsiveness in critically ill patients. Critical care. 2006;10(5):R132-R.
62. De Backer D, Heenen S, Piagnerelli M, Koch M, et al. Pulse pressure variations to predict fluid responsiveness: influence of tidal volume. Intensive care medicine. 2005;31(4):517-23.
63. Feissel M, Badie J, Merlani PG, Faller JP, et al. Pre-ejection period variations predict the fluid responsiveness of septic ventilated patients. Critical care medicine. 2005;33(11):2534-9.
64. Monnet X, Rienzo M, Osman D, Anguel N, et al. Esophageal Doppler monitoring predicts fluid responsiveness in critically ill ventilated patients. Intensive care medicine. 2005;31(9):1195-201.
65. Barbier C, Loubieres Y, Schmit C, Hayon J, et al. Respiratory changes in inferior vena cava diameter are helpful in predicting fluid responsiveness in ventilated septic patients. Intensive care medicine. 2004;30(9):1740-6.
66. Feissel M, Michard F, Faller JP, Teboul JL. The respiratory variation in inferior vena cava diameter as a guide to fluid therapy. Intensive care medicine. 2004;30(9):1834-7.
67. Vieillard-Baron A, Chergui K, Rabiller A, Peyrouset O, et al. Superior vena caval collapsibility as a gauge of volume status in ventilated septic patients. Intensive care medicine. 2004;30(9):1734-9.
68. Feissel M, Michard F, Mangin I, Ruyer O, et al. Respiratory changes in aortic blood velocity as an indicator of fluid responsiveness in ventilated patients with septic shock. Chest. 2001;119(3):867-73.
69. Michard F, Boussat S, Chemla D, Anguel N, et al. Relation between respiratory changes in arterial pulse pressure and fluid responsiveness in septic patients with acute circulatory failure. American journal of respiratory and critical care medicine. 2000;162(1):134-8.
